# Visualizing the Invisible: Dual Click Imaging of Ruthenium-Based Photoactivated Chemotherapy Agents and Their DNA Synthesis Inhibition in Fixed Cancer Cells

**DOI:** 10.1021/jacs.5c13249

**Published:** 2025-11-05

**Authors:** Anja Busemann, Lisa Rieger, Rachael M. Cunningham, Sam C. Davidse, Irene Regeni, Ingrid Flaspohler, Claudia Schmidt, Xue-Quan Zhou, Vincent van Rixel, Maxime A. Siegler, Ingo Ott, Sylvia E. Le Dévédec, Hans-Achim Wagenknecht, Victoria J. DeRose, Sylvestre Bonnet

**Affiliations:** † Leiden Institute of Chemistry, Leiden University, Einsteinweg 55, 2333 CC Leiden, Netherlands; ‡ Institute of Organic Chemistry, Karlsruhe Institute of Technology (KIT), Fritz-Haber-Weg 6, 76131 Karlsruhe, Germany; § Department of Chemistry and Biochemistry, 3265 University of Oregon, Eugene, Oregon 97403, United States; ∥ Leiden Academic Centre for Drug Research (LACDR), 4496 Leiden University, Einsteinweg 55, 2333 CC Leiden, Netherlands; ⊥ Small Molecule X-Ray Facility, Department of Chemistry, 1466 Johns Hopkins University, Baltimore, Maryland 21218, United States; # Institute of Medicinal and Pharmaceutical Chemistry, Technische Universität Braunschweig, Beethovenstrasse 55, 38106 Braunschweig, Germany

## Abstract

Like many drugs, ruthenium-based photoactivated chemotherapy (PACT) complexes are hard to follow in cells due to their absence of emissive properties. Here, two alkyne-functionalized Ru-based PACT compounds with the formula [Ru­(HCC-tpy)­(N̂N)­(Hmte)]­(PF_6_)_2_ were synthesized, where HCC-tpy = 4′-ethynyl-2,2′:6′,2″-terpyridine, N̂N = 3,3′-biisoquinoline (i-biq, [**2**]­(PF_6_)_2_) or di­(isoquinolin-3-yl)­amine (i-Hdiqa, [**4**]­(PF_6_)_2_), and Hmte = 2-(methylthio)­ethanol. Their challenging synthesis involved a protection–deprotection strategy to avoid the reaction of the free alkyne group with the coordinatively unsaturated ruthenium center. The thermal stability and photosubstitution quantum yield (Φ_[**2**]_ = 0.022 and Φ_[**4**]_ = 0.080) of the PACT complexes were essentially preserved upon alkyne functionalization. Interestingly, however, cellular uptake was doubled after alkyne functionalization, resulting in increased cytotoxicity against A549 cancer cells for both complexes in the dark and after green light activation (EC_50,light_ = 5 and 7 μM, respectively). To follow the complexes and see the effect of light activation, post-treatment fluorophore labeling via copper-catalyzed azide–alkyne cycloaddition was realized in fixed cells at 2 different time points, which allowed for imaging the otherwise invisible molecules. The images showed that the Ru complexes accumulated in the cytoplasm only after light irradiation and that they colocalized with the lysosomes and the Golgi apparatus. Moreover, we combined this approach with metabolic labeling of DNA, and showed by dual click imaging that DNA replication was inhibited by complex **4**. The strategy described herein, pioneered for nonemissive, photosubstitutionally active ruthenium complexes, opens a new avenue for investigating the selective attack of lung cancer cells by PACT.

## Graphical abstract

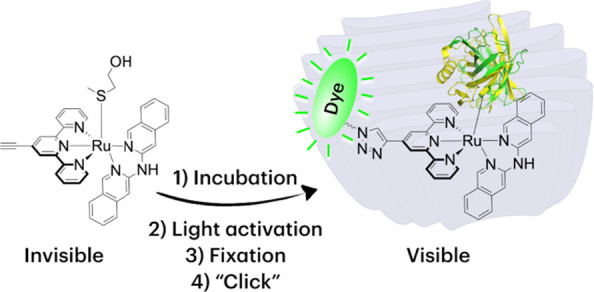

## Introduction

Light-driven therapy has generated much attention in recent years due to different clinical breakthroughs brought by photodynamic therapy (PDT) and photodynamic diagnosis (PDD) for the treatment of cancer. For instance, 5-amino levulinic acid (5-ALA) was recently approved by the FDA for blue-light visualization of brain tumors during excision surgery^1^ and is in clinical trial for red-light phototherapy,^2,3^ while TLD-1433 is currently in clinical trial phase II for the green-light phototherapy treatment of refractive bladder cancer with low side effects for the patients.^4–6^ A comparatively newer approach called photoactivated chemotherapy (PACT) also brings new promise in photomedicine because it has the potential to attack hypoxic tumors without tissue necrosis, as light activation of the prodrug is obtained without Reactive Oxygen Species (ROS) generation by an oxygen-independent bond cleavage photoreaction. In PACT, a biologically active compound is protected by a light-cleavable protecting group that inactivates its function. Upon local light activation inside the tumor, the protecting group is photoreleased, thereby releasing the toxic species that can resume its interaction with its intended target.

While the photosubstitution properties and anticancer properties of ruthenium-based PACT agents have been studied extensively,^6–17^ their behavior in the complex environment of the cell remains rather unexplored due to a poorly discussed but constant issue of this family of molecules: they are usually not emissive. To gather information about the fate of a drug in biology, such as its intracellular target(s), it is very convenient to have emissive drugs. With such compounds, the mode of action can be more easily correlated to the efficacy and cytotoxicity profile, enabling improvement of the drug design and increasing its chances to follow in vitro and in vivo as well as getting into the clinics. While some clinically approved or tested drugs, such as doxorubicin, temoporfin, or TLD-1433, are emissive, many others are not. This is the case for most ruthenium-based PACT compounds.

The low emission observed for photosubstitutionally active ruthenium-based PACT prodrugs is due to the competition between the different triplet excited state decay processes, leading to photosubstitution, phosphorescence, or singlet oxygen production. The photosubstitution reaction observed in PACT prodrugs usually results in luminescence quenching, as the photogenerated triplet metal-to-ligand charge transfer (^3^MLCT) excited states generated photochemically upon spin flip usually decay via the generation of low-lying triplet metal-centered (^3^MC) excited states that may lead to ligand substitution. A common method to visualize nonemissive (pro)­drugs in cells is to conjugate them with an organic fluorophore.^18^ The first example of a metal-based drug that could be traced in cells is a cisplatin derivative covalently bound to an emissive carboxyfluorescein diacetate (CFDA) moiety reported by Molenaar et al.^19^ They confirmed the accumulation of the platinum compound in the nucleus, as usually expected for cisplatin. Hereafter, other groups investigated fluorophore-labeled drug derivatives,^20–24^ and PACT compounds covalently bound to anthraquinone or anthracene and have shown interesting emission properties.^25,26^ However, in these conjugates, it is never clear how functionalization with the organic dye modifies the biodistribution and overall biological properties of the drug.^27,28^ Notably, many fluorophore moieties drastically change the hydrophobicity of the conjugate, which modifies its cellular uptake and intracellular distribution compared with the original (pro)­drug.^29^ In addition, due to its size and/or charge, the fluorophore moiety might also strongly modify the interaction of the conjugate with its biological target, leading to a mode of action that does not reflect that of the original (pro)­drug.^30^ These effects culminate in photoactivated drugs, where interactions in the excited state between the photoactive core and the appended fluorophore are very likely.

An alternative method for the visualization of nonemissive organic drugs in cells was recognized by the Nobel Prize in 2022 to Sharpless, Meldal, and Bertozzi.^31^ This method is based on the smallest possible modification of the drug using a minimal functional group called a “click handle,” and on postlabeling the drug in its cellular environment after cell fixation using “click” chemistry employing a fluorophore functionalized with a selective partner to the click handle. This method usually assumes that the click handle can be small enough to preserve the chemical and biological properties of the unfunctionalized drug. In this assumption, the cellular uptake, intracellular distribution, and target interaction of the drug are minimally affected by the click handle, while the fluorophore moiety is installed after the drug has interacted with biomolecules in the cell.

In fact, more than one option of reactive click partners and their associated reaction conditions have been developed throughout the years.^32^ The copper­(I)-catalyzed azide–alkyne cycloaddition (CuAAC),^33^ the strain-promoted azide–alkyne cycloaddition (SPAAC),^34^ the inverse electron-demand Diels–Alder reaction (IEDDA),^35^ and the photoclick reaction^36–38^ belong to the most well-known click reactions. These methods were simultaneously applied for metallodrug research by DeRose and Bierbach.^30,39^ The presence of a metal center in a drug makes this approach challenging, as it may lead to interfering interaction with the click handle, and so far, only the groups of Bierbach,^29^ DeRose and co-workers,^40^ Che and co-workers,^41,42^ and Griffith^43^ have used click chemistry methods to study the cellular distribution of metallodrugs. They all used the CuAAC strategy that employs a free alkyne (3-atom) as a click handle and an azide as a reactive partner. To our knowledge, these methods have not yet been used for studying ruthenium-based PACT prodrugs due to the double challenge they represent: a synthetic challenge, consisting of functionalizing photolabile ruthenium complexes with free alkyne groups and a photochemical challenge, due to the possible interaction that may take place between the alkyne, the clicked fluorophore, and the photoactive ruthenium center.

In this work, we functionalized two previously described ruthenium-based PACT agents activated by green light, [Ru­(tpy)­(i-biq)­(Hmte)]­(PF_6_)_2_ [**1**]­(PF_6_)_2_ and [Ru(tpy)(i-Hdiqa)(Hmte)]­(PF_6_)_2_ [**3**]­(PF_6_)_2_ (where tpy = 2,2′:6′,2″-terpyridine, i-biq = 3,3′-biisoquinoline, i-Hdiqa = di­(isoquinolin-3-yl)­amine, and Hmte = 2-(methylthio)­ethanol), with the smallest click handle possible, that is, a simple free alkyne group (Figure 1, red label), to obtain the drug analogues [Ru­(HCC-tpy)­(N̂N)­(Hmte)]­(PF_6_)_2_, where N̂N = i-biq ([**2**]­(PF_6_)_2_) or i-Hdiqa ([**4**]­(PF_6_)_2_, see Figure 2).^44,45^ The design of these PACT agents was motivated by three factors. First, we wanted to investigate whether free alkyne functionalization of the PACT agent was “minimal,” that is, whether it would influence its photochemical and biological properties. Second, we wanted to investigate if “click” fluorophore labeling with azide-functionalized fluorescent dyes in fixed cells could be done to observe in cellulo, and if possible in a time-dependent manner, the otherwise invisible PACT compounds. Third, we wanted to localize PACT molecules inside the cell by performing colocalization experiments. The principle of our approach is summarized in Figure 1: the alkyne-functionalized PACT complex is first incubated in cells, then activated with light, then further incubated in the dark for a fixed duration, then fixed, and finally, clicked for confocal fluorescent imaging.

**Figure 1:**
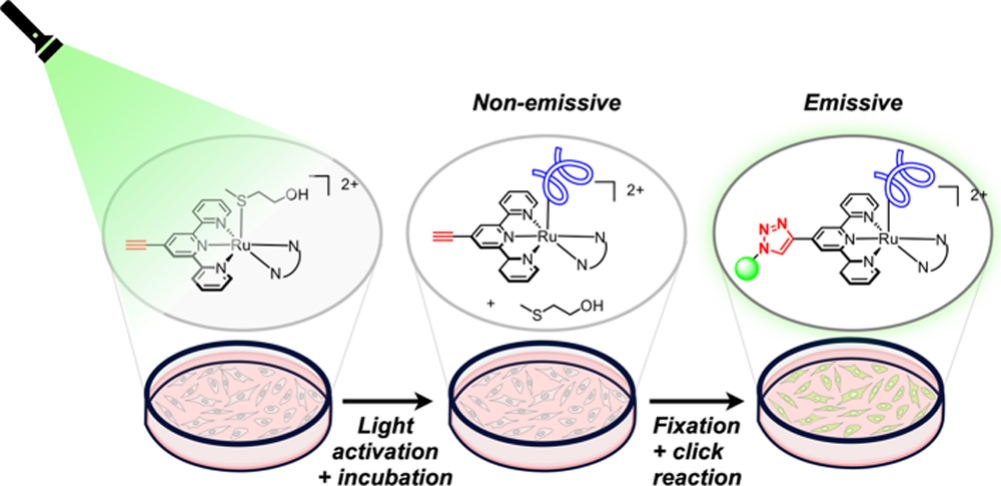
Principles for the time-dependent imaging of nonemissive ruthenium-based PACT compounds in cancer cells. Cells are treated with an alkyne-modified PACT agent for a specific time interval and activated with light. Upon exposure to light, the thioether ligand is cleaved off and the complex binds to its unknown biological target(s) (in blue). Subsequently, cells are fixed and postsynthetically labeled via click chemistry of an azide-functionalized fluorophore, before imaging.

## Results

The alkyne-functionalized PACT agents [**2**]^2+^ and [**4**] ^2+^ (Figure 2) were synthesized according to the synthetic route developed for their nontoxic analogue [Ru­(HCC-tpy)­(bpy)­(Hmte)]­(PF_6_)_2_ (where bpy = 2,2′-bipyridine, Scheme S1).^44^ In short, the terminal alkyne was protected with a *tert*-butyldimethylsilyl (TBDMS) group during all steps of the synthesis of the ruthenium PACT complex. Such protection prevents the reaction between the terminal alkyne and the coordination site(s) on the metal center opening during ligand installation, which would result in the formation of undesired polymerization side products that are difficult to remove. TBDMS was selectively removed at the very end of the synthesis on the thioether-protected ruthenium center using an excess of potassium fluoride to afford the two CCH-functionalized thioether-bound prodrugs as brown reddish salts in moderate to high yield ([**2**]­(PF_6_)_2_: 62%; [**4**]­(PF_6_)_2_: 83%). ^1^H NMR spectroscopy in acetone-d_6_ confirmed successful deprotection and the formation of the free alkyne via the appearance of a singlet at 4.56 and 4.52 ppm for [**2**]­(PF_6_)_2_ and [**4**]­(PF_6_)_2_, respectively (Figures S13 and S19). Due to the low water solubility of [**2**]­(PF_6_)_2_, an ion exchange procedure was applied to exchange the PF_6_^–^ counterions for Cl^–^, thus affording [**2**]­Cl_2_ (Figures S16–S18). Full characterization is provided in the Supporting Information (Figures S16–S21).

**Figure 2:**
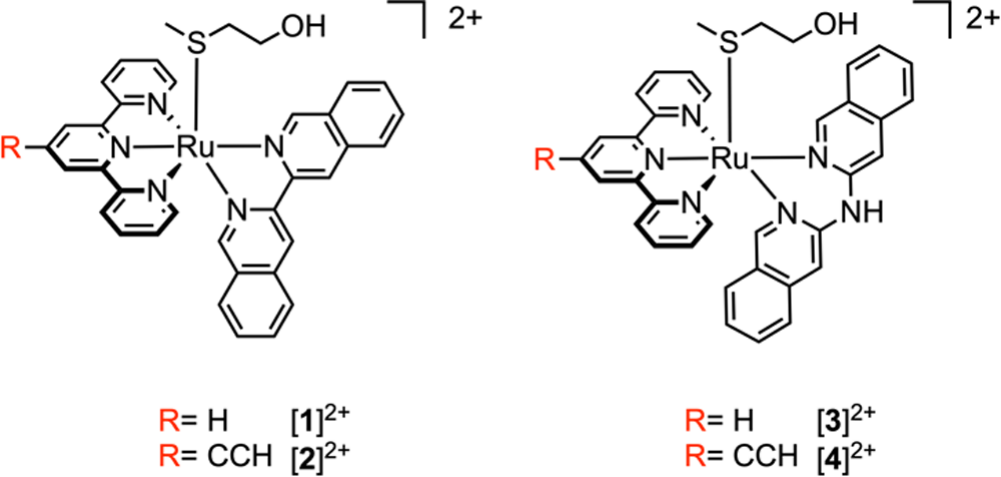
Chemical structures of the ruthenium-based PACT agents [**1**]^2+^–[**4**]^2+^.

Single crystals suitable for X-ray structure determination were obtained for the complex [**2**]­(PF_6_)_2_ by slow vapor diffusion of diethyl ether into a solution of the complex in cyclopentane. The molecular geometry of the complex in the crystal structure is shown in Figure 3. Selected bond lengths and angles are summarized in Table 1, together with those reported for the alkyne-free complex [**1**]­(PF_6_)_2_.^45^ In [**2**]^2+^, the terminal alkyne has a bond length (C≡C) of 1.188(7) Å, which is in agreement with the literature.^46^ The Ru–N bond lengths of polypyridyl ligands tpy and *i-*biq are not significantly different in complexes [**2**]^2+^ and [**1**]^2+^. The length of the Ru–S bond with the thioether ligand is also not affected by alkyne functionalization (Ru–S = 2.3623(10) and 2.368(3) Å for [**2**]^2+^ and [**1**]^2+^, respectively).^44^ Since crystal growth for complex [**4**]^2+^ was unsuccessful, we performed density functional theory (DFT) modeling to obtain the complex structure and compare it to the optimized DFT geometry of [**3**]^2+^ (Table 1).^45^ The DFT calculation revealed no significant differences between the geometries of [**4**]^2+^ and [**3**]^2+^. Overall, the addition of the alkyne moiety to the tpy ligand has no significant effect on the bond lengths and overall geometry of these ground-state ruthenium complexes.

**Figure 3:**
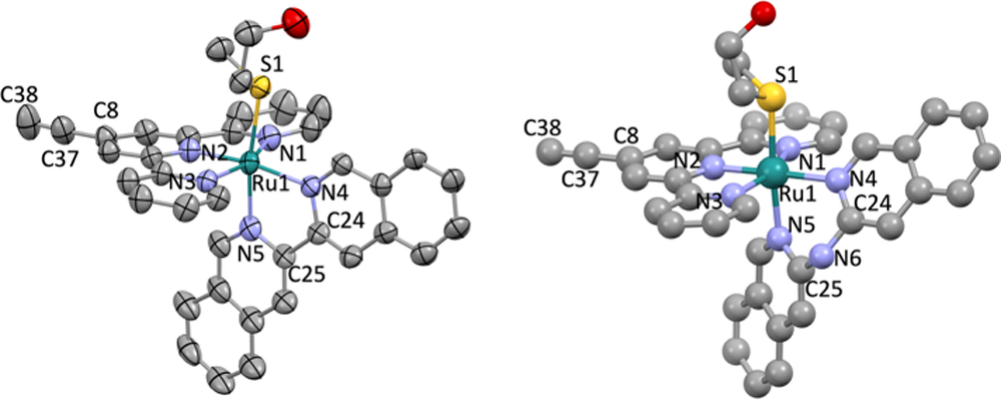
Left: Displacement ellipsoid (50% probability level) of the cationic part as observed in the crystal structure of [**2**]­(PF_6_)_2_ at 110(2) K. Disorder, counterions, and H atoms have been omitted for clarity. Right: DFT model of [**4**]^2+^.

**Table 1:** Selected Bond Lengths (Å) and Angles (°) in the X-ray Structures of [**1**]­PF_6__2_ and [**2**]­PF_6__2_, and in the DFT Model of [**3**]^2+^ and [**4**]^2+^

	[1](PF_6_)_2_ ^a^	[2](PF_6_)_2_	[3]^2+^ ^a^,^b^	[4]^2+^ ^b^
Ru–N1	2.071(9)	2.086(3)	2.095	2.098
Ru–N2	1.967(10)	1.963(3)	1.978	1.974
Ru–N3	2.073(10)	2.073(3)	2.114	2.111
Ru–N4	2.104(10)	2.093(3)	2.138	2.141
Ru–N5	2.074(9)	2.069(6)	2.115	2.112
Ru–S1	2.368(3)	2.3623(10)	2.396	2.402
C8–C37		1.435(6)		1.423
C37–C38		1.188(7)		1.202
N1–Ru1–N2	79.3(4)	79.61(13)	79.17	79.13
N2–Ru1–N3	80.1(4)	79.59(13)	78.90	79.01
N1–Ru1–N3	159.4(4)	159.17(13)	158.01	158.10
N4–Ru1–N5	79.4(4)	79.7(4)	86.45	86.47
λ^c^	3.65	2.73	2.46	3.63
σ^2^ ^d^	60.3	59.8	46.4	46.1

a Data from Busemann et al.^45^

b Data from DFT calculations.

c Mean quadratic elongation $\lambda =\frac{1}{6}\sum_{n=1,6}{\left[\frac{d_{n}-\langle d\rangle }{\left\langle d\right\rangle }\right]}^{2}$ , where *d_n_* is one of the six bond lengths in the first coordination sphere, and ⟨*d*⟩ is the mean of those bond lengths.

d Bond angle variance $\sigma^{2}=\frac{1}{11}\sum_{n=1,12}(\theta_{n}-90{)}^{2}$ , where θ_*n*_ is one of the 12 angles in the first coordination sphere.

The UV–vis absorption in aqueous solution showed that the two complexes [**2**]^2+^ and [**4**]^2+^ have ^1^MLCT bands at 470 and 485 nm, compared to the nonfunctionalized analogues [**1**]^2+^ and [**3**]^2+^, which have absorption maxima at 429 and 470 nm, respectively (Table 2 and Figure S22).^45^ Thus, the alkyne functionalization caused a bathochromic shift of the ^1^MLCT absorption in both complexes. The alkynyl complexes showed very little singlet oxygen generation (quantum yield Φ_Δ_ < 0.03), and their phosphorescence quantum yields were found to be very low as well (Φ_P_ < 5 × 10^–4^, see Table 2 and Figure S23), which makes them typical PACT compounds like their alkyne-free analogues.

**Table 2:** Lowest-Energy Absorption Maxima (λ_max_ in nm), Molar Absorption Coefficients at λ_max_ (ε_max_ in M^–1^ cm^–1^), Phosphorescence Quantum Yields (Φ_P_), Singlet Oxygen Quantum Yields (Φ_Δ_), and Photosubstitution Quantum Yields (Φ_517_) for Complexes [**1**]^2+^–[**4**]^2+^

complex	N̂N	R	λ_max_ (ε_max_)^a^	Φ_P_ ^b^	Φ_Δ_ ^b^	Φ_517_ ^a^
[1]^2+^ ^c^	*i*-biq	H	429 (5.76 × 10^3^)	1.5 × 10^–4^	0.010	0.023
[2]^2+^	*i*-biq	CCH	470 (7.65 × 10^3^)	2.4 × 10^–4^	0.017	0.022
[3]^2+^ ^c^	*i*-Hdiqa	H	470 (5.35 × 10^3^)	4.5 × 10^–4^	0.042	0.077
[4]^2+^	*i*-Hdiqa	CCH	485 (6.86 × 10^3^)	<1 × 10^–4^	0.010	0.080

a In Milli-Q water.

b In methanol-d_4_.

c Data from Busemann et al.^45^

The photoreactivity of [**2**]^2+^ and [**4**]^2+^ was investigated by green light irradiation (520 nm) of low mM solutions of the complexes in water at 37 °C followed by UV–vis spectroscopy (Figure S24). Each complex exhibited a bathochromic shift of their absorption maxima after irradiation, which is indicative of the release of the thioether ligand to form the corresponding aqua complex, as also demonstrated by mass spectrometry (*m*/*z* = 316.4 (calc. *m*/*z* = 316.5 for [Ru(HCC-tpy)(i-biq)(OH_2_)]^2+^) and *m*/*z* = 323.6 (calc. *m*/*z* = 324.1 for [Ru(HCC-tpy)(i-Hdiqa)(OH_2_)]^2+^, see Figure S25).^47–49^ The photosubstitution quantum yields (Φ_517_) were determined by deconvolution of the UV–vis absorption data using the Glotaran software package.^50^ Φ_517_ values of 0.022 and 0.080 were obtained for [**2**]^2+^ and [**4**]^2+^, respectively (Table 2 and Figure S26), which are comparable with the values of 0.023 and 0.077 reported for complexes [**1**]^2+^ and [**3**]^2+^.^45^ Overall, all photochemical investigations confirmed the negligible influence of the alkyne group on the photosubstitution properties of the complexes. The excellent photosubstitution quantum yields of [**2**]^2+^ and [**4**]^2+^ coupled to their excellent thermal stability in cell-growing medium (OptiMEM complete) when kept in the dark at 37 °C for 24 h (Figure S28), indicated that both alkynyl complexes should behave as PACT agents that are similar to parent compounds [**1**]^2+^ and [**3**]^2+^.

To compare the cytotoxicities of the complexes, [**2**]^2+^ and [**4**]^2+^ were then tested under normoxic conditions (21% O_2_) in human lung carcinoma (A549) and human epidermoid carcinoma (A431) cell lines. As described in earlier protocols,^51^ the complexes were incubated for 24 h in the dark at various concentrations prior to green light activation (520 nm, 38 J/cm^2^, 30 min, see Figure S29). The activated complexes were further incubated for 48 h before measuring the relative cell proliferation with a sulforhodamine B (SRB) end-point assay.^51^ The dose–response curves are shown in Figure S30. The effective concentrations that inhibit by 50% cell growth compared to untreated control (EC_50_ values) and the ratio between the EC_50_ values obtained in the dark and upon light irradiation, also called the photo index (PI), are reported in Table 3.

**Table 3:** (Photo)­cytotoxicity (EC_50_ with 95% Confidence Interval)^a^ and Cellular Uptake (CU with Mean Deviation)^b^ of [**1**]^2+^–[**4**]^2+^ in Lung Cancer Cells (A549) under Normoxic Conditions (21% O_2_)

	[**1**](PF_6_)_2_		[**2**]Cl_2_		[**3**](PF_6_)_2_		[**4**](PF_6_)_2_	
R	H^d^		CCH		H^d^		CCH	
dark	79.7	+6.1	66.0	+12.4	62.1	+16.4	29.4	+2.7
		–5.7		–9.9		–13.8		–2.4
light	20.6	+3.0	5.3	+1.4	13.8	+4.3	7.0	+1.5
		–2.6		–1.1		–3.6		–1.3
PI^c^	3.9		12.5		4.5		4.2	
CU^b^	0.32 ± 0.14		0.73 ± 0.12		0.69 ± 0.16		1.19 ± 0.20	

a The (photo)­cytotoxicity experiments were performed in biological and technical triplicates; all EC_50_ values and 95% confidence intervals are given in μM.

b Cellular uptake (and mean deviation) upon incubation for 24 h in the dark (30 μM). Results are given in nmol Ru/mg cell protein and averaged over three independent experiments.

c The photo index (PI) is defined as EC_50,dark_/EC_50,light_ and has no unit.

d All data for the unfunctionalized complexes (R = H) are taken from ref 44.

In the dark, the cytotoxicity of [**2**]^2+^ was comparable to that of its alkyne-free analogue [**1**]^2+^ (EC_50_ of 66 vs 79 μM), but [**4**]^2+^ was twice as toxic as [**3**]^2+^ (EC_50_ of 29 vs 62 μM). After light activation, both complexes showed increased cytotoxicity compared with dark conditions with similar EC_50_ values (5 and 7 μM for [**2**]^2+^ and [**4**]^2+^, respectively). These values were both lower than those of their nonfunctionalized analogues [**1**]^2+^ and [**3**]^2+^. Interestingly, while the PI for both *i*-Hdiqa-based complexes was above 4, alkyne functionalization of the *i*-biq complex led to an increase in the PI from 3.9 to 12.5. Thus, the effect of the alkyne group on the EC_50_ values was different for the two complexes. In summary, alkyne functionalization in [**2**]^2+^ and [**4**]^2+^ led to an increased cytotoxicity compared to their nonfunctionalized analogues [**1**]^2+^ and [**3**]^2+^ in the dark and after light activation.

To explore the reasons for such changes, we investigated the effect of alkyne functionalization on the cellular uptake of the complexes into A549 cancer cells. The concentration of ruthenium in nmol per mg cell protein was determined by high-resolution continuum-source atomic absorption spectrometry (HRCS AAS) after incubation of the cells for 24 h with 30 μM drug in the dark (Table 3). The results revealed that the alkyne-functionalized complexes [**2**]^2+^ and [**4**]^2+^ were taken up twice as much into A549 cells as their nonfunctionalized analogues [**1**]^2+^ and [**3**]^2+^.^45^ This result suggested that, though the free alkyne group consists of only 3 atoms, it increased the lipophilicity of the complexes. For [**4**]^2+^, a doubling of the Ru concentration in the cells correlated well with the halving of the EC_50_ value, compared with [**3**]^2+^, which was found both in the dark and after light activation (PI remained close to 4). Of note, for [**2**]^2+^, these effects also depended on the conditions, as doubling the amount of ruthenium taken up in the cells before irradiation had only a little effect on the dark cytotoxicity, while after light activation, the EC_50_ value of [**2**]^2+^ was a fourth of that of [**1**]^2+^. Overall, it seems that the presence of the CCH group led to higher cytotoxicity and higher cellular uptake of the complexes, which was probably due to a higher lipophilicity (the log P value for [**1**]­(PF_6_)_2_ and [**3**]­(PF_6_)_2_ was already 2.10 ± 0.27 and 0.45 ± 0.10).^45^ For [**2**]^2+^, however, considering the much-improved PI compared with [**1**]^2+^, it cannot be ruled out that such higher hydrophobicity also results in different intracellular localization and/or mode of uptake for the alkyne-functionalized compound compared to the alkyne-free compound. As noted, this hypothesis cannot be tested, as the nonemissive alkyne-free complex [**1**]^2+^ could not be followed in a cell. In fact, the introduction of an alkyne handle may influence transporter binding, but the possibility of lysosomal accumulation through endocytosis is also a hypothesis that would be consistent with the observed cellular localization data (see below).

To shed light on the mode of action of these PACT agents, more insight into their cellular distribution is required. The alkyne-free PACT agents were nonemissive, but the alkyne handle of [**2**]^2+^ and [**4**]^2+^ offered a unique opportunity to label the compounds via click chemistry after cell treatment. CuAAC with azide AlexaFluor 488 was realized in fixed and permeabilized A549 lung cancer cells, 24 h after addition of the complexes [**2**]­Cl_2_ and [**4**]­(PF_6_)_2_, according to a protocol established by DeRose and co-workers and detailed in the Supporting Information.^40^ Confocal microscopy was then applied for imaging the complexes in the cells.

These cell experiments were first performed with Ru prodrug concentrations ranging from the green light EC_50_ values 5 μM to 25 μM. The postclick fluorescence signal was most obvious at 25 μM for both [**2**]^2+^ and [**4**]^2+^ (Figures S33 and S34), thus 25 μM was used in all follow-up cell imaging experiments. As this concentration is toxic to the cells 48 h after light activation, the incubation time after light activation was further reduced from 24 to 1 h before fluorescent imaging (see Figures S35 and S33 for [**4**]­(PF_6_)_2_ after 24 and 1 h, respectively). In such conditions, the cells were stressed but still alive before fixation, which allowed imaging.

The data shown in Figure 4 for [**4**]^2+^ distinguish four different conditions. In conditions A and C, cells were left untreated, while in B and D, cells were treated with 25 μM [**4**]^2+^ for 24 h in the dark and then irradiated with λ = 520 nm for 1 h (drug-to-light interval, DLI = 24 h, 76 J/cm^2^). In C and D, copper­(I) was omitted during CuAAC, and no fluorescence was observed as expected in the absence of the click catalyst. Only in the presence of the ruthenium compound and of the copper catalyst, green emission was observed (Figure 4B), demonstrating that the click reaction between light-activated [**4**]^2+^ attached to its cellular target and the azido-fluorophore had taken place and that the background fluorescence was minimal under such conditions. Interestingly, the green fluorescence appeared not only throughout the nucleus but also in a bright region adjacent to the nucleus in the cytoplasm, suggesting a dual subcellular localization.

**Figure 4:**
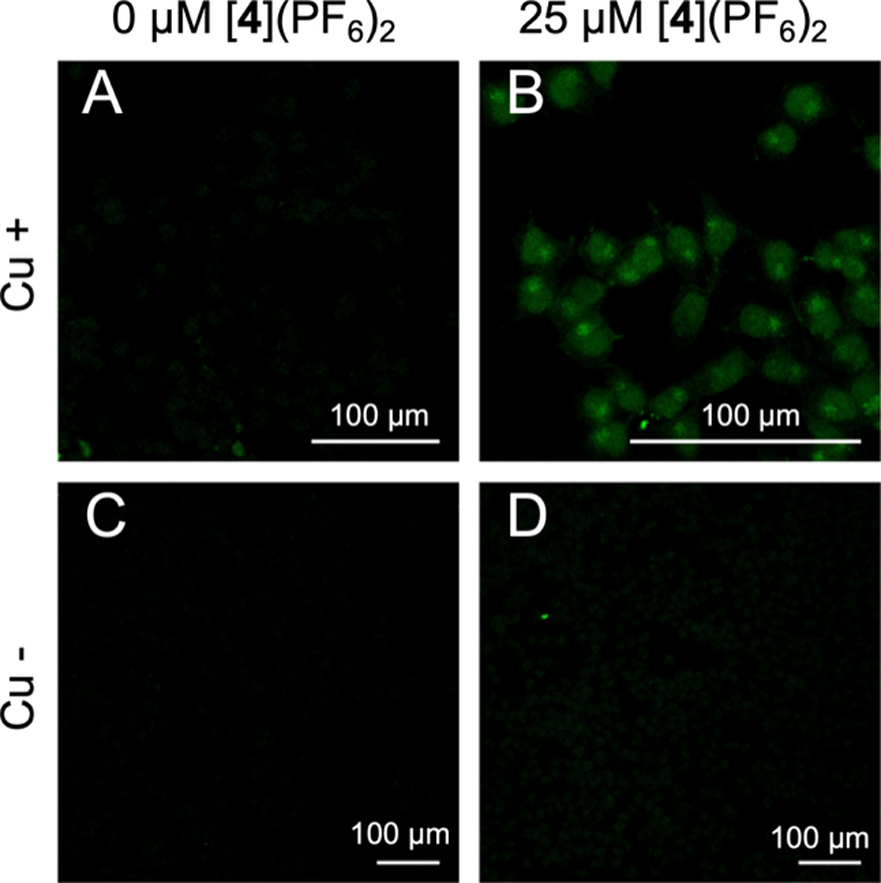
Cellular imaging of alkyne-functionalized PACT agent [**4**]­(PF_6_)_2_ using CuAAC click postfunctionalization with Alexa Fluor 488 fluorophore in fixed cells. A549 cells were either not treated (A, C) or treated (B, D) with 25 μM [**4**]­(PF_6_)_2_ for 24 h and irradiated with λ = 520 nm for 1 h (76 J/cm^2^). In C and D, copper was omitted (see details in the ESI). Scale bar: 100 μm.

This experiment was also realized with [**2**]^2+^, which revealed similarities but also differences with [**4**]^2+^. First, the localization of the fluorescence signals for [**2**]^2+^ and [**4**]^2+^ after light activation were found to be identical (see Figures S39 and S36, respectively), but the fluorescence signal intensity of [**2**]^2+^ was weaker, which might be attributed to the lower uptake of [**2**]^2+^ compared to [**4**]^2+^ (Table 3). These new results clearly demonstrated that the alkyne handle on the ruthenium complexes enabled the labeling of [**2**]^2+^ and [**4**]^2+^ using an Alexa Fluor 488 azide within fixed cells, paving the way for identifying the intracellular targets and for imaging the fate of these otherwise invisible PACT agents.

Without light, the Ru complexes are not activated, and the general assumption in PACT is that they should, in principle, not covalently interact with their targets or interact via weak interactions. This assumption was confirmed here for the first time by the low fluorescence signal observed in dark conditions, shown in Figure 5 (‘Dark’ images). Such a lower intensity signal is likely due to washing off the fluorophore-labeled prodrug from the permeabilized cells, a procedure needed during labeling before microscopy.

**Figure 5:**
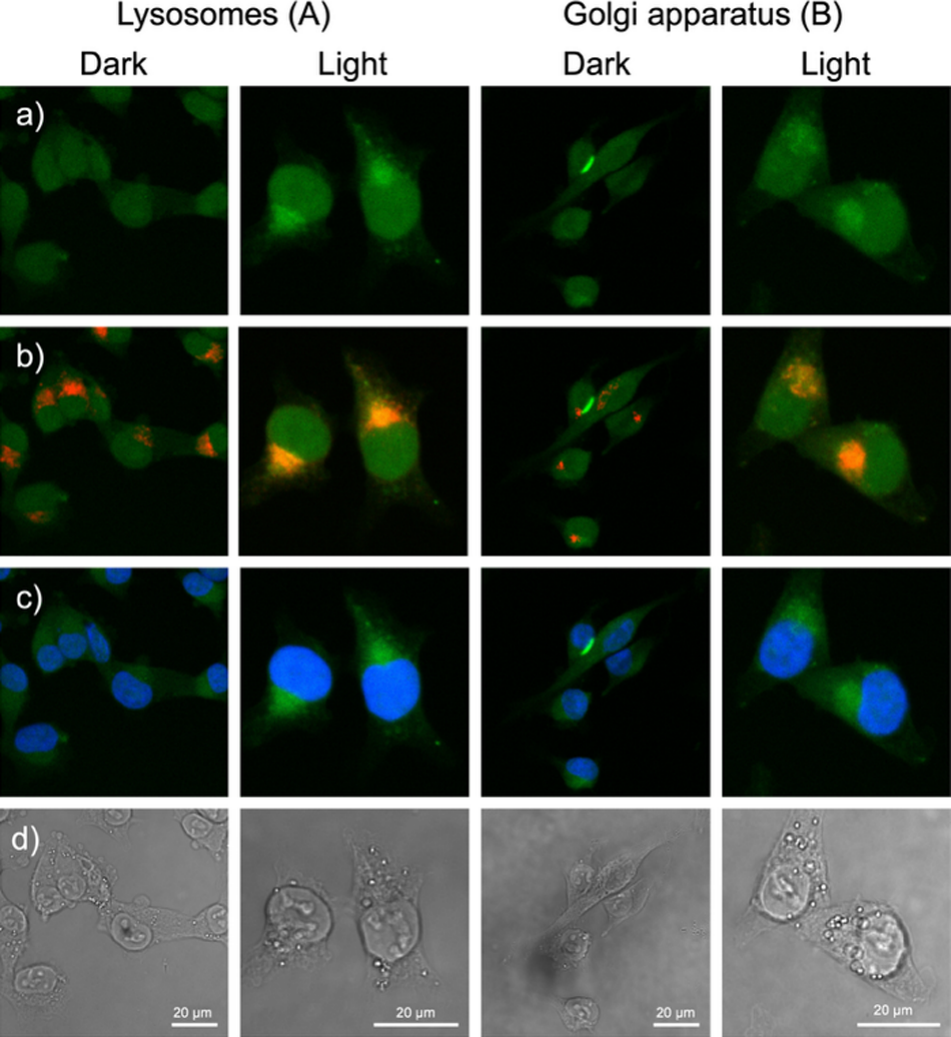
Costaining of lysosomes (left) and Golgi apparatus (right) of A549 cells treated with [**4**]­(PF_6_)_2_ (25 μM) for 24 h (DLI) and activated by green light (right) for 30 min (λ = 520 nm, 38 J/cm^2^) or kept in the dark (left) and further incubated for 1 h. From top to bottom, three fluorescence channels are shown: (a) λ_exc._ = 488 nm, λ_em._ = 520–590 nm, (Cu-click + [**4**]­(PF_6_)_2_), (b) λ_exc._ = 638 nm and λ_em._ = 670–720 nm (organelle), and (c) λ_exc._ = 405 nm and λ_em._ = 424–473 nm (Hoechst). (d) Bright-field images of the cells. In (b), the overlap between green fluorescence from the clicked Ru compound and the red fluorescence of the organelle results in an orange color. Microscopy images taken with a 40× water objective.

Following up on these encouraging results, we further investigated the intracellular localization of [**2**]^2+^ and [**4**]^2+^ by costaining the different cell compartments in the cytoplasm using standard indirect immunofluorescence protocols.^52^ As hydrophobic organelles such as mitochondria, endoplasmic reticulum (ER), lysosomes, and Golgi apparatus are the most common intracellular localizations, we used dedicated primary and secondary antibodies (ABs) to look for the localization of these two PACT agents. The cells were first treated with the ruthenium complex for 24 h and activated with 0, 30, or 60 min of green light as indicated above. Then their organelles were labeled with the corresponding antibodies as indicated in the Supporting Information. To reduce nonspecific antibody binding, bovine serum albumin (BSA) was employed as a blocking agent, enhancing the specificity of antibody–antigen interactions. After thorough washing to remove unbound primary antibodies, a fluorophore-conjugated secondary antibody was introduced to enable fluorescence imaging. The nucleus was stained by using Hoechst dye, which binds to DNA, to facilitate the identification of nuclear structures. The cells were finally labeled with CuAAC and imaged using confocal microscopy across different emission channels. For each organelle, we studied cells kept in the dark (Figure 5, left) and cells irradiated with green light for 30 or 60 min (Figure 5, right). The fluorescence of [**2**]^2+^or [**4**]^2+^ postlabeled with AlexaFluor 488 azide was measured in the green channel a) and the specific organelle costaining in the red channel b). In c), Hoechst is shown as a nucleus costaining in the form of an overlap between the green and blue emission channels.

As shown in Figure 5a, cells treated with [**4**]^2+^, activated with green light, and labeled through CuAAC resulted in green fluorescence throughout the nucleus and a bright cytoplasmic area adjacent to the nucleus. For the mitochondria and the ER, we were unable to see any specific overlap between the emission channel of the secondary antibody and that of the AlexaFluor 488 azide (Figure S36). For the lysosomes and Golgi, the situation was different. In the absence of light activation, red emission was observed from the organelle only, together with a faint green background for the nuclei (Figure 5b, dark). In the presence of light, however, these organelles showed an orange color characteristic of colocalized red and green fluorescence from [**4**]^2+^ (Figure 5b, light). Additionally, more intense green fluorescence was found in the nucleus (Figure 5a,c).

Overall, it seemed that with [**4**]^2+^ both higher cell uptake and covalent interaction with biomolecules contributed to a higher fluorescence intensity. The more intense fluorescence intensity of AlexaFluor 488 after light activation of the ruthenium prodrug suggested indeed that the light-activated complex was not washed out during the labeling procedure and hence that it had covalently attached to biomolecules inside cells. The nonactivated prodrugs were washed out, which was a sign of weak interactions with biomolecules. Additionally, it can be concluded from these costaining experiments that the two alkyne-functionalized PACT agents primarily accumulated in the lysosomes, the Golgi apparatus, and in the nucleus after light activation. As noted, to costain the lysosomes, we used antibodies targeted toward the lysosomal-associated membrane protein 1 (LAMP1). LAMP1 is predominantly located on lysosomal membranes, as it is essential for cellular digestion and waste removal. Costaining with LAMP1 enables insights into the function and dynamics of lysosomes. For the Golgi apparatus, antibodies targeted to the Golgi matrix protein (GM130) were used, as GM130 is a peripheral membrane protein specifically localized to the cis-Golgi network. It is used as a reliable marker for identifying the structure and organization of the Golgi apparatus.

In order to gain a better and more quantitative understanding of the influence of incubation time on the intracellular localization of drug [**4**]^2+^ following light activation and to demonstrate the value of this analytical method to study the fate of PACT prodrugs, we performed several experiments with different light exposures and incubation times. First, we compared 60 min incubation time after activation with 0 h, where imaging was performed immediately after light activation. Second, keeping the incubation time of 60 min, we compared 60 min vs 30 min light exposure. In this experiment, the quantitative colocalization analysis of the obtained images could not be performed using the classical Pearson correlation coefficient (PCC) calculation.^53^ This issue was due to the widespread fluorescence of the clicked compounds after cellular uptake, which overlapped not only with the lysosome and Golgi but also with the nucleus. This widespread distribution led to high PCCs that can be interpreted as false positives for the considered organelles. To solve this problem, an alternative image analysis strategy using the image analysis software CellProfiler was employed. This approach consisted of segmenting both nuclear and cytoplasmic compartments using Hoechst staining (see the ESI). Next, the specific signals from the different organelles were also used to segment lysosomes, Golgi apparatus, mitochondria, and ER. The AlexaFluor 488 signal from the click-activated PACT compound was then quantified in all the identified compartments, and organelles were defined as “positive” when >50% of the red emission area overlapped with the green emission from the complex. The images used for this analysis can be found in the Supporting Information Figures S36–S41.

Figure 6 shows different quantifications of the time evolution of the fluorescence from the clicked light-activated Ru complex in the nucleus or in different organelles. In Figure 6A, we varied the irradiation time from 30 to 60 min (corresponding to light doses of 38 and 76 J/cm^2^, respectively) but kept the incubation time after light irradiation constant (60 min). When doing so, the ratio between the green emission from the cytoplasm and that from the nucleus significantly increased. Hence, more activated PACT molecules localized outside the nucleus when the irradiation time increased. In Figure 6B,C, we kept the irradiation time constant (60 min) but varied the incubation time after light irradiation from 0 (= no incubation, B) to 60 min (C). The organelle distribution shown in Figure 6C is clearly different from that in Figure 6B: according to these data, the light-activated Ru compound [**4**]^2+^ concentrated in the Golgi after 60 min of incubation, while imaging directly after light activation showed a diffuse localization distributed over almost all organelles. These data are the first-in-kind showing that the intracellular motion of a nonemissive PACT compound can be followed in time using click chemistry and image analysis at different time points.

**Figure 6:**
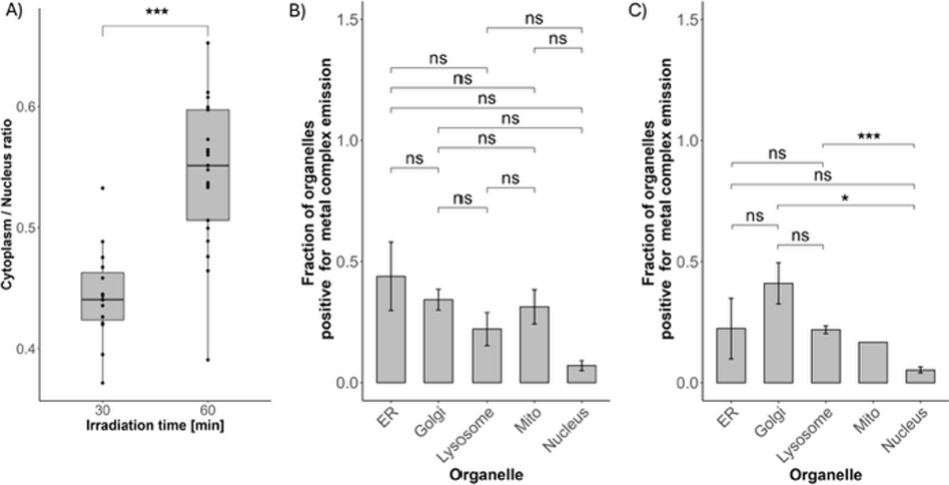
(A) quantified fluorescence ratio between the cytoplasmic and nuclear signals after 60 min of incubation following 30 or 60 min of light activation (*** means *p* < 2 × 10^–6^). (B,C) Localization of the tagged complex through staining of various organelles: the endoplasmic reticulum (*ER*), Golgi apparatus (*Golgi*), mitochondria (*Mito*), and nucleus of the cell at different incubation time points (B: 0 min; C: 60 min) following light irradiation (60 min, 76 J/cm^2^). Error bars represent standard errors.

In fact, the fluorescence observed in the nucleus comes from activated Ru molecules, but these molecules could potentially be bound to many biological binding partners. As DNA is a likely candidate, DNA-binding experiments were performed by using gel electrophoresis. pUC19 DNA was incubated with [**2**]^2+^ or [**4**]^2+^ and irradiated with green light or left in the dark before running the gel. The concentration of pUC19 was kept constant at 1.43 mM, while the concentration of both complexes was increased from 0 to 187.5 μM. The results are shown in Figure S42 and Table S4. For both complexes, the bands moved from ∼2000 to 3000 bp, starting from 18.8 μM of complex concentration. This observation suggested that the pUC19 DNA became progressively unwound upon binding of the complex to DNA. These results suggest that the interaction of these complexes with nuclear DNA is possible.

To complete these purely chemical DNA-binding data in vitro with in cellulo data, a previously unknown and challenging combination with a bio-orthogonal click chemistry labeling method was developed. In this last experiment, labeling of the ruthenium PACT complex [**4**]^2+^ was carried out as described above, but in addition, 5-vinyl-2′-deoxyuridine, the gold standard for metabolic DNA labeling, was added prior to complex activation. In a healthy cell, this artificial 2′-deoxynucleoside is incorporated into nuclear DNA, where it can subsequently be labeled at the vinyl group as a chemical reporter employing a fluorogenic inverse electron-demand Diels–Alder reaction (IEDDA) reaction.^54^ This method allows for observing to what extent DNA replication is enhanced or inhibited. To compare these results with the previous ones, the same drug-to-light interval (60 min) and light activation time (60 min) were used as above, but the ruthenium concentrations had to be reduced from 25 to 5 or 10 μM. Indeed, the incubation time between light activation and imaging had to be increased (to 6, 8, or 24 h) for the cell cycle to have enough time to incorporate the artificial 2′-deoxynucleoside before the cells die. On the other hand, a lower concentration of the PACT molecule renders imaging more challenging. Figures S47 and S48 show the data gathered at both concentrations. At 5 μM, the CuAAC channel images were too faint to conclude, but the data using 10 μM [**4**]^2+^ allowed us to observe emission from both the PACT compound and from the synthesized DNA. The resulting confocal microscopy images are shown in Figure 7. In the absence of PACT treatment, a very strong fluorescence characteristic for the newly formed DNA was visible after 24 h (Figure S49). In the presence of light-activated [**4**]^2+^ (10 μM, Figure S48), the red emission intensity was much lower, showing that almost no replicated DNA was formed after 24 h. Under the influence of 5 μM [**4**]^2+^, the red fluorescence of the newly replicated DNA was more intense (Figure 7) than that with 10 μM [**4**]^2+^ (Figure S48). Altogether, these observations clearly show the effect of the light-activated PACT agent on DNA biosynthesis, and they suggest a correlation between higher concentrations and increased inhibition of DNA replication. Figure 7 shows the time effect on the DNA replication dynamics since it is found to increase between 6 and 8 h incubation (Figure 7a,b, red (column 2)), while between 8 and 24 h (Figure 7b,c) it decreased. According to these results, we postulate that DNA replication was inhibited by the influence of the light-activated chemotherapeutic agents.

**Figure 7:**
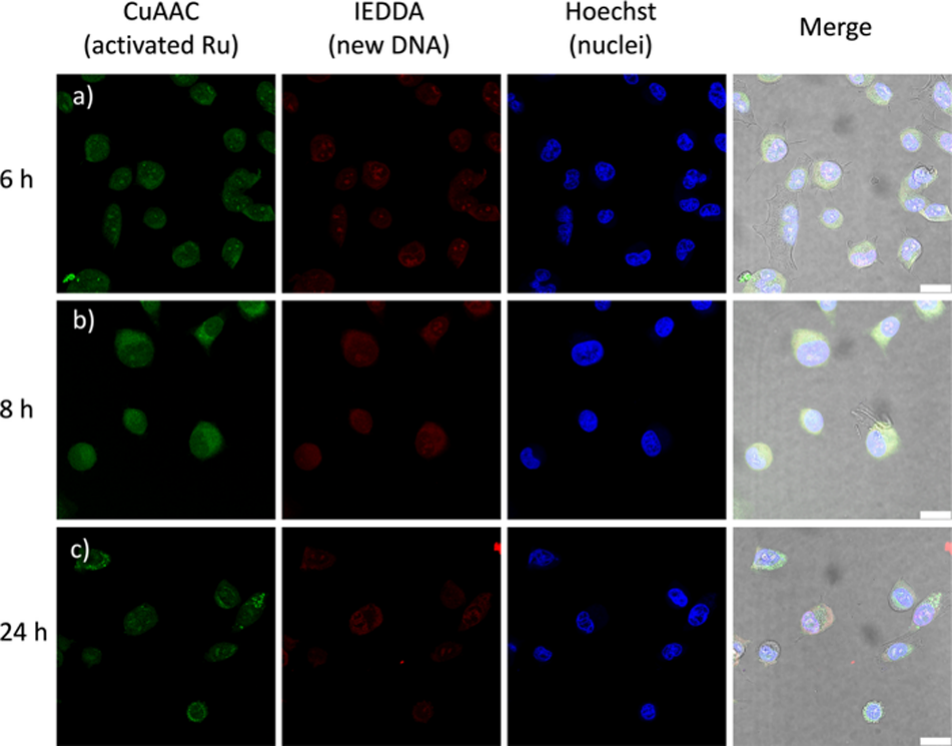
Dual labeling of ruthenium (using CuAAC) and of newly synthesized DNA (using IEDDA) in A549 cells treated with [**4**]­(PF_6_)_2_ (10 μM). Row (a) shows 6 h incubation, (b) 8 h incubation, and (c) 24 h incubation. Column 1 shows the CuAAC channel (Ru, in green), column 2 shows the newly replicated DNA in the IEDDA channel (red), column 3 shows Hoechst (nuclei, blue), and column 4 shows the merge of all 3 channels and of bright-field images of the cells. Scale bar: 20 μm.

## Discussion

Ruthenium-based PACT compounds hold promise to be an effective and mild tool to kill cancerous cells.^54–56^ Albeit the efficiency has been proven in a plethora of studies, their exact localization and thus information about their possible intracellular targets are still elusive. Recent results have suggested the destabilization of the mitochondrial membrane potential^55,56^ or interaction with calcium transporters,^57,58^ but such results cannot be generalized to all compounds. In this study, we pioneer the cellular localization of two nonemissive PACT compounds using bio-orthogonal click chemistry. To achieve this, we functionalized the tpy spectator ligand with a free alkyne group, which represents the smallest possible handle for bio-orthogonal reactions. A small size is a critical factor for the efficient bio-orthogonal labeling using a click handle.^59^ Our data suggest that such functionalization preserves the ground-state structure of the complex and its photochemistry, but that the toxicity and cellular uptake of the alkyne-functionalized compounds were enhanced, compared to alkyne-free analogues.

So far, cellular accumulation and intracellular localization of metallodrugs in general, and PACT compound in particular, was limited to poorly precise surfactant-based organelle-extraction procedures, to analysis using AAS or ICPMS, and to the attachment of a fluorophore.^18^ With the CuAAC-labeling method used in this work, we could visualize for the first time a PACT agent inside cells, and showed that it accumulated preferentially in cancer cells following light activation, and was localized partly in the nucleus and partly in the lysosome and Golgi apparatus. An earlier study supports this hypothesis, as it was shown that [**1**]­(PF_6_)_2_ and [**3**]­(PF_6_)_2_ accumulated less in SH-SY5Y cells when kept in the dark than after light activation.^60^ On the other hand, it is assumed that the nonactivated prodrug cannot engage in coordination interactions with biomolecules and that it is washed out from permeabilized cells because of the protocol used. In this protocol, fixation of the cells is followed by a washing step with Triton X to permeabilize the cell membrane and remove unbound molecules. Unbound, nonactivated complexes may be removed in this step, while our results clearly show that following light activation the PACT molecules must engage in some interaction with biomolecule that withstands such washing.

From our immunostaining experiments with antibodies targeting the four main organelles, we found for both compounds [**2**]^2+^ and [**4**]^2+^ a similar intracellular localization, although the fluorescence signal was less intense for [**2**]^2+^, which is consistent with the lower cellular uptake compared with [**4**]^2+^. The strong fluorescence observed in the nucleus, lysosomes, and Golgi apparatus suggests that both Ru complexes target the three compartments. Although the fluorescence in the nucleus was found to be less intense than in the other two compartments, many Ru compounds are known to bind to DNA,^61–65^ and indeed intercalation studies with pUC19 revealed that light-activated [**2**]^2+^ and [**4**]^2+^ were able to unwind the pUC19 plasmid.^66,67^ They are thus likely to intercalate, overall rendering nuclear DNA as a possible target of these compounds. Under some conditions (Figures S34 and S38), faint labeling of the nucleoli was distinguished from the nuclear labeling (the nucleoli were clearly distinguishable in the bright-field images). This result is not too surprising since both the nucleus and the nucleolus contain high levels of nucleic acids and proteins that are liable to be bound by metal compounds. Very bright nucleolar fluorescence has been reported for click-modified Pt­(II) compounds that are known to aggregate in the nucleolus.^40,68,69^ The stronger nucleolus staining in Figure S38 compared with Figures 5 and S36 is probably a consequence of the twice longer light activation time in Figure S38 (60 vs 30 min), which provides more time for the activated complex to reach the nucleus and interact with the nucleoli. Those experimental findings were extended via a rare study combining two types of click chemistry, i.e., CuAAC and IEDDA. According to this experiment, DNA replication within the nucleus seems to be a significant contributor to cellular stress, with [**2**]^2+^ and [**4**]^2+^. As noted, it is the first time that a method of labeling newly built DNA^70,71^ finds application under the influence of a (photoactivated) chemotherapeutic agent.

Importantly, fluorescence labeling of [**2**]^2+^ and [**4**]^2+^ not only enabled the visualization and localization of the complexes but also allowed tracking of the time evolution of such localization. Upon extending the irradiation time from 30 to 60 min, the complex moved from being more concentrated in the nucleus than in the cytoplasm to a more diffuse localization in the cytoplasm, in particular for [**4**]^2+^. When the incubation time was varied from 0 to 1 h at constant (60 min) green light irradiation time, differences in the distribution of complex [**4**]^2+^ in the different cellular compartments were observed; that is, it localized more in the Golgi apparatus 1 h after light activation. With our limited knowledge on these compounds, such results are difficult to interpret, but the methodology developed allows us to design future experiments where the motion of PACT agents inside cancer cells can be followed in time. As noted, it is impossible at this stage to say by which mechanism the activated ruthenium complex moves inside the cell. A first hypothesis could be that photosubstitution initially produces an aqua complex, as usually hypothesized, and that this aqua complex is inert enough to diffuse inside the cell until it finds a “final” biological target that binds irreversibly to the metal center, thereby stopping the motion. A second hypothesis is that the Ru center binds right after light activation to an initial biomolecule, hence not necessarily forming an aqua complex, and that this initial Ru-biomolecule photoproduct either undergoes further ligand exchange or relocalizes over time until it finds its final target, where irreversible binding takes place. Our results showing motion of the complex inside the cell when the postactivation incubation time increased tend toward the second hypothesis and suggest that 60 min after the end of light activation the final location has not necessarily been reached yet. A full proteomic study at different time points would be necessary to answer what the initial and final targets of the activated complex are and at what time the final target is reached, if a final target exists.

## Conclusions

Two new ruthenium-based PACT agents modified with alkyne groups were synthesized where the photosubstituted ligand is a nontoxic protecting group, and the ruthenium center generates the anticancer effect. The X-ray structure and photosubstitution properties of the complexes remained largely unchanged, compared to non-functionalized analogues, suggesting that the free alkyne moiety is a good candidate for “minimal” functionalization of this class of complexes. The alkyne-functionalized complexes allowed for visualizing the light-dependent activation of these complexes inside the cell as the nonactivated prodrugs had such weak interaction with intracellular biomolecules that they were washed away before imaging. The localization of the light-activated PACT complexes in cancer cells was shown to be possible using post-treatment CuAAC, and we determined using this method that the complexes mainly localize in the nucleus, Golgi apparatus, and lysosomes but move inside the cells to concentrate in the Gogli. Finally, a dual labeling experiment combining CuAAC with IEDDA allowed to demonstrate that light activation of [**4**]^2+^ inhibited DNA synthesis. Overall, DNA-binding experiments, nuclear localization, and DNA replication inhibition suggest that [**4**]^2+^ might target nuclear DNA, while localization in the Golgi and lysosomes suggest that the mode of action of these ruthenium-based PACT agents may not only be DNA-dependent.

Overall, this study represents the first use of CuAAC to trace nonemissive ruthenium-based PACT agents in a cell, suggesting that this method can be used also for studying nonemissive light-activated metallodrugs.^72,73^ In addition, these results provide the first example of dual labeling in cellulo to track DNA replication under the influence of a photoactivated chemotherapy agent. The combination of metabolic labeling using the IEDDA reaction with ruthenium-based chemotherapeutics lays the foundation for the development of new diagnostic methods in cellulo.

## Supplementary Material





## Notes

Synthetic procedures; full chemical characterization of synthesized compounds; X-ray crystallography data; DFT calculations; mass spectrometry, UV–vis spectroscopic data; photochemistry data (extinction coefficients, singlet oxygen quantum yields, photosubstitution quantum yields); cytotoxicity and cellular uptake data; dark stability data; click reaction procedures and microscopy images; imaging details; DNA gel electrophoresis data; image analysis procedure with CellProfiler; dual labeling procedure and data (PDF)
DFT-ru-HCCtpy-idiqa-hmte-water (XYZ)
