# Supplementary material for: Visualizing the Invisible: Dual Click Imaging of Ruthenium-Based Photoactivated Chemotherapy Agents and Their DNA Synthesis Inhibition in Fixed Cancer Cells
Source: J Am Chem Soc. 2025 Nov 5;147(46):42500–10. doi: 10.1021/jacs.5c13249 (PMC12636004; doi:10.1021/jacs.5c13249)
Supplement: Supplementary file 1 [file ja5c13249_si_001.pdf]

Supporting Information

Visualizing the invisible: dual click imaging of ruthenium-based photoactivated chemotherapy agents and their DNA synthesis inhibition in fixed cancer cells

Anja Busemann, [†a] Lisa Rieger, [†b] Rachael M. Cunningham, [c] Sam C. Davidse,[d] Irene Regeni,[a] Ingrid Flaspohler,[a] Claudia Schmidt, [f] Xue-Quan Zhou, [a] Vincent van Rixel,[a] Maxime A. Siegler,[e] Ingo Ott,[f] Sylvia E. Le Dévédec,[d] Hans-Achim Wagenknecht*,[b] Victoria J. DeRose*,[c] Sylvestre Bonnet*[a]

[a] Leiden Institute of Organic Chemistry, Leiden University, Einsteinweg 55, 2333 CC Leiden, Netherlands.

[b] Institute of Organic Chemistry, Karlsruhe Institute of Technology (KIT), Fritz-Haber-Weg 6, 76131 Karlsruhe, Germany.

[c] Department of Chemistry and Biochemistry, University of Oregon, 91 Klamath Hall, Eugene, OR 97403, USA.

[d] Leiden Academic Centre for Drug Research (LACDR), Leiden University, Einsteinweg 55, 2333 CC Leiden, Netherlands.

[e] Small Molecule X-Ray Facility, Department of Chemistry, Johns Hopkins University, Baltimore, Maryland 21218, USA.

[f] Institute of Medicinal and Pharmaceutical Chemistry, Technische Universität Braunschweig, Beethovenstrasse 55, 38106 Braunschweig, Germany

[†] Both authors contributed equally to this work.

*Corresponding author E-mail: bonnet@chem.leidenuniv.nl, derose@uoregon.edu, wagenknecht@kit.edu

1. Table of content

1. Table of content ... 2
2. Synthesis ... 3
2.1. Materials and methods ... 3
2.1.1. Synthesis of [Ru(RCC-tpy)(*i*-biq)(Cl)]Cl (R = TBDMS) [6] ... 3
2.1.2. Synthesis of [Ru(RCC-tpy)(*i*-Hdiqa)(Cl)]Cl (R = TBDMS) [7] ... 5
2.1.3. Synthesis of [Ru(RCC-tpy)(*i*-biq)(Hmte)]$(PF_6)_2$ (R = TBDMS) [8] ... 7
2.1.4. Synthesis of [Ru(RCC-tpy)(*i*-Hdiqa)(Hmte)](PF6)2 (R = TBDMS) [9] ... 9
2.1.5. Synthesis of [Ru(HCC-tpy)(*i*-biq)(Hmte)]$(PF_6)_2$, [2]$(PF_6)_2$ ... 11
2.1.6. Synthesis of [Ru(HCC-tpy)(i-biq)(Hmte)]Cl_2, [2]Cl_2 ... 13
2.1.7. Synthesis of [Ru(HCC-tpy)(*i*-Hdiqa)(Hmte)]$(PF_6)_2$, [4]$(PF_6)_2$ ... 16
3. Single Crystal X-Ray Crystallography ... 18
4. DFT Calculations ... 18
5. Photochemistry ... 22
5.1. Irradiation experiments monitored with MS and UV-vis ... 22
5.2. Singlet Oxygen quantum yield measurement ... 22
5.3. Molar extinction coefficient in water ... 23
5.4. Singlet oxygen production and phosphorescence ... 24
5.5. Green light activation according to UV-vis spectroscopy and mass spectrometry ... 24
6. Cytotoxicity and cellular uptake ... 26
6.1. Materials ... 26
6.2. Cell culturing ... 26
6.3. Phototoxicity essays ... 26
6.4. Dark stability ... 28
6.5. Determination of light dose ... 28
6.6. Dose response curves for A549 cells ... 29
6.7. Cell-irradiation setup ... 29
6.8. Determination of irradiation times ... 29
6.9. Cellular uptake ... 30
7. Click reactions ... 31
7.1. Materials ... 31
7.2. Treatment ... 31
7.3. Click reaction ... 31
8. Imaging ... 31
8.1. Materials ... 31
8.2. Co-staining ... 32
8.3. Microscopy imaging ... 32
8.4. Images with co-staining using 25 µM concentration of the PACT prodrug ... 35
9. DNA photointeraction studies ... 41
10. Image Analysis with CellProfiler ... 42
10.1. Measurement of cytoplasm /nuclei ratio ... 42
10.2. Specific target image analysis ... 45
11. Dual labelling experiments ... 47
12. References ... 49

2. Synthesis

2.1. Materials and methods

All metal complexes were synthesized in dim light and stored in darkness. All reactants and solvents were used without further purification. All ^{1}H NMR, ^{13}C attachedproton-test NMR (13C-APT NMR) were recorded on a Bruker DPX-300 or DMX-400 spectrometers. Chemical shifts were reported in ppm using deuterated solvents as internal standard (Acetone-d$_6$ 1H: δ = 2.04 ppm, 13C: δ = 29.8 and 206.3 ppm; methanol-d$_4$ 1H: δ = 3.35 and 4.78 ppm, 13C: δ = 49.3 ppm). Coupling constants are given in Hertz. The following abbreviations were used to describe the multiplicity of the signals: s (singlet), d (doublet), t (triplet), q (quartet), quin (quintet) or combination thereof. Mass spectra were recorded using an MSQ Plus Spectrometer. Data was analysed using Microsoft Word, Microsoft Excel and Origin.

4'-Bromo-2,2':6',2"-terpyridine was purchased from TCI Europe; $RuCl_3$ and potassium fluoride from Alfa Aesar; 3-bromoisoquinoline from ABCR; isoquinolin-3-amine, tris(dibenzylideneacetone)dipalladium(0), 1,3-bis(diphenylphosphino)propane, 2-(methylthio)ethanol, and *ter*t-butyldimethylsilylethyne from Sigma Aldrich; and potassium *tert*-butoxide from Acros Organics. The ligand *i*-biq was synthesized according to literature[1]; *i*-Hdiqa, [**1**](PF_6)$_2$, and [**3**](PF_6)$_2$ followed a procedure by Busemann *et. al.*[2]; and [Ru(HCC-tpy)(bpy)(Hmte)](PF_6)$_2$ as described previously by the Bonnet group[3] . The following Scheme S1 is the designed synthetic route for the project.

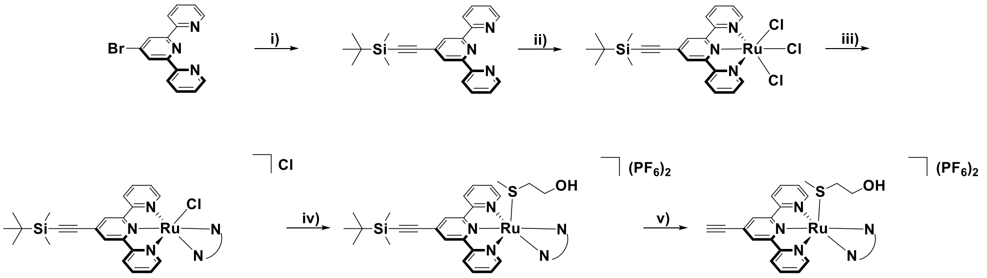

Scheme S1: Reaction scheme of the stepwise synthesis of [**2**](PF_6)$_2$ and [**4**](PF_6)$_2$. Conditions: i) CuI, $Pd(PPh_3)_2Cl_2$, TBDMS-ethyne, Et_3N, 80 °C, N_2, 7 h, 95%; ii) $RuCl_3$, EtOH, 80 °C, 16 h, 75%; iii) LiCl, Et_3N, EtOH/H_2O 3:1), 60 °C, i-biq (overnight, 73%) or i-Hdiqa (5 h, 71%); (iv) Hmte, H_2O, 60 °C, N_2, 16 h, aq. KPF_6; 93 and 95%, respectively; v) KF, MeOH, 30 °C, 16 h, aq. KPF_6; 82 and 83%, respectively.

2.1.1. Synthesis of [Ru(RCC-tpy)(*i*-biq)(Cl)]Cl (R = TBDMS) [6]Cl

[Ru(RCC-tpy)(Cl)$_3$] (251 mg, 0.445 mmol), *i*-biq (114 mg, 0.445 mmol), and lithium chloride (105 mg, 2.50 mmol) were dissolved in degassed ethanol/water mixture (3:1, 40 mL). Triethylamine (160 µL, 1.15 mmol) was added and the reaction mixture was refluxed (60 °C) under dinitrogen atmosphere overnight. The reaction mixture was filtered hot over Celite and the cake was washed with ethanol. After evaporation of the solvents, the crude was purified by column chromatography on silica with dichloromethane/methanol (9:1) as eluent (R_f = 0.70). Yield: 73% (260 mg, 0.325 mmol).

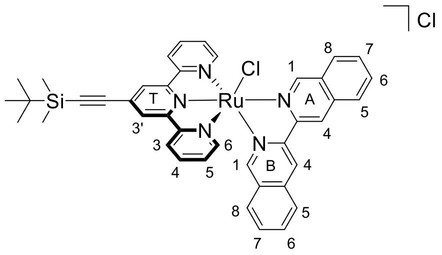

^{1}H NMR (300 MHz, methanol-d$_4$, 298 K) δ (ppm) = 10.78 (s, 1H, A1), 9.34 (s, 1H, A4), 9.03 (s, 1H, B4), 8.74 (s, 2H, T3'), 8.63 (dd, *J* = 8.0, 1.2 Hz, 2H, T3), 8.46 – 8.32 (m, 2H, A5 + A8), 8.11 – 7.94 (m, 4H, B5 + B1 + A6 + A7), 7.94 – 7.82 (m, 4H, T4 + T6), 7.78 – 7.65 (m, 2H, B6 + B8), 7.57 (ddd, *J* = 8.2, 6.8, 1.1 Hz, 1H, B7), 7.29 (ddd, *J* = 7.6, 5.6, 1.3 Hz, 2H, T5), 1.15 (s, 9H, Si-C-(C$\underline{H}_3$)$_3$), 0.34 (s, 6H, Si-(C$\underline{H}_3$)$_2$). *^{13}C NMR (75 MHz, methanol-d$_4$, 298 K)* δ (ppm) = 158.7 + 158.6 (C_q T2 + T2'), 155.5 (A1), 154.7 (A6), 151.6 (T6), 149.5 (C_q A3 or B3), 137.0 (T4), 135.5 +

134.4 (C_q A4a + B4a), 132.4 (B5), 132.1 (B6), 129.9 (A7), 129.4 (B7), 128.9 (C_q A8a or B8a), 127.6 (A5), 127.4 (A8), 127.3 (T5), 127.0 (B1), 126.0 (B8), 124.2 (T3'), 123.9 (T3), 120.1 (A4), 119.5 (B4), 102.6 + 100.2 (C_q C<u>C</u>H + <u>C</u>CH), 25.2 (Si-C-(<u>C</u>H3)3), -6.0 (Si-(<u>C</u>H3)2. *ES MS m/z (calc.):* 764.6 (764.2, $[M-Cl]^+$).

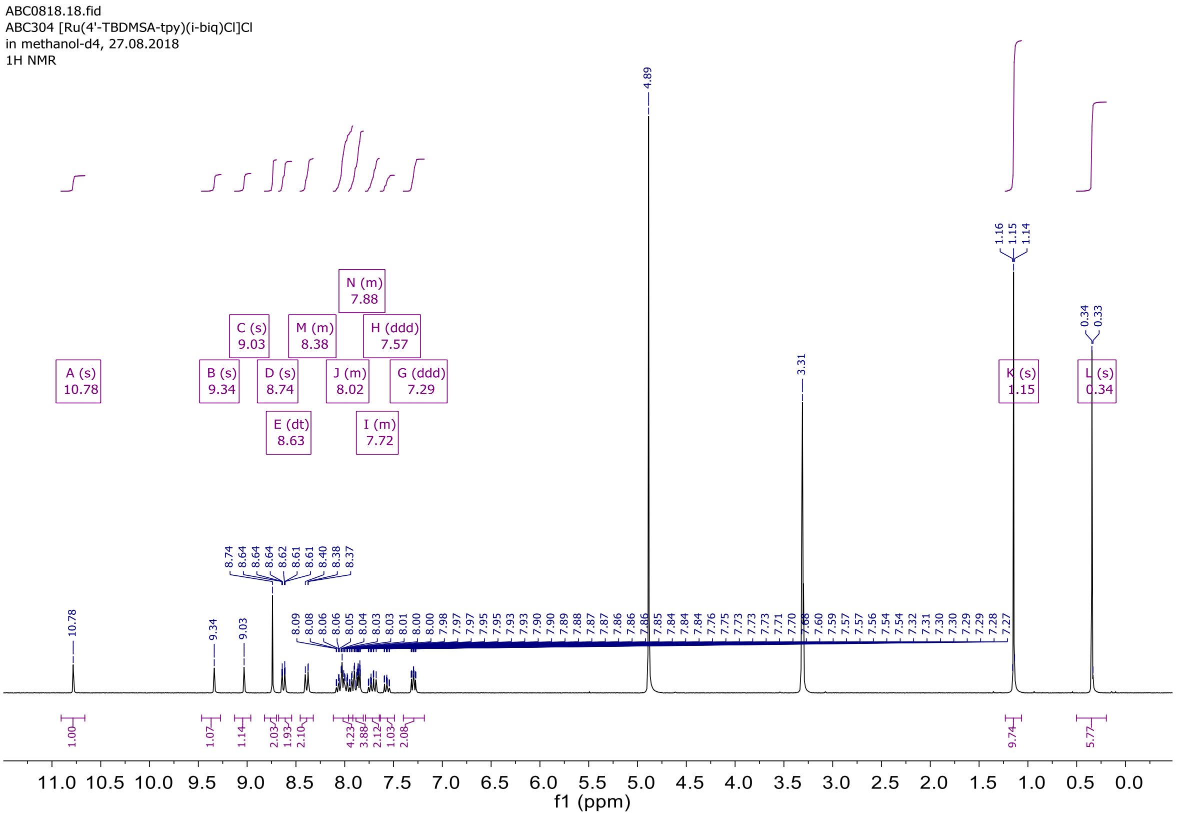

Figure S1: [1]H of compound [**6**]Cl.

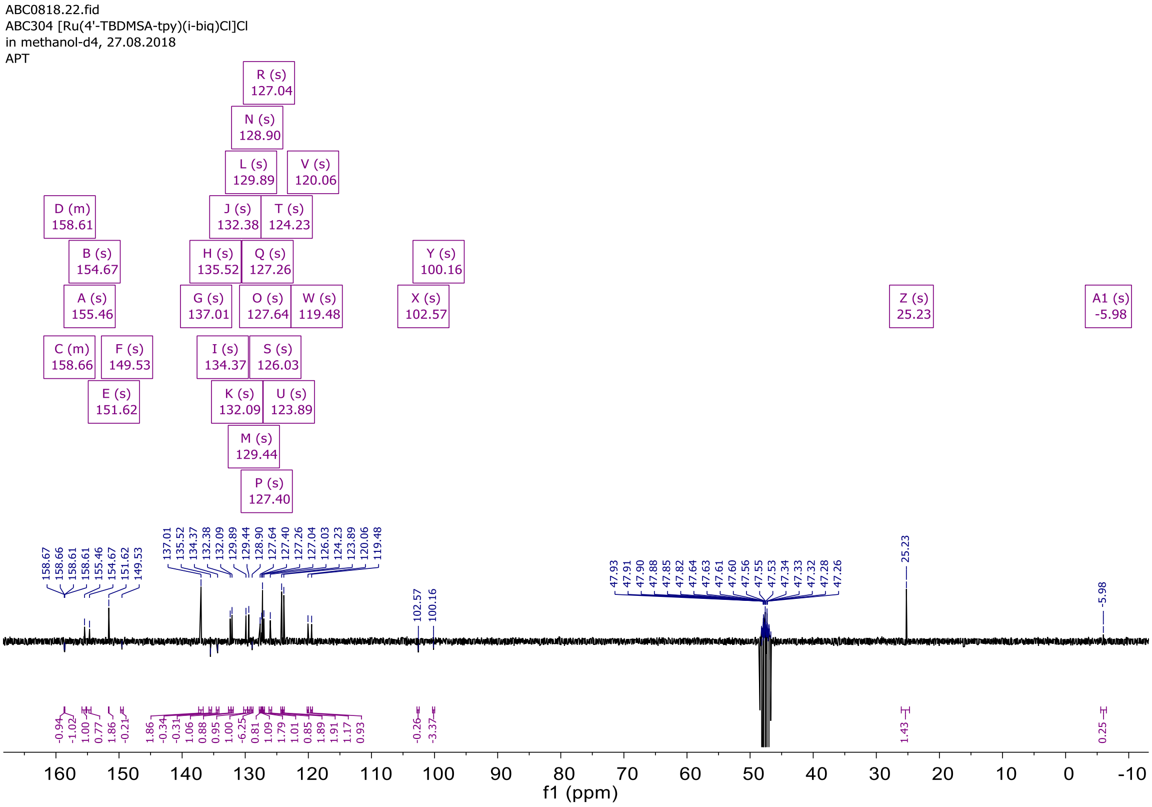

Figure S2: ^{13}C of compound [**6**]Cl.

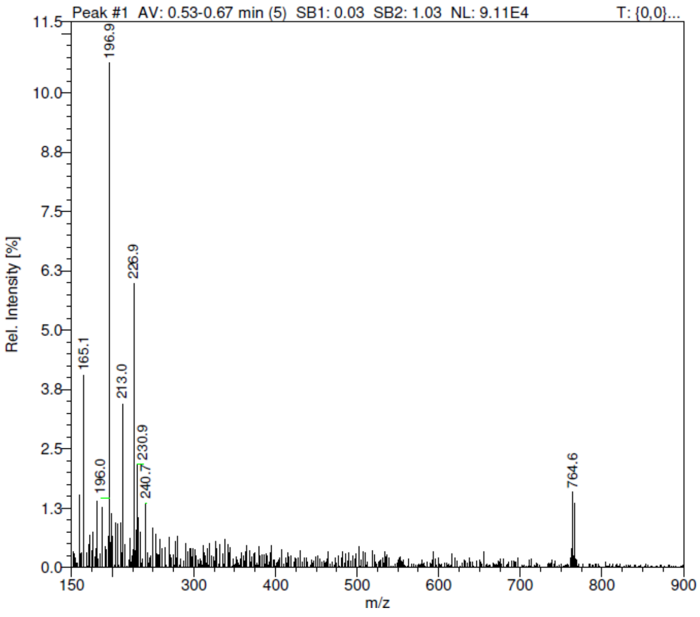

Figure S3: MS of compound [**6**]Cl m/z (calc.): 764.6 (764.2, [M – Cl]$^{+}$)).

2.1.2. Synthesis of [Ru(RCC-tpy)(*i*-Hdiqa)(Cl)]Cl (R = TBDMS) [7]Cl

[Ru(RCC-tpy)(Cl)$_3$] (400 mg, 0.709 mmol), *i*-Hdiqa (192 mg, 0.709 mmol), and lithium chloride (165 mg, 3.94 mmol), were dissolved in degassed ethanol/water mixture (3:1, 64 mL). Triethylamine (252 µL, 1.81 mmol) was added and the reaction mixture was refluxed (60 °C) under dinitrogen atmosphere for 5 h. The reaction mixture was filtered hot over Celite and the cake was washed with ethanol. After

evaporation of the solvents, the crude was purified by column chromatography on silica with dichloromethane/methanol (9:1) as eluent ($R_f = 0.74$). Yield: 71% (413 mg, 0.507 mmol).

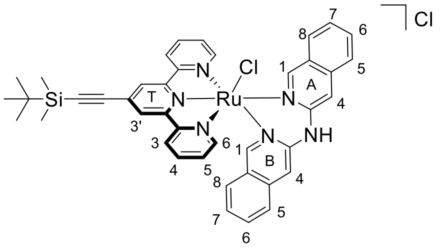

^{1}H NMR (300 MHz, methanol-d_4, 298 K) δ (ppm) = 10.34 (s, 1H, A1), 8.64 (s, 2H, T3'), 8.60 (dd, *J* = 8.0, 1.2 Hz, 2H, T3), 8.58 (ddd, *J* = 5.6, 1.6, 0.7 Hz, 2H, T6), 8.13 (d, *J* = 8.6 Hz, 1H, A8), 8.04 (dd, *J* = 8.5, 1.0 Hz, 1H, A5), 7.99 (td, *J* = 7.8, 1.6 Hz, 2H, T4), 7.88 (s, 1H, A4), 7.85 (ddd, *J* = 8.3, 6.9, 1.2 Hz, 1H, A6), 7.66 – 7.59 (m, 1H, A7), 7.58 – 7.47 (m, 4H, B5 + T5 + B6), 7.45 (s, 1H, B1), 7.39 (d, *J* = 8.3 Hz, 1H, B8), 7.27 (s, 1H, B4), 7.26 – 7.18 (m, 1H, B7), 1.11 (s, 9H, Si-C-(C<u>H</u>$_3$)$_3$), 0.30 (s, 6H, Si-(C<u>H</u>$_3$)$_2$). *^{13}C NMR (75 MHz, methanol-d_4, 298 K)* δ (ppm) = 160.9 + 160.4 (C_q T2 +T2'), 160.0 (A1) 154.5 (T6), 154.3 (B1), 152.3 + 151.3 (C_q A3+ B3), 139.7 + 138.5 (C_q A4a + B4a), 138.5 (T4), 133.7 (A6), 133.5 (B6), 129.1 (C_q T4'), 128.9 (A8), 128.5 (T5), 127.9 + 126.9 (C_q A8a + B8a), 127.7 (A7), 127.5 (B7), 127.4 (B8), 126.8 (A5), 126.1 (B5), 125.6 (T3'), 125.3 (T3), 108.2 (A4), 107.6 (B4), 103.8 + 101.7 (C_q C<u>C</u>H + <u>C</u>CH), 26.6 (Si-C-(<u>C</u>H$_3$)$_3$), 17.6 (C_q Si-<u>C</u>-(CH$_3$)$_3$), -4.6 (Si-(<u>C</u>H$_3$)$_2$). *ES MS m/z (calc.):* 779.5 (779.2, [M – Cl]$^+$).

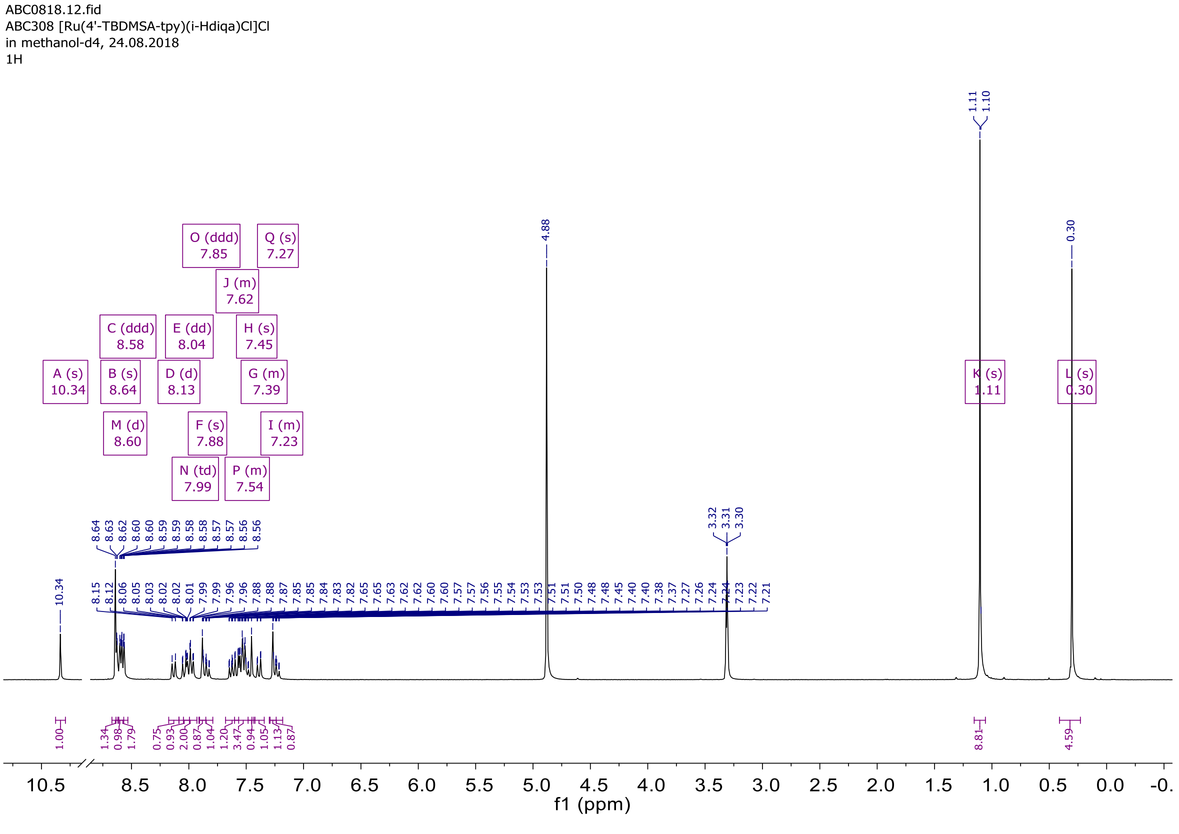

Figure S4: [1]H of compound [**7**]Cl.

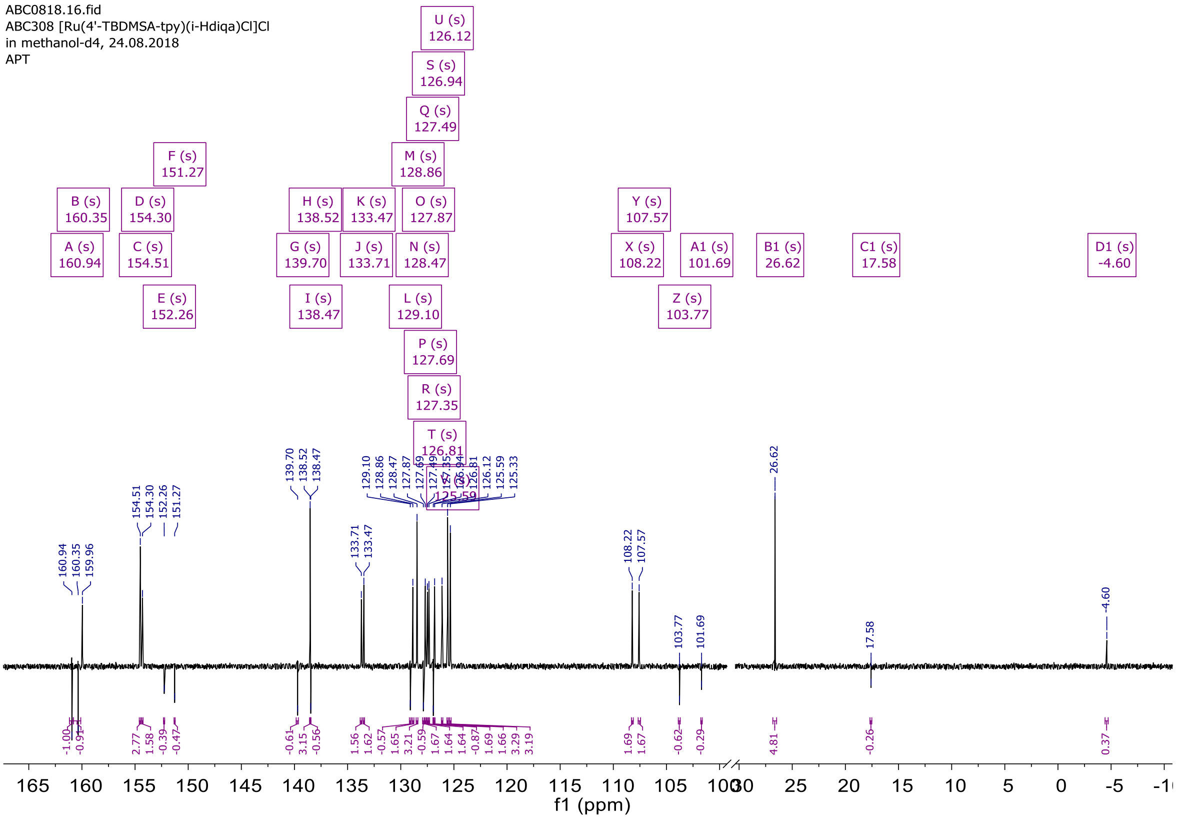

Figure S5: ^{13}C of compound [**7**]Cl.

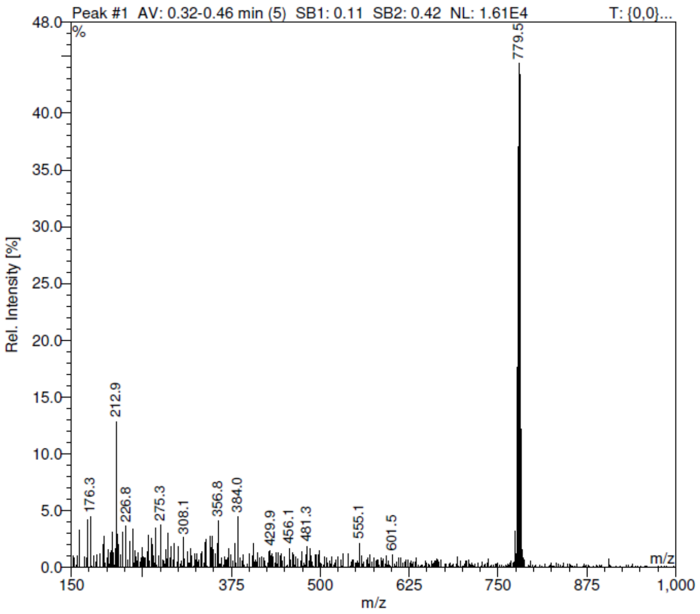

Figure S6: MS of compound [**7**]Cl m/z (calc.): 779.5 (779.2, $[M - Cl]^+$)).

2.1.3. Synthesis of [Ru(RCC-tpy)(*i*-biq)(Hmte)]$(PF_6)_2$ (R = TBDMS) [**8**]$(PF_6)_2$

[Ru(RCC-tpy)(*i*-biq)(Cl)]Cl (151 mg, 0.189 mmol) and 2-(methylthio)ethanol (1 mL, 11 mmol) were dissolved in a degassed water/acetone mixture (4:1, 25 mL). The resultant mixture was stirred and heated to 60 °C under dinitrogen atmosphere overnight. The reaction mixture was filtered hot over Celite and the cake was washed with ethanol. The amount of solvents was reduced by rotary evaporation. The product was precipitated by addition of saturated hexafluoridophosphate, filtered, and washed with cold water. Yield: 93% (195 mg, 0.176 mmol).

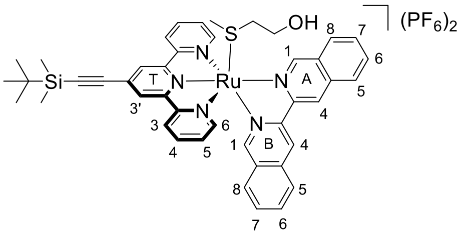

^{1}H NMR (300 MHz, acetone-d$_6$, 298 K) δ (ppm) = 10.64 (s, 1H, A1), 9.52 (s, 1H, A4), 9.28 (s, 1H, B4), 9.01 (s, 2H, T3‘), 8.88 (dt, *J* = 8.0, 1.1 Hz, 2H, T3), 8.49 (dd, *J* = 8.2, 1.2 Hz, 1H, A8), 8.43 (d, *J* = 8.2 Hz, 1H, A5), 8.38 (s, 1H, B1), 8.26 (dd, *J* = 8.0, 1.1 Hz, 2H, T6), 8.20 – 8.09 (m, 4H, T4 + A6 + B5), 8.05 (ddd, *J* = 8.1, 6.9, 1.2 Hz, 1H, A7), 7.84 (ddd, *J* = 8.2, 6.8, 1.3 Hz, 1H, B6), 7.74 (d, *J* = 8.1 Hz, 1H, B8), 7.64 (ddd, *J* = 8.2, 6.8, 1.1 Hz, 1H, B7), 7.50 (ddd, *J* = 7.7, 5.5, 1.3 Hz, 2H, T5), 4.22 (t, *J* = 5.1 Hz, 1H, O<u>H</u>), 3.64 (dt, *J* = 5.6, 5.1 Hz, 2H, S-CH$_2$-C<u>H</u>$_2$), 2.11 (t, *J* = 5.6 Hz, 2H, S-C<u>H</u>$_2$), 1.58 (s, 3H, S-C<u>H</u>$_3$), 1.13 (s, 9H, Si-C-(C<u>H</u>$_3$)$_3$), 0.36 (s, 6H, Si-(C<u>H</u>$_3$)$_2$). *^{13}C NMR (75 MHz, acetone-d$_6$, 298 K)* δ (ppm) = 159.0 + 158.7 (C$_q$ T2 + T2'), 156.6 (A1), 154.8 (B1), 154.3 (T6), 150.7 + 150.2 (C$_q$ A3 + B3), 139.8 (T4), 136.8 + 136.2 (C$_q$ A4a + B4a), 134.2 (A6), 134.1 (B6), 131.4 + 130.7 (C$_q$ A8a + B8a), 131.2 (A7), 130.7 (B7), 129.8 (C$_q$ T4’), 129.7 (T5), 129.0 (A8), 128.7 (B8), 128.4 (A5), 128.3 (B5), 127.1 (T3’), 126.3 (T3), 122.2 (A4), 121.5 (B4), 103.2 + 103.0 (C$_q$ C<u>C</u>H + <u>C</u>CH), 59.0 (S-CH$_2$-<u>C</u>H$_2$), 38.1 (S-<u>C</u>H$_2$), 26.5 (Si-C-(<u>C</u>H$_3$)$_3$), 17.3 (C$_q$ Si-<u>C</u>-(CH$_3$)$_3$), 14.7 (S-<u>C</u>H$_3$), -4.6 (Si-(<u>C</u>H$_3$)$_2$). *ES MS m/z (calc.):* 410.5 (410.6, $[M – 2PF_6]^{2+}$).

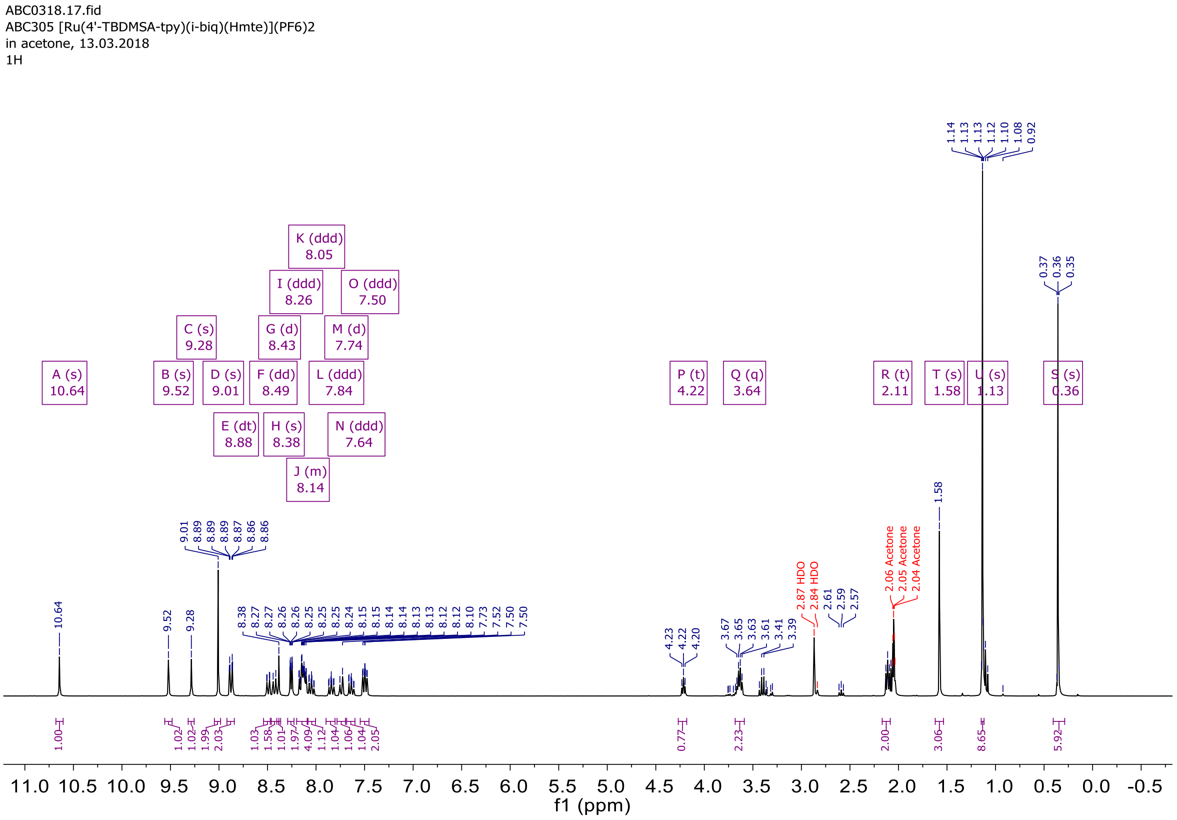

Figure S7: ^{1}H of compound [**8**]$(PF_6)_2$.

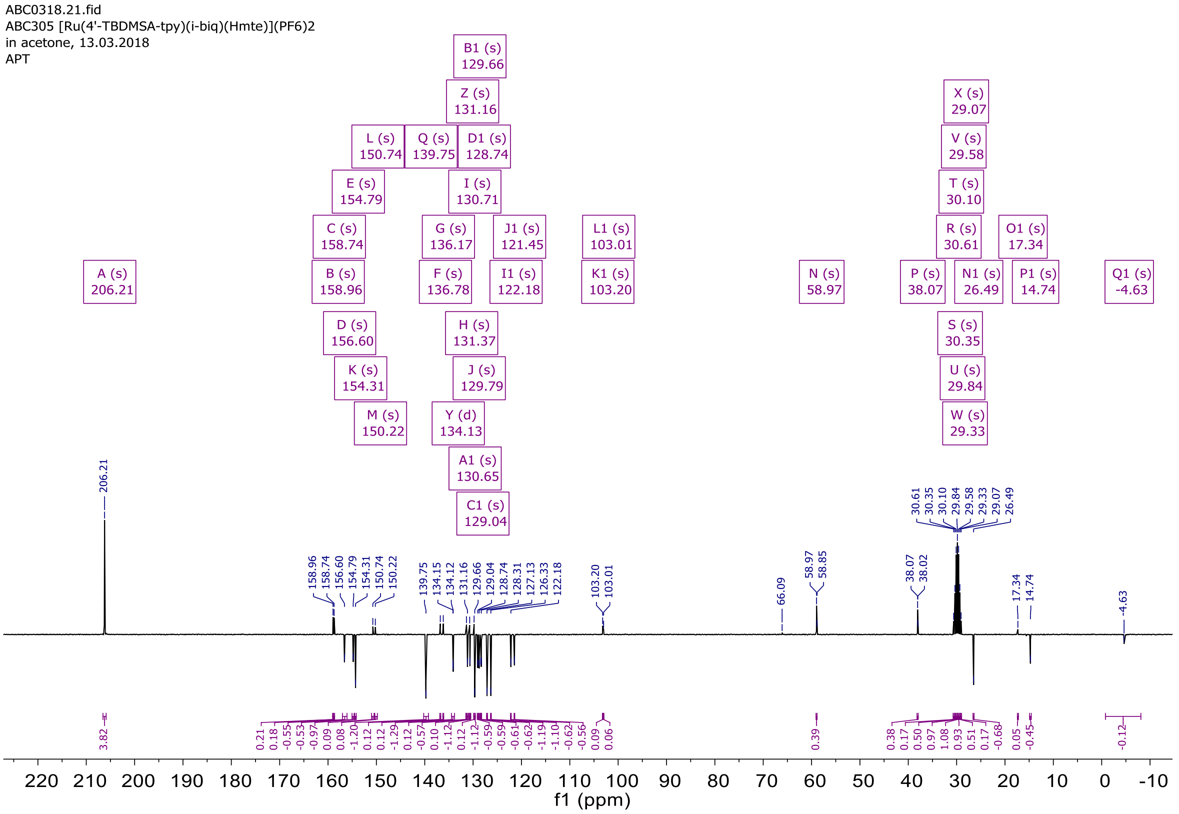

Figure S8: ^{13}C of compound [**8**](PF_6)$_2$.

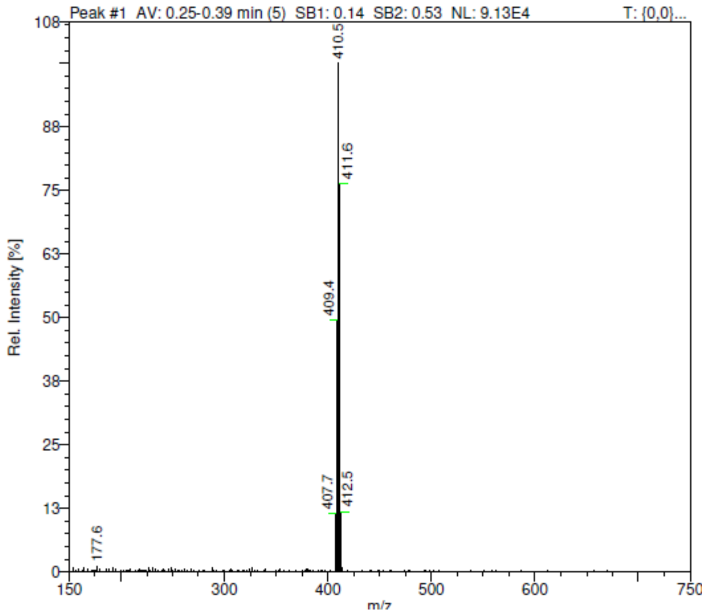

Figure S9: MS of compound [**8**](PF_6)$_2$ m/z (calc.): 410.5 (410.6, $[M - 2PF_6]^{2+}$)).

2.1.4. Synthesis of [Ru(RCC-tpy)(*i*-Hdiqa)(Hmte)](PF_6)$_2$ (R = TBDMS) [9](PF_6)$_2$

[Ru(RCC-tpy)(*i*-Hdiqa)(Cl)]Cl (300 mg, 0.368 mmol) and 2-(methylthio)ethanol (2 mL, 22 mmol) were dissolved in a degassed water/acetone mixture (4:1, 50 mL). The resultant mixture was stirred and heated to 60 °C under dinitrogen atmosphere overnight. The reaction mixture was filtered hot over Celite and the cake was washed with ethanol. The amount of solvents was reduced by rotary evaporation. The product was precipitated by addition of saturated hexafluoridophosphate, filtered, and washed with cold water. Yield: 95% (395 mg, 0.351 mmol).

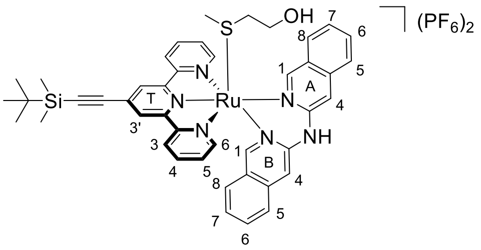

¹H NMR (300 MHz, acetone-d$_6$, 298 K) δ (ppm) = 10.16 (s, 1H, A1), 9.64 (s, 1H, NH), 8.95 (dd, *J* = 5.6, 1.6 Hz, 2H, T6), 8.91 (s, 2H, T3'), 8.87 (dd, *J* = 8.1, 1.4 Hz, 2H, T3), 8.35 (dd, *J* = 8.4, 1.1 Hz, 1H, A8), 8.28 (td, *J* = 7.9, 1.5 Hz, 2H, T4), 8.15 (dd, *J* = 8.5, 1.0 Hz, 1H, A5), 8.10 (s, 1H, A4), 7.95 (ddd, *J* = 8.3, 6.8, 1.2 Hz, 1H, A6), 7.82 (s, 1H, B1), 7.81 – 7.70 (m, 4H, T5 + B5 + A7), 7.63 (ddd, *J* = 8.4, 6.7, 1.2 Hz, 1H, B6), 7.58 (s, 1H, B4), 7.54 (dd, *J* = 8.4, 1.1 Hz, 1H, B8), 7.32 (ddd, *J* = 8.3, 6.7, 1.2 Hz, 1H, B7), 4.06 (t, *J* = 5.1 Hz, 1H, OH), 3.50 (dt, *J* = 5.6, 5.1 Hz, 2H, S-CH$_2$-CH$_2$), 1.92 (t, *J* = 5.6 Hz, 2H, S-CH$_2$), 1.39 (s, 3H, S-CH$_3$), 1.09 (s, 9H, Si-C-(CH$_3$)$_3$), 0.31 (s, 6H, Si-(CH$_3$)$_2$). *¹³C NMR (75 MHz, acetone-d$_6$, 298 K)* δ (ppm) = 159.5 + 159.0 (C$_q$ T2 + T2'), 159.1 (A1), 155.7 (T6), 153.0 (B1), 151.4 + 150.9 (C$_q$ A3 + B3), 139.9 (T4), 139.5 + 138.6 (C$_q$ A4a + B4a), 134.1 (A6), 134.0 (B6), 131.0 (C$_q$ T4'), 129.4 (T5), 129.0 (A8), 128.3 (B8), 128.3 + 126.8 (C$_q$ A8a + B8a) 127.9 (A7), 127.4 (B7), 127.1 (T3'), 126.7 (A4), 126.4 (T3), 126.1 (B5), 110.3 (A4), 109.0 (B4), 103.0 (C$_q$ CCH or CCH), 58.9 (S-CH$_2$-CH$_2$), 37.8 (S-CH$_2$), 26.5 (Si-C-(CH$_3$)$_3$), 17.3 (C$_q$ Si-C-(CH$_3$)$_3$), 15.0 (S-CH$_3$), -4.7 (Si-(CH$_3$)$_2$), one quaternary carbon is missing: C$_q$ CCH or CCH. *ES MS m/z (calc.):* 417.8 (418.1, [M – 2PF$_6$]$^{2+}$).

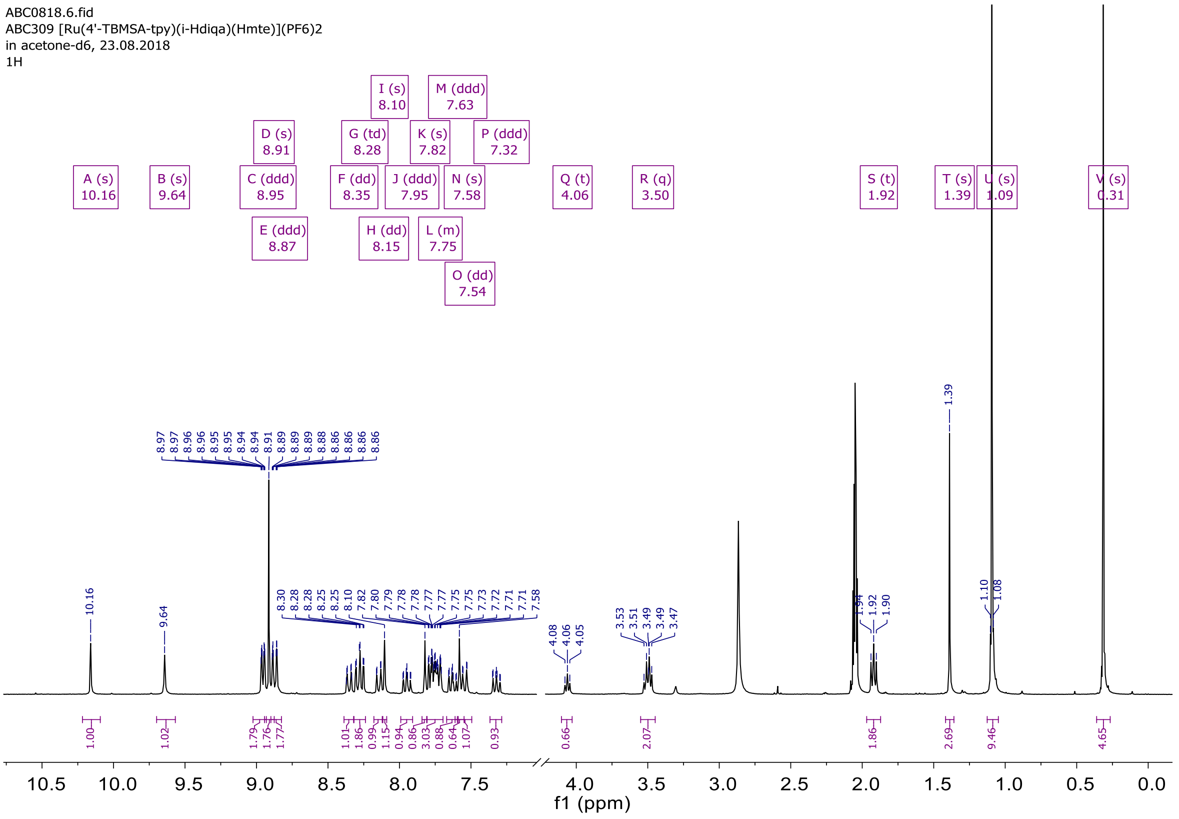

Figure S10: ¹H of compound [**9**](PF$_6$)$_2$.

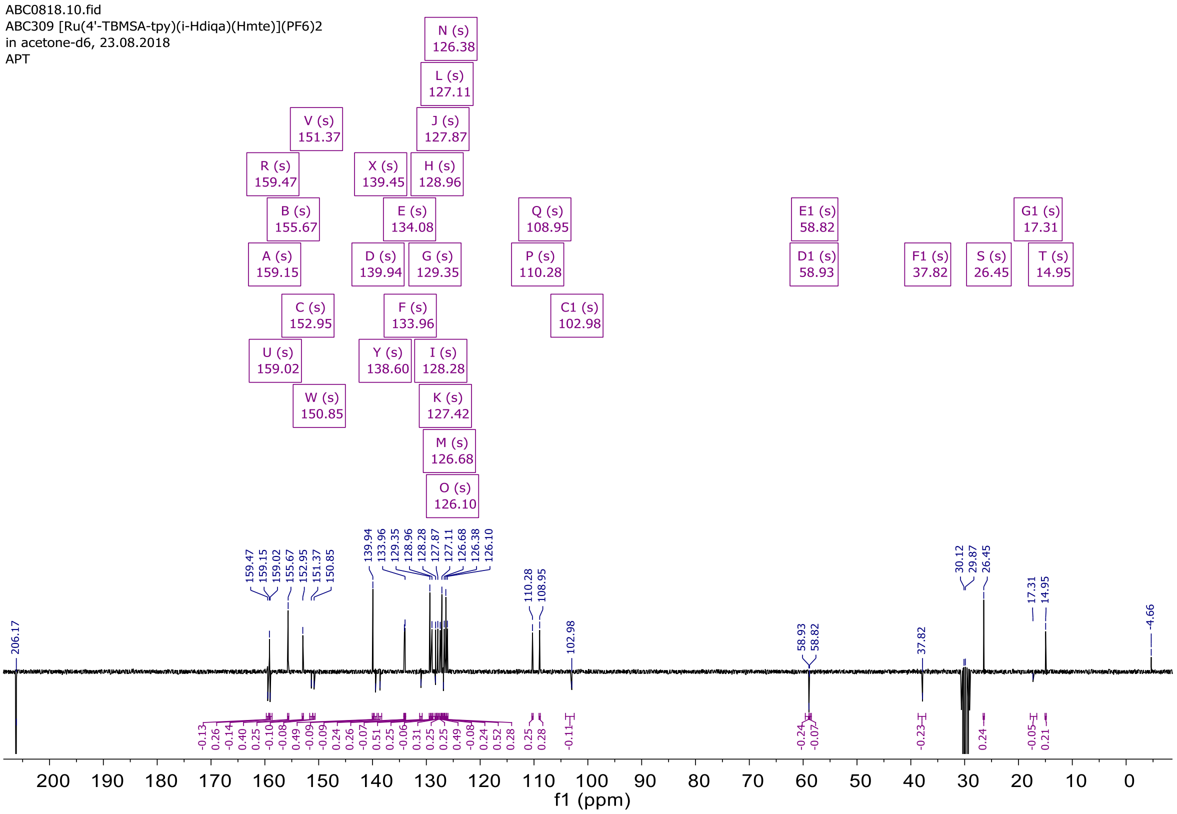

Figure S11: ^{13}C of compound [**9**]$(PF_6)_2$.

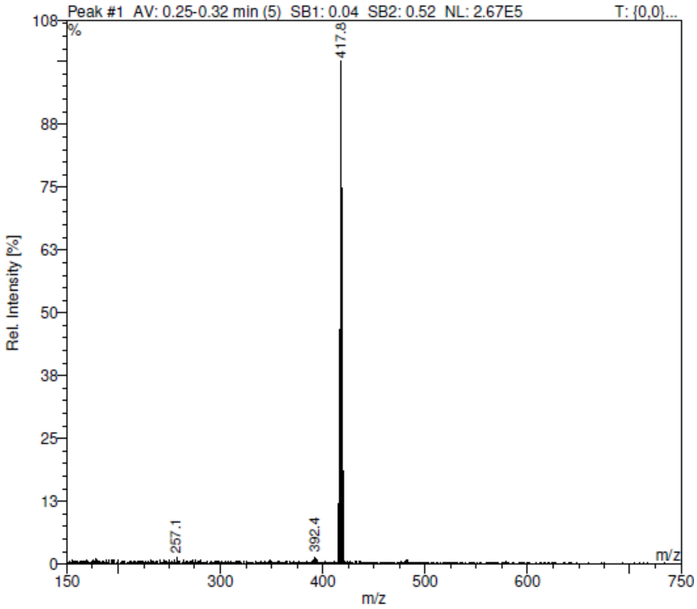

Figure S12: MS of compound [**9**]$(PF_6)_2$ (m/z (calc.): 417.8 (418.1, $[M - 2PF_6]^{2+}$)).

2.1.5. Synthesis of $[Ru(HCC\text{-}tpy)(\textit{i}\text{-}biq)(Hmte)](PF_6)_2$, [2]$(PF_6)_2$

A solution of $[Ru(RCC\text{-}tpy)(\textit{i}\text{-}biq)(Hmte)](PF_6)_2$ (120 mg, 0.108 mmol) in methanol (5 mL) was combined with a solution of potassium fluoride (63 mg, 1.1 mmol) in methanol (5 mL). The resulting reaction mixture was stirred at 30 °C overnight. The amount of solvent was reduced by rotary evaporation and aqueous potassium hexafluoridophosphate was added dropwise to the solution till a precipitate was formed. The precipitate was filtered and washed with cold water. Yield: 82% (88 mg, 0.089 mmol).

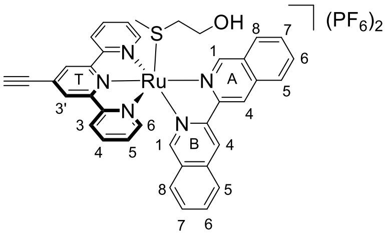

^{1}H NMR (300 MHz, acetone-d_6, 298 K) δ (ppm) = 10.65 (s, 1H, A1), 9.53 (s, 1H, A4), 9.29 (s, 1H, B4), 9.03 (s, 2H, T3'), 8.88 (d, *J* = 8.1 Hz, 2H, T3), 8.50 (d, *J* = 8.1 Hz, 1H, A8), 8.43 (d, *J* = 8.2 Hz, 1H, A5), 8.34 (s, 1H, B1), 8.27 (d, *J* = 5. Hz, 2H, T6), 8.23 – 8.01 (m, 5H, T4 + A6 + B5 + A7), 7.85 (ddd, *J* = 8.2, 6.7, 1.4 Hz, 1H, B6), 7.72 (d, *J* = 8.2 Hz, 1H, B8), 7.64 (ddd, *J* = 8.2, 6.8, 1.1 Hz, 1H, B7), 7.51 (ddd, *J* = 7.7, 5.5, 1.3 Hz, 2H, T5), 4.59 (s, 1H, CC<u>H</u>), 4.26 (t, *J* = 4.7 Hz, 1H, O<u>H</u>), 3.63 (dt, *J* = 5.6, 4.7 Hz, 2H, S-CH_2-C<u>H_2</u>), 2.12 (t, *J* = 5.6 Hz, 2H, S-C<u>H_2</u>), 1.59 (s, 3H, S-C<u>H_3</u>). *^{13}C NMR (75 MHz, acetone-d_6, 298 K)* δ (ppm) = 159.0 + 158.7 (C_q T2 + T2'), 156.6 (A1), 154.8 (B1), 154.4 (T6), 150.8 + 150.2 (C_q A3 + B3), 139.8 (T4), 136.8 + 136.2 (C_q A4a + B4a), 134.2 (A6), 134.2 (B6), 131.2 (A7), 131.0 + 130.7 + 129.8 (C_q A8a + B8a + T4'), 130.7 (B7), 129.7 (T5), 129.1 (A8), 128.7 (A5), 128.4 + 128.3 (B5 + B8), 127.4 (T3'), 126.3 (T3), 122.2 (A4), 121.4 (B4), 87.9 (C<u>C</u>H), 81.3 (C_q <u>C</u>CH), 58.9 (S-CH_2-<u>C</u>H_2), 38.1 (S-<u>C</u>H_2), 14.8 (S-<u>C</u>H_3). *ES MS m/z (calc.):* 354.0 (353.6, $[M - 2PF_6]^{2+}$).

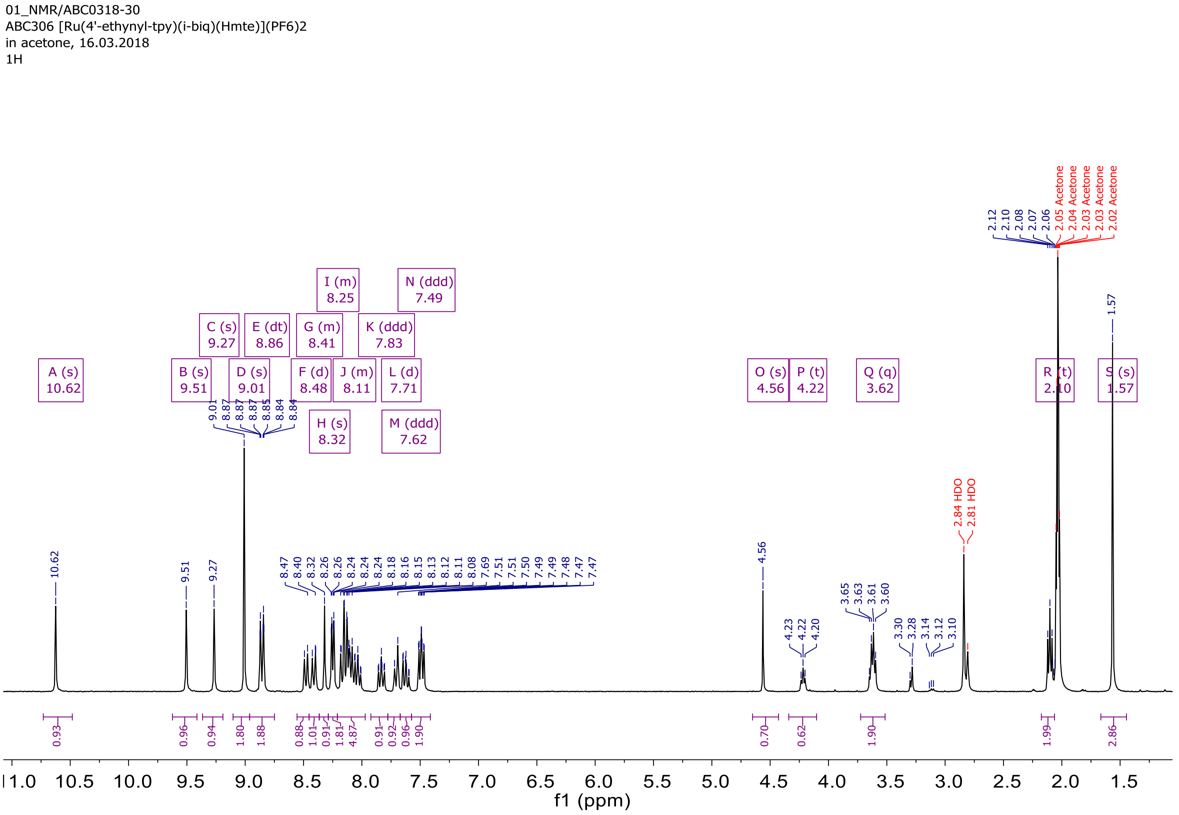

Figure S13: [1]H of compound [**2**]$(PF_6)_2$.

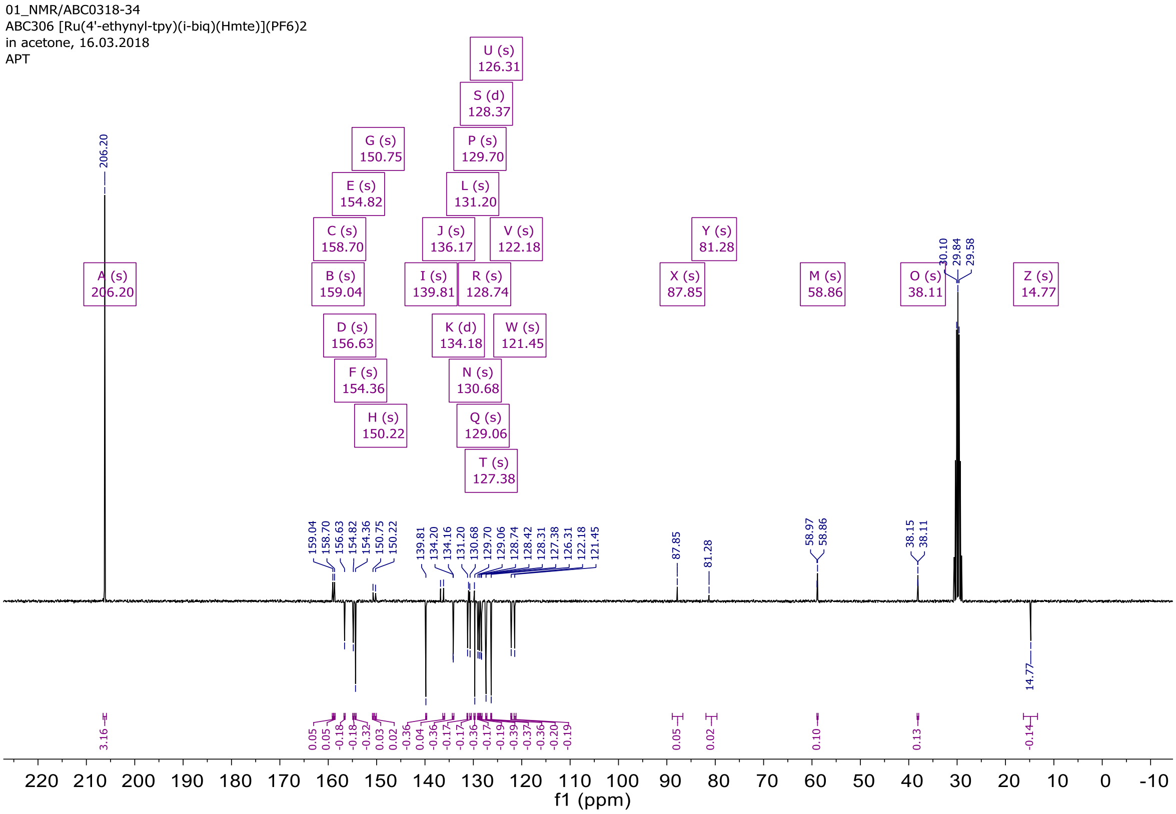

Figure S14: ^{13}C of compound [**2**]$(PF_6)_2$.

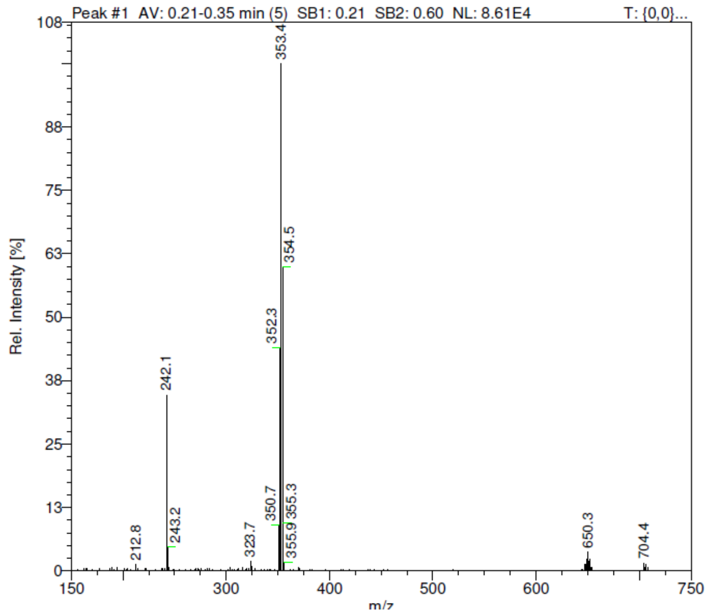

Figure S15: MS of compound [**2**]$(PF_6)_2$ (m/z (calc.): 354.0 (353.6, $[M - 2PF_6]^{2+}$)).

2.1.6. Synthesis of $[Ru(HCC\text{-}tpy)(i\text{-}biq)(Hmte)]Cl_2$, [2]$Cl_2$

[**2**]$(PF_6)_2$ (65 mg, 0.065 mmol) was dissolved in a minimum amount of acetone (1 mL) and saturated Bu_4NCl solution (4 mL) was added dropwise. The formed precipitate was filtered and washed several times with acetone. The product was obtained as brownish red solid. Yield: 99% (50 mg, 0.064 mmol).

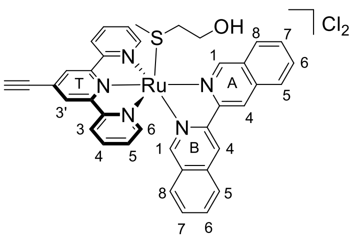

^{1}H NMR (300 MHz, methanol-d_4, 298 K) δ (ppm) = 10.53 (s, 1H, A1), 9.43 (s, 1H, A4), 9.19 (s, 1H, B4), 8.97 (s, 2H, T3'), 8.71 (dt, *J* = 7.9, 1.1 Hz, 2H, T3), 8.49 (d, *J* = 8.1 Hz, 1H, A8), 8.42 (d, *J* = 7.9 Hz, 1H, A5), 8.16 – 7.98 (m, 6H, A6 + B5 + T4 + B1 + A7), 7.94 (dd, *J* = 5.5, 1.5 Hz, 2H, T6), 7.89 – 7.78 (m, 2H, B8 + B6), 7.65 (ddd, *J* = 7.8, 6.5, 1.1 Hz, 1H, B7), 7.42 (ddd, *J* = 7.7, 5.5, 1.3 Hz, 2H, T5), 4.53 (s, 1H, CCH), 3.61 – 3.46 (m, 2H, S-CH_2-CH_2), 1.98 – 1.80 (m, 2H, S-CH_2), 1.42 (s, 3H, S-CH_3). *^{13}C NMR (214 MHz, methanol-d_4, 298 K)* δ (ppm) = 159.3 + 159.1 (C_q T2 + T2'), 156.8 (A1), 154.5 (B1), 154.2 (T6), 151.0 + 150.5 (C_q A3 + B3), 140.2 (T4), 137.3 + 136.7 (C_q A4a + B4a), 134.6 + 134.5 (A6 + B6), 132.1 (C_q A8a), 131.6 (A7), 131.2 (B7), 130.3 (C_q A8b), 130.0 (T5), 129.2 (A8), 129.0 (A5), 128.6 + 128.6 (B8 + B5), 127.8 (T3'), 126.6 (T3), 122.6 (A4), 121.9 (B4), 88.0 (CCH), 81.0 (C_q CCH), 58.6 (S-CH_2-CH_2), 38.4 (S-CH_2), 14.3 (S-CH_3). *ES MS m/z (calc.):* 353.56 (353.70, $[M - 2\,Cl]^{2+}$). *Elem. Anal. Calc. for $C_{38}H_{31}Cl_2N_5ORuS$ + 3 H_2O:* C, 54.87; H, 4.48; N, 8.42. *Found:* C, 54.08; H, 4.08; N, 8.39.

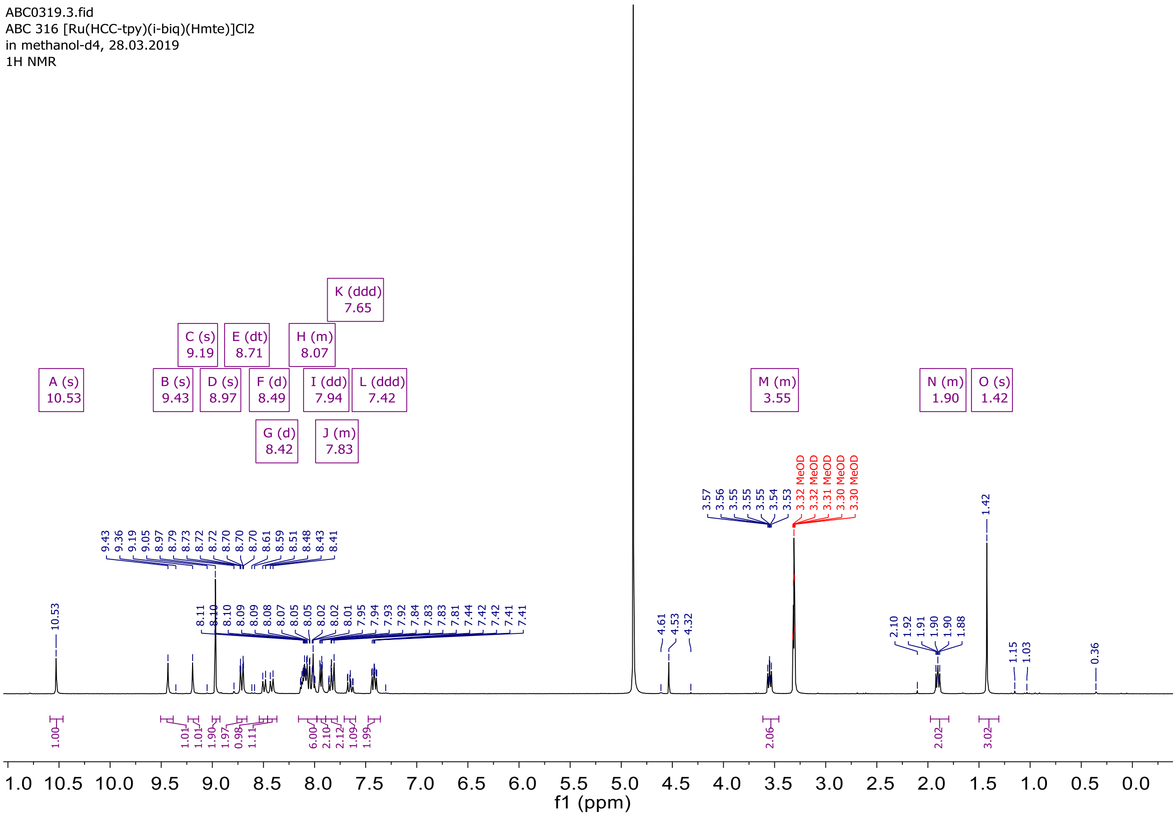

Figure S16: ^{1}H of compound [**2**]Cl$_2$.

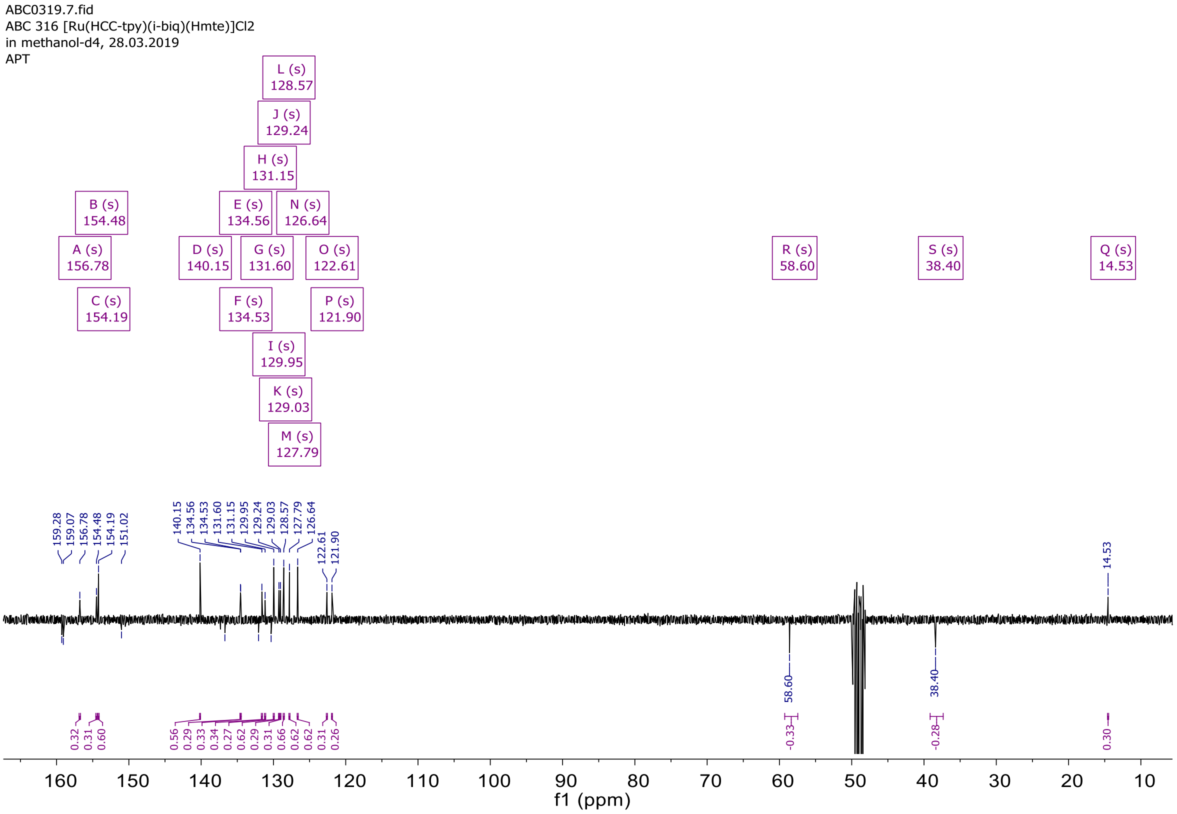

Figure S17: ^{13}C of compound [**2**]Cl$_2$.

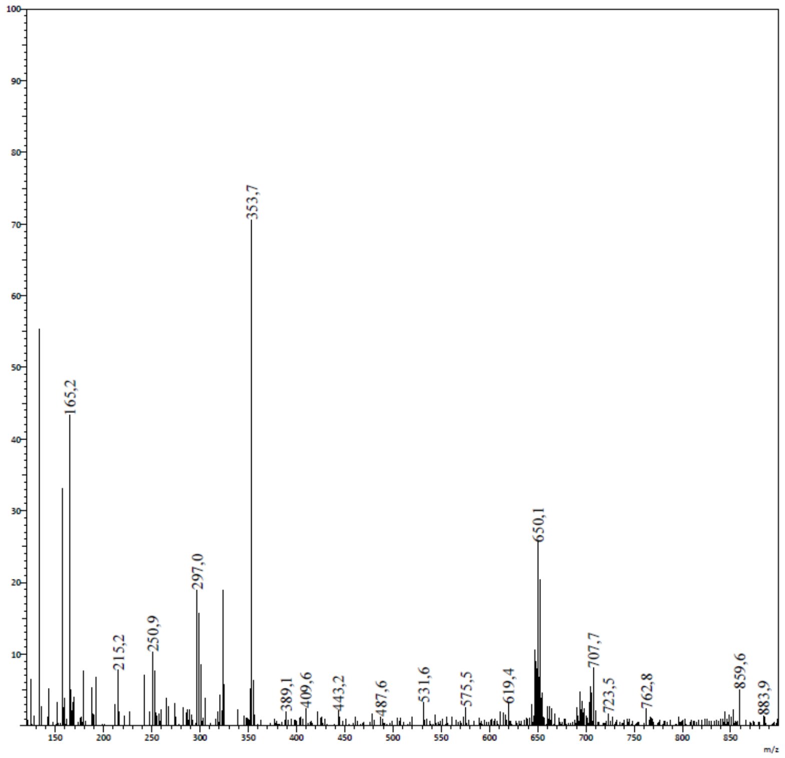

Figure S18: ES MS of compound [**2**]Cl$_2$ m/z (calc.): 353.56 (353.70, [M – 2 Cl]$^{2+}$).

2.1.7. Synthesis of [Ru(HCC-tpy)(*i*-Hdiqa)(Hmte)]$(PF_6)_2$, [4]$(PF_6)_2$

A solution of [Ru(RCC-tpy)(*i*-Hdiqa)(Hmte)]$(PF_6)_2$ (200 mg, 0.178 mmol) in methanol (10 mL) was combined with a solution of potassium fluoride (103 mg, 1.78 mmol) in methanol (5 mL). The resulting reaction mixture was stirred at 30 °C overnight. The amount of solvent was reduced by rotary evaporation and aqueous potassium hexafluorophosphate was added dropwise to the solution till a precipitate was formed. The precipitate was filtered and washed with cold water. The product was obtained as brownish red solid. Yield: 83% (150 mg, 0.148 mmol).

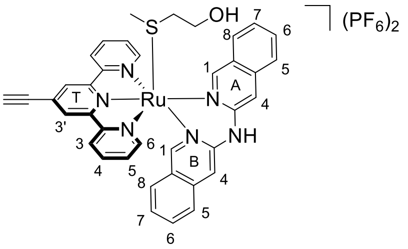

^{1}H NMR (300 MHz, acetone-d_6, 298 K) δ (ppm) = 10.17 (s, 1H, A1), 9.66 (s, 1H, N<u>H</u>), 8.96 (dd, *J* = 5.6, 1.6 Hz, 2H, T6), 8.93 (s, 2H, T3'), 8.87 (dd, *J* = 8.2, 1.4 Hz, 2H, T3), 8.36 (dd, *J* = 8.3, 1.1 Hz, 1H, A8), 8.30 (td, *J* = 7.9, 1.5 Hz, 2H, T4), 8.15 (dd, *J* = 8.6, 1.1 Hz, 1H, A5), 8.11 (s, 1H, A4), 7.95 (ddd, *J* = 8.3, 6.8, 1.2 Hz, 1H, A6), 7.80 (s, 1H, B1), 7.79 – 7.70 (m, 4H, T5 + B5 + A7), 7.63 (ddd, *J* = 8.4, 6.7, 1.2 Hz, 1H, B6), 7.58 (s, 1H, B4), 7.53 (dd, *J* = 8.5, 1.0 Hz, 1H, B8), 7.32 (ddd, *J* = 8.2, 6.7, 1.2 Hz, 1H, B7), 4.52 (s, 1H, CC<u>H</u>), 4.07 (t, *J* = 5.1 Hz, 1H, -O<u>H</u>), 3.50 (dt, *J* = 5.6, 5.1 Hz, 2H, S-CH_2-C<u>H_2</u>), 1.93 (t, *J* = 5.6 Hz, 2H, S-C<u>H_2</u>), 1.39 (s, 3H, S-C<u>H_3</u>). *^{13}C NMR (75 MHz, acetone-d_6, 298 K)* δ (ppm) = 159.6 + 159.0 (C_q T2 + T2'), 159.1 (A1), 155.7 (T6), 152.9 (B1), 151.4 + 150.9 (C_q A3 + B3), 140.0 (T4), 139.5 + 138.6 (C_q A4a + B4a), 134.1 (A6), 134.0 (B6), 130.7 (C_q T4'), 129.4 (T5), 129.0 (A8), 128.3 + 126.8 (C_q A8a + B8a), 128.3 (B8), 127.9 (A7), 127.4 (B7), 127.4 (T3'), 126.7 (A5), 126.4 (T3), 126.1 (B5), 110.3 (A4), 108.9 (B4), 87.9 (C<u>C</u>H), 81.1 (C_q <u>C</u>CH), 59.0 (S-CH_2-<u>C</u>H_2), 37.9 (S-<u>C</u>H_2), 15.0 (S-<u>C</u>H_3). *ES MS m/z (calc.):* 361.0 (361.1, $[M - 2PF_6]^{2+}$). *High resolution ES MS m/z (calc.):* 361.06995 (361.07001, $[M - 2PF_6]^{2+}$). *Elem. Anal. Calc. for $C_{38}H_{32}F_{12}N_6OP_2RuS$:* C, 45.11; H, 3.19; N, 8.31. *Found:* C, 44.54; H, 3.24; N, 8.20.

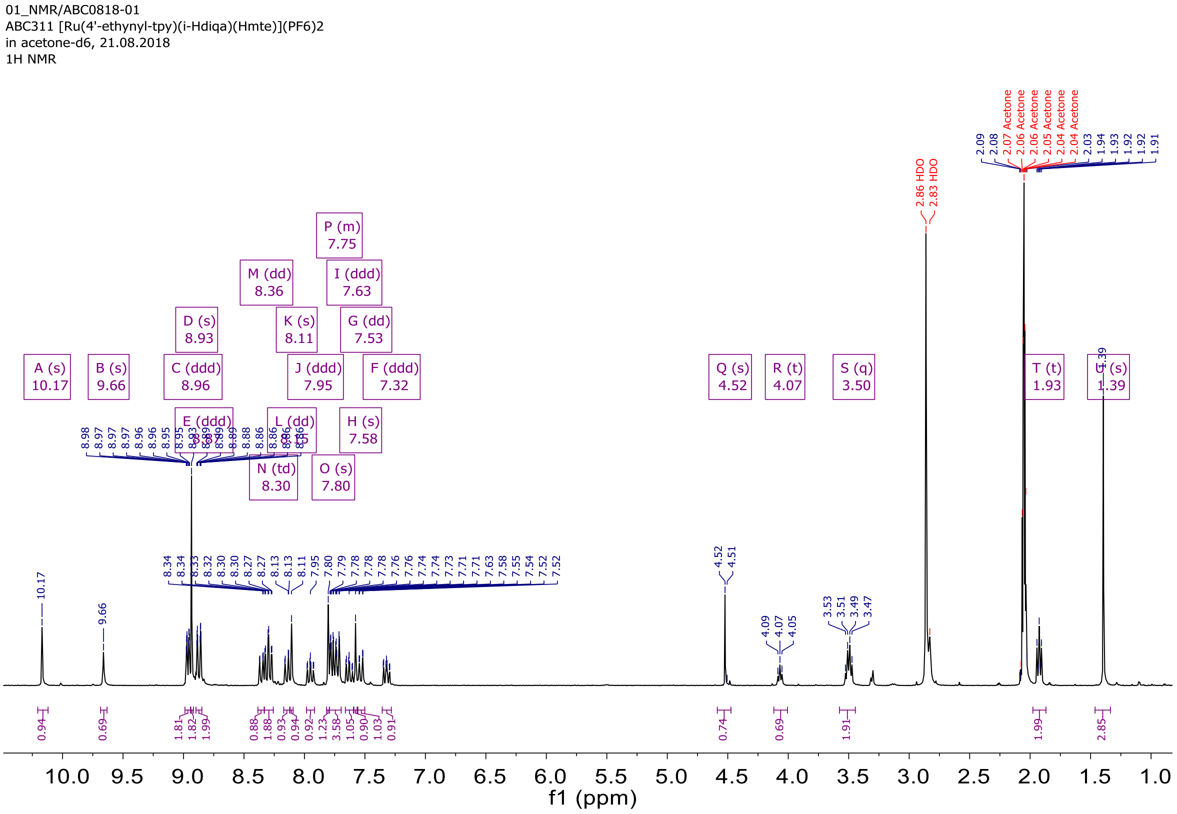

Figure S19: ^{1}H of compound [**4**]$(PF_6)_2$.

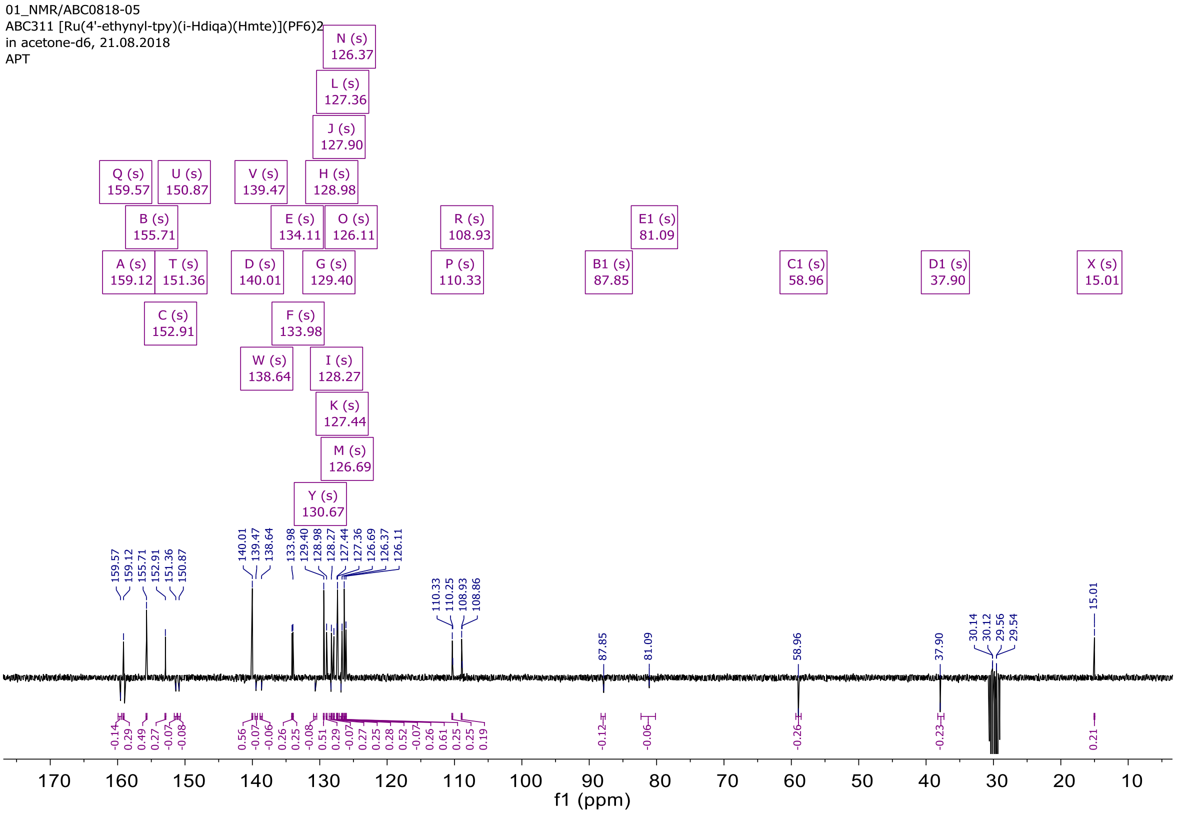

Figure S20: ^{13}C of compound [**4**](PF_6)$_2$.

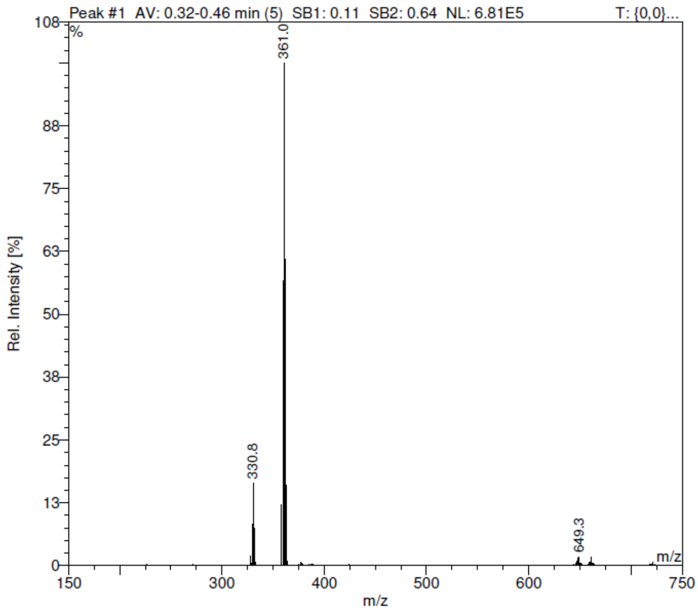

Figure S21: MS of compound [**4**](PF_6)$_2$ (m/z (calc.): 361.06995 (361.07001, [M – 2PF$_6$]$^{2+}$).

3. Single Crystal X-Ray Crystallography

Single crystals of [**2**](PF_6)$_2$ were obtained by recrystallization through liquid-vapor diffusion using cyclopentane as solvent and diethyl ether as counter-solvent. In short, 1 mg of [**2**](PF_6)$_2$ was dissolved in cyclopentane (1 mL) and placed in a small vial. This vial was placed in a larger vial containing diethyl ether (2.8 mL). The large vial was closed and vapor diffusion within a few days afforded X-ray quality crystals.

All reflection intensities were measured at 110(2) K using a SuperNova diffractometer (equipped with Atlas detector) with Cu *K*α radiation (λ = 1.54178 Å) under the program CrysAlisPro (Version CrysAlisPro 1.171.39.29c, Rigaku OD, 2017). The same program was used to refine the cell dimensions and for data reduction. The structure was solved with the program SHELXS-2014/7 (Sheldrick, 2015) and was refined on F^2 with SHELXL-2014/7 (Sheldrick, 2015). Analytical numeric absorption correction using a multifaceted crystal model was applied using CrysAlisPro. The temperature of the data collection was controlled using the system Cryojet (manufactured by Oxford Instruments). The H atoms were placed at calculated positions using the instructions AFIX 23, AFIX 43, AFIX 137, AFIX 147 or AFIX 163 with isotropic displacement parameters having values 1.2 or 1.5 *U*eq of the attached C or O atoms.

The structure of [**2**](PF_6)$_2$ is partly disordered. The 3,3'-biquinoline ligand and one of the two PF_6^- counter ions are found to be disordered over two orientations, and the occupancy factors of the major components of the disorder refine to 0.54(3) and 0.699(17). [**2**](PF_6)$_2$: 0.07 × 0.04 × 0.02 mm³, triclinic, *P*-1, a = 9.6220 (3), b = 11.2316 (4), c = 19.3633 (7) Å, α = 97.533 (3), β = 92.211 (3), γ = 109.604 (3)°, V = 1946.63 (12) Å³, Z = 2, μ = 5.43 mm^{-1}, transmission factor range: 0.779–0.924. 25285 Reflections were measured up to a resolution of (sin θ/λ)max = 0.616 $Å^{-1}$. 7581 Reflections were unique (R_{int} = 0.058), of which 6081 were observed [I > 2σ(I)]. 761 Parameters were refined using 1434 restraints. R1/wR2 [I > 2σ(I)]: 0.0428/ 0.1013. R1/wR2 [all refl.]: 0.0609/ 0.1119. S = 1.02. Residual electron density found between −0.57 and 0.80 e $Å^{-3}$.

Crystallographic data for the structure reported in this Article have been deposited at the Cambridge Crystallographic Data Centre under deposition number CCDC 2476330.

4. DFT Calculations

DFT was used to perform electronic structure calculations. The structure of [**2**]$^{2+}$ and [**4**]$^{2+}$ was optimized using ADF from SCM,[4] the PBE0 hybrid functional, a triple zeta basis set (TZP) for all atoms, and COSMO to simulate solvent effects in water. The nuclear coordinates (Å) of the optimized structure of [**2**]$^{2+}$ and [**4**]$^{2+}$ are given in Table S1 and S2, respectively.

Table S1. Nuclear coordinates (Å) of [**2**]$^{2+}$ minimized at the DFT/PBE0/TZP/COSMO level in water.

Ru	-0.2371536913409365	-0.04131143665661324	0.3460659281257489
C	-1.532890793872343	-2.602328684055745	-0.8306710061644537
H	-2.251842348881358	-1.911567141887859	-1.252225677621425
C	-1.642526304531791	-3.96624060809431	-1.038131573614926
H	-2.459204774094477	-4.350589289423185	-1.634600041543079
C	-0.7013743566126036	-4.806788840837971	-0.4691272946413964
H	-0.7606423864704916	-5.879266502288916	-0.6057782673450046
C	0.3244371977643025	-4.256496998357359	0.280980597153343
H	1.073834674795002	-4.891602003092336	0.7329816069243424
C	0.3796309229394406	-2.882849869395558	0.452020830275026

C	1.433658431222659	-2.209775445273846	1.229689737130766
C	2.508944179645303	-2.816744716864388	1.854185679326281
H	2.638186424226729	-3.889234316979597	1.828005450072141
C	3.442045979451416	-2.018614854871485	2.521557608428701
C	3.283798981528712	-0.6306698787685715	2.528909377656344
H	4.013082308074423	-0.008006548648022216	3.02708294803903
C	2.188715956496207	-0.07747895341463253	1.888875034334967
C	1.878763640275484	1.357303476414452	1.77341761348179
C	2.678870773488598	2.350340032078643	2.314778091310427
H	3.578432050871337	2.089844977980936	2.855303860191404
C	2.31659698085506	3.677117784872823	2.154734344427466
H	2.932303671824132	4.463886659186145	2.572391886780417
C	1.161037402799495	3.976758199036288	1.453955813852466
H	0.8391700867768646	4.99842639341749	1.301868740976054
C	0.4044605322992431	2.939773235551277	0.9370884192813959
H	-0.5053942363915487	3.133427350325226	0.3837876110025992
N	-0.5561908969102859	-2.061993051065329	-0.09824363547775791
N	1.290643964402534	-0.8729698396351607	1.27972252057514
N	0.7411276968266891	1.656692544693616	1.088450397646056
C	-2.904625694685204	1.30973345151026	-0.4209406170955253
H	-3.159074546272399	1.119331693394778	0.6153244934058034
C	-3.829177188865098	1.972253477042693	-1.246029962462154
C	-5.081237029510736	2.409650093749971	-0.7637294254597728
H	-5.34448276509836	2.229980511678045	0.2726116553637966
C	-5.942831019191246	3.053993630841919	-1.609664473666664
H	-6.905897726169232	3.394462803479109	-1.247638822633878
C	-5.587886540448488	3.281488799698712	-2.957145039524841
H	-6.285976707378517	3.793348696266316	-3.609247377279859
C	-4.378358036899172	2.866462360594738	-3.447234822297753
H	-4.10634271209199	3.042710941226021	-4.48166277226628
C	-3.468728735758551	2.200987135832057	-2.596392809941774
C	-2.203426748938691	1.754980424235831	-3.015489735763675
H	-1.899498739328031	1.931003313573584	-4.038612887354921
C	-1.359976511614206	1.121721902231646	-2.140462249179792
C	-0.01824501729549672	0.6605926787291667	-2.512434950916393
C	0.4817824490667824	0.7264095862799352	-3.787041401434702
H	-0.1230407470131745	1.118646591073996	-4.593612221254457
C	1.783479099821239	0.2780379653931098	-4.071161829436831
C	2.347824843120327	0.3072217645920173	-5.365585833815256
H	1.761832927559798	0.6925704049691234	-6.191994422088704
C	3.622713063068739	-0.1525423362894255	-5.562329286576039
H	4.054548323476805	-0.1323788859786376	-6.556309515466841
C	4.388417037766485	-0.6562377893677332	-4.488377113651005
H	5.394308928850761	-1.014394626269369	-4.672973123771951
C	3.867364508486003	-0.6958141573343313	-3.223514558866063

H	4.446312734937969	-1.082510559177432	-2.392379184937099
C	2.554916147170921	-0.230109285010957	-2.997186517892855
C	1.960413925012303	-0.26628564356595	-1.7255621992893
H	2.526526438672298	-0.6588921698128807	-0.8899822557854268
N	-1.721971209636388	0.8960550899409376	-0.8299180173908062
N	0.7347211632291468	0.1464650390181449	-1.483104476989038
C	-1.845296623246403	-1.922477263098712	2.772395908558176
H	-0.8846884645075135	-2.357346109867444	3.043471661669178
H	-2.280987263610525	-2.460060610957463	1.933930936543366
H	-2.533878589227942	-1.960646866782089	3.613296643274097
S	-1.673802141698928	-0.2010169341142989	2.261105957526909
C	-0.8691121165985981	0.5154409185747081	3.72972950747809
H	0.1926272029382035	0.2662976853549588	3.724018072673569
H	-0.9719533058539879	1.59397919133606	3.600518103505578
C	-1.459294903964237	0.06171005892970272	5.053209670958381
H	-1.217232791103629	-0.9847746283939892	5.235726672069032
H	-0.9774779337303118	0.6479115885820277	5.842142915206382
O	-2.870901701229117	0.1610228170340286	5.131215462211711
H	-3.109650250974828	1.095982201588678	5.115647872970135
C	4.555317496604483	-2.613200219557823	3.179299539713295
C	5.495611468742798	-3.110518769450443	3.738768862794749
H	6.330862662385483	-3.553482201032657	4.23575063131157

Table S2. Nuclear coordinates (Å) of [**4**]$^{2+}$ minimized at the DFT/PBE0/TZP/COSMO level in water.

Ru	-0.1386272406317239	-0.1728871042070829	0.5035144302613997
C	-1.531207832588218	-2.757551541285255	-0.5218677891833204
H	-2.24524195140538	-2.072423656384397	-0.9594936734264196
C	-1.675997338141558	-4.126788522859417	-0.6665537013911703
H	-2.518821125742265	-4.516548561628556	-1.221494992827352
C	-0.7357222835419303	-4.963510374135509	-0.09270650390146704
H	-0.8206172645433322	-6.039053545348915	-0.1819074272165476
C	0.327600867434155	-4.40433911662796	0.5972036675306582
H	1.081112834402843	-5.035730075210719	1.047221963386152
C	0.417181389439417	-3.026298802234863	0.7064797035326705
C	1.523544100393143	-2.343645777494022	1.399681667297997
C	2.60281546127147	-2.953909223144666	2.013882645173779
H	2.693778989773381	-4.030211278213327	2.045507268159674
C	3.586680046685451	-2.153612667407497	2.600332753500335
C	3.467366308803155	-0.7634784683642144	2.545008518012292
H	4.228782042746849	-0.140463455060503	2.991546172989982
C	2.364411606499144	-0.208128963985455	1.920371284871431
C	2.073021538164229	1.227764094915739	1.792468720753408
C	2.913329210355384	2.210394634842851	2.291747916711746
H	3.841052838678216	1.938060530988706	2.775454190403192

C	2.551363903356648	3.540969230760397	2.171238195072737
H	3.197464461409832	4.319428153682459	2.557460879337389
C	1.349901365986842	3.852472938321198	1.559496479888117
H	1.016793496740082	4.876031773882573	1.450620045206895
C	0.5603224057869644	2.825035087576484	1.074174813912468
H	-0.3838218829951246	3.03477308698064	0.5916879604034408
N	-0.5216293835766055	-2.206354272701332	0.155047587701827
N	1.42392386809426	-1.001338278164124	1.379606451031281
N	0.902153017557386	1.537635809657449	1.171604041385557
C	-3.055834875307845	0.4626045292003551	-0.1810695788521076
H	-3.209630041363103	-0.2996710710699634	0.5728062088056303
C	-4.167432105358489	1.128597058235949	-0.7100348312821501
C	-5.487296559209559	0.8327259701647999	-0.295188126423042
H	-5.649178011916466	0.05867265660458093	0.4465769691020724
C	-6.535715999421286	1.52738318379346	-0.8292910015529869
H	-7.549881263621116	1.310463002625711	-0.5151715060764455
C	-6.306593740841212	2.535264578067364	-1.795257130406514
H	-7.15257921015576	3.073712147075788	-2.207278291116568
C	-5.040420298126659	2.839731075786094	-2.214178389097393
H	-4.87024200718328	3.61229575551311	-2.955176724920513
C	-3.935014326520808	2.141688821798193	-1.675169778541516
C	-2.606826972902733	2.373549805060031	-2.057235752150853
H	-2.380759725806299	3.106536976228194	-2.822597109822626
C	-1.583380804479718	1.665282006510563	-1.475154195189084
C	0.6905300303639436	1.006677217215075	-2.179636241328131
C	1.44494098073945	1.188420920758492	-3.313804367195852
H	1.251664988497113	2.043527364600589	-3.950555883023393
C	2.443378017206619	0.2660112487094222	-3.657204325507613
C	3.263160180950746	0.3825685116658833	-4.804067621013719
H	3.141168472402774	1.237076690469951	-5.45994451635968
C	4.196855512988538	-0.5806372607944117	-5.07424441519364
H	4.824449937422036	-0.4888223908618392	-5.953518405341535
C	4.362405104514678	-1.6998334163743	-4.225019509237881
H	5.111492072668192	-2.445424680994455	-4.46387209501423
C	3.582462178063681	-1.841231656677604	-3.112471637118505
H	3.69794148806238	-2.69522755607542	-2.453977974064316
C	2.608949485465777	-0.8600149567651078	-2.811330471509063
C	1.792650759123127	-0.9500685301219021	-1.679588086642163
H	1.914030288175524	-1.799387189712885	-1.020944257503333
N	-1.797857072116594	0.7287145290616694	-0.5059219985511247
N	0.8825410455729213	-0.05063428862247677	-1.341413318084977
C	-0.9111555926772327	-1.705518949397671	3.524696615773443
H	0.1164597094902461	-1.580235719766127	3.861243941079369
H	-1.006703992411256	-2.604271538494385	2.920455738034669
H	-1.585396778980701	-1.785680575273563	4.374319552610008

S	-1.451298510998732	-0.3169425161030515	2.509487491827477
C	-1.105690736513914	1.072592885094073	3.63265678449639
H	-0.03282458910066699	1.265640227898179	3.659755221938439
H	-1.599986077124376	1.928814860253459	3.17030927504548
C	-1.595596377644129	0.8542962781429553	5.05325523832981
H	-0.982706794336715	0.1042891559679136	5.552218599118054
H	-1.457715785829314	1.796290445726972	5.593716128308011
O	-2.93425964451962	0.3989196147537361	5.149703473115839
H	-3.516598099268223	1.102244418313223	4.837021328797476
C	4.704407288681812	-2.748790957023131	3.249952879084972
C	5.64754672222498	-3.246019440854954	3.804782716088554
H	6.486489020537687	-3.687566003023037	4.296774628787286
N	-0.2688007680057821	1.954491933437816	-1.836436878591254
H	-0.2315834179832435	2.747514473869215	-2.461243340831531

5. Photochemistry

5.1. Irradiation experiments monitored with MS and UV-vis

Photoreactions monitored with UV-vis spectroscopy were performed using a Cary 60 spectrometer from Varian equipped with temperature control set to 310 K and a magnetic stirrer. The measurements were performed in a quartz cuvette with 1 cm optical pathlength containing 3 mL of solution. The stirred sample was irradiated perpendicularly to the axis of the spectrometer with the beam of a LED fitted to the top of the cuvette.

For photoactivation with green light, a LED light source (λ = 517 nm, $\Delta\lambda_{1/2}$ = 23 nm, 5.2 mW, 5.43 x 10^{-8} mol · s^{-1}) was used, an absorption spectrum was measured every 30 sec until the end of the experiment. $[Ru]_0$ = 0.074, 0.077, 0.061, and 0.127 mM for [**1**]$(PF_6)_2$, [**2**]Cl_2, [**3**]$(PF_6)_2$, and [**4**]$(PF_6)_2$, respectively. Mass spectrometry was performed at the beginning and at the end of the irradiation to confirm the nature of the reagent and products. Photosubstitution quantum yield calculations were performed using the Glotaran Software as described earlier.[5] The conditions are summarized in Table S3.

5.2. Singlet Oxygen quantum yield measurement

The quantum yield of singlet oxygen generation was determined in a previously reported custom-built setup.[6] For each measurement, 500 µL of sample, consisting of the compound in deuterated methanol ($A_{450} \leq 0.1$ for 4.0 mm pathlength), was placed in a stirred 104F-OS semi-micro fluorescence cuvette (Hellma Analytics, Müllheim, Germany) in a CUV-UV/VIS-TC temperature-controlled cuvette holder from Avantes. The irradiation of samples was done at 298 K using 450 nm fiber-coupled laser (Laser system LRD-0450; Laserglow, Toronto, Canada), at 50 mW optical power (4 mm beam diameter; 0.4 Wcm^{-2}) at a 90° angle with respect to the spectrometer at 50 mW optical power (4 mm beam diameter; 0.4 Wcm^{-2}). The excitation power was measured using a S310C thermal sensor connected to a PM100USB power meter (Thorlabs, Dachau, Germany). Infrared emission spectra were measured from 1000 nm to 1400 nm using an Avantes NIR256-1.7TEC spectrometer, The infrared emission spectrum was acquired within 9 s, after which the laser was turned off directly. UV-vis absorption spectra before and after emission spectroscopy were measured using an Avalight-DHc halogen-deuterium lamp (Avantes) as light source (turned off during emission spectroscopy) and an Avantes 2048L StarLine UV-vis spectrometer as detector. No difference in UV-vis absorption spectrum was found due to exposure

to the blue laser, showing that the singlet oxygen emission is that of the starting compound. All spectra were recorded with Avasoft 8.5 software from Avantes and further processed with Microsoft Office Excel 2010 and Origin Pro 9.1 software. Results for these measurements are shown in main paper Table 2.

Table S3: Conditions of the photoreactions used for photosubstitution quantum yield calculations using Glotaran.

	**[2]Cl₂**	**[4](PF₆)₂**
irradiation wavelength (λ in nm)	517	517
volume (V in L)	0.003	0.003
path length (l in m)	0.01	0.01
concentration (c in M)	$7.71 \cdot 10^{-5}$	$1.27 \cdot 10^{-4}$
photon flux (Φ in mol · s^{-1})	$5.3 \cdot 10^{-8}$	$5.2 \cdot 10^{-8}$
epsilon Ru-L (ε in $M^{-1} \cdot cm^{-1}$) at 517 nm	2531	4458
epsilon Ru-OH_2 (ε in $M^{-1} \cdot cm^{-1}$) at 517 nm	7536	8014

5.3. Molar extinction coefficient in water

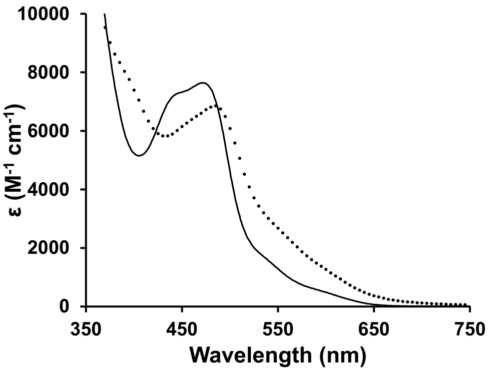

Figure S22: Molar extinction coefficient of aqueous solutions of [**2**]Cl_2 (—) and [**4**]$(PF_6)_2$ (···) in water.

5.4. Singlet oxygen production and phosphorescence

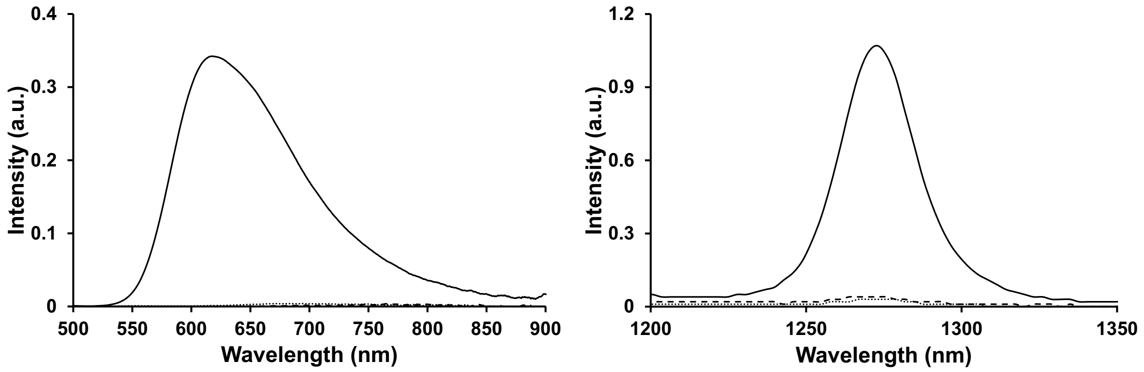

Figure S23: Visible emission spectra of [**2**]Cl_2 (···), [**4**]$(PF_6)_2$ (- -), and $[Ru(bpy)_3]Cl_2$ (—) (left) and near-infrared spectra of $^1O_2$ phosphorescence (λ_{em} = 1275 nm) sensitized by [**2**]Cl_2 (···), [**4**]$(PF_6)_2$ (- -), and $[Ru(bpy)_3]Cl_2$ (—) (right) in aerated methanol-d_4 at 293 K under blue-light irradiation (450 nm, 0.4 W · cm^{-2}).

5.5. Green light activation according to UV-vis spectroscopy and mass spectrometry

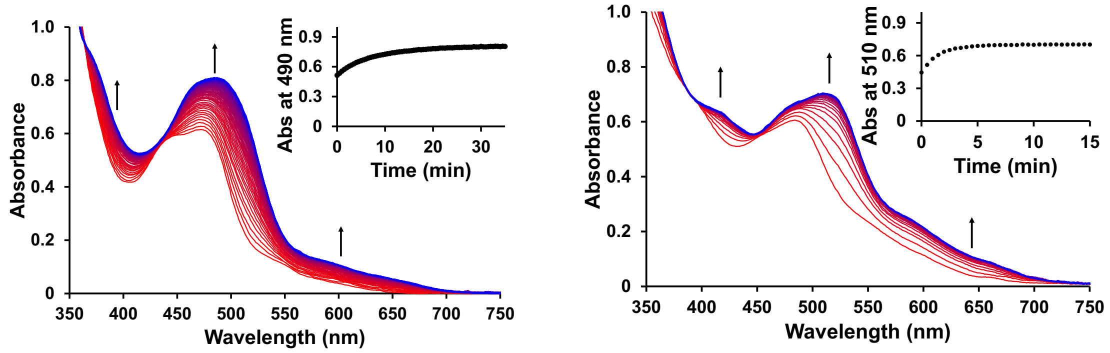

Figure S24: Evolution of the UV-vis absorption spectra of a solution of [**2**]Cl_2 (left) and [**4**]$(PF_6)_2$ (right) in water upon green light irradiation. Conditions: [Ru] = 0.077 and 0.127 mM for [**2**]Cl_2 and [**4**]$(PF_6)_2$, respectively, T = 37 °C, light source: λ = 517 nm, $\Delta\lambda_{1/2}$ = 23 nm, 5.2 mW, photon flux Φ_{517} = 5.3 x 10^{-8} and 5.2 x 10^{-8} mol · s^{-1}, V = 3 mL, under air atmosphere. Inset: Time evolution of absorbance at wavelength 490 nm for [**2**]Cl_2 and 510 nm for [**4**]$(PF_6)_2$.

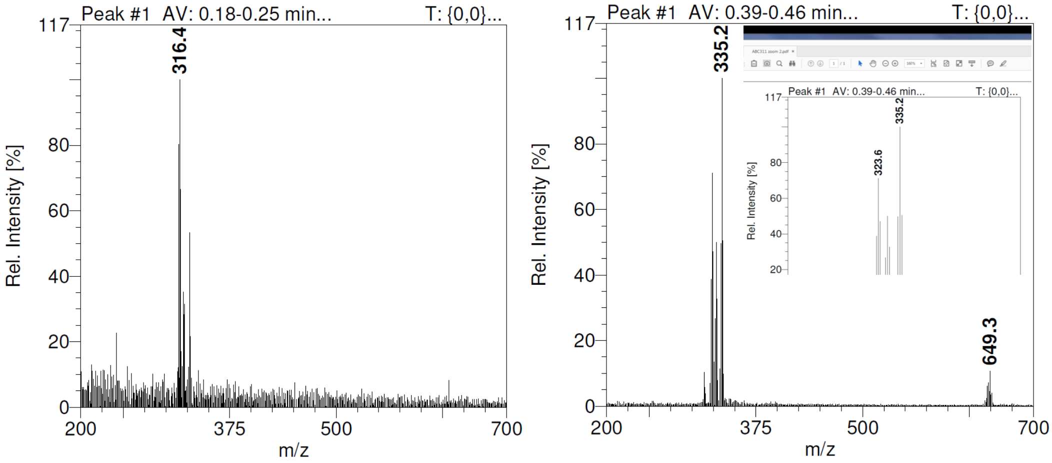

Figure S25: Mass spectrum of a solution of [**2**]Cl_2 and [**4**]$(PF_6)_2$ in water after 80 and 50 min, respectively, of light irradiation at 310 K with a 517 nm LED with a photon flux Φ_{517} = 5.3 x 10^{-8} and 5.2 x 10^{-8} mol · s^{-1}, respectively, under air atmosphere with peaks corresponding to a) $[Ru(HCC\text{-}tpy)(i\text{-}biq)(OH_2)]^{2+}$ (calc. m/z = 316.5); and b) $[Ru(HCC\text{-}tpy)(i\text{-}Hdiqa)(OH_2)]^{2+}$ (calc. m/z = 324.1) and

$[Ru(HCC\text{-}tpy)(i\text{-}Hdiqa)(OH)]^{+}$ (calc. m/z = 647.1). $[Ru(HCC\text{-}tpy)(i\text{-}Hdiqa)(MeCN)]^{2+}$ (calc. m/z = 335.6).

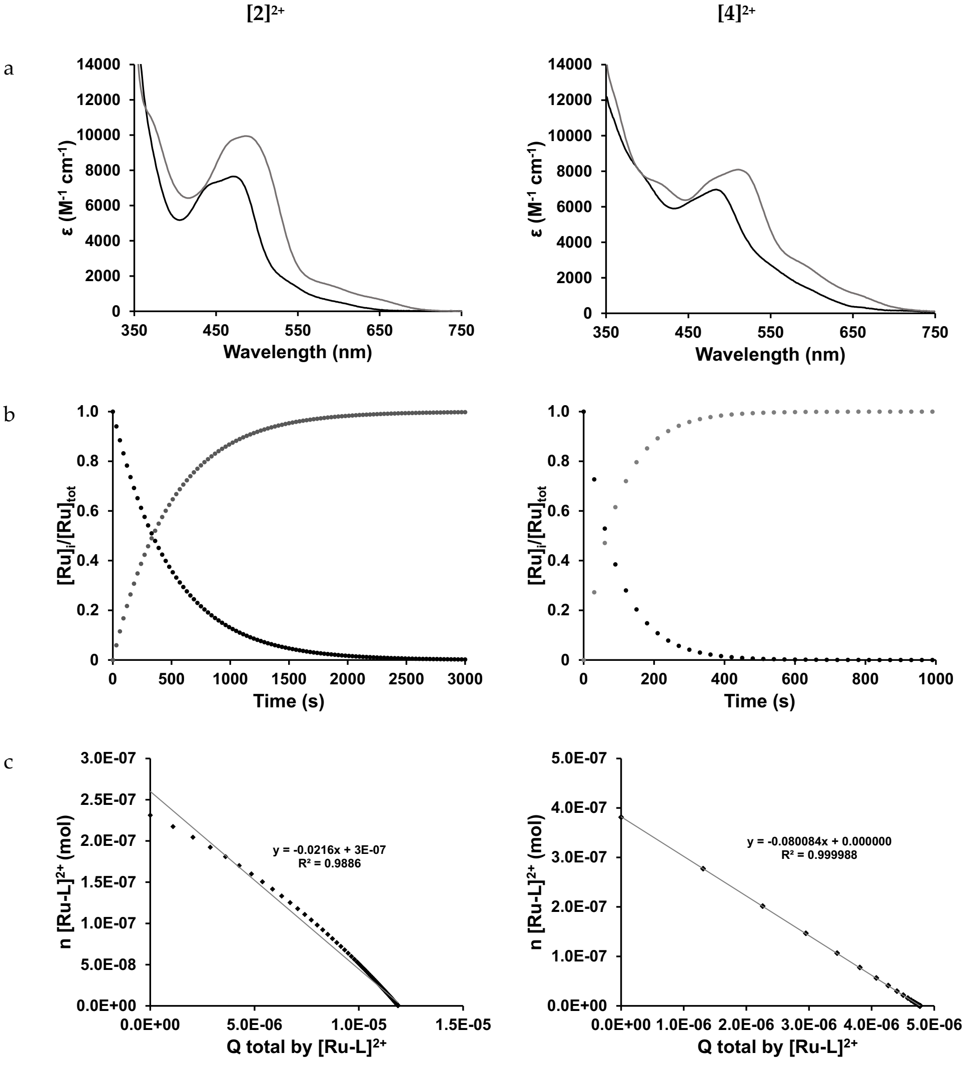

Figure S26: Kinetic data for the photosubstitution of Hmte according to the time evolution of the absorbance spectra of solutions of [**2**]Cl_2 and [**4**]$(PF_6)_2$ in H_2O under air atmosphere. a) Globally fitted absorption spectra of the starting material [**2**]Cl_2 and [**4**]$(PF_6)_2$ (black) and their aqua products $[Ru(HCC\text{-}tpy)(i\text{-}biq)(H_2O)]^{2+}$ and $[Ru(HCC\text{-}tpy)(i\text{-}Hdiqa)(H_2O)]^{2+}$ (grey). b) Modelled evolution of the relative concentration of [**2**]$^{2+}$ and [**4**]$^{2+}$ vs. irradiation time according to global fitting using Glotaran. c) Plot of the amount of [**2**]$^{2+}$ and [**4**]$^{2+}$ (mol) vs. total amount of photons absorbed by [**1**]$^{2+}$ and [**3**]$^{2+}$ since t = 0 (mol). The slope of the obtained line is the opposite of the quantum yield of the formation of the aqua complex. Conditions: 0.077 and 0.127 mM solution of [**2**]Cl_2 and [**4**]$(PF_6)_2$ in MilliQ H_2O irradiated at 310 K under air atmosphere using a 517 nm LED.

6. Cytotoxicity and cellular uptake

6.1. Materials

Human cancer cell line A549 (human lung carcinoma) and A431 (human epidormoid carcinoma) were distributed by the European Collection of Cell Cultures (ECACC) and purchased from Sigma Aldrich. Dulbecco's Modified Eagle Medium (DMEM, without phenol red, without glutamine), Glutamine-S (GM; 200 mM), trichloroacetic acid (TCA), glacial acetic acid, sulforhodamine B (SRB), and tris(hydroxylmethyl)aminomethane (Trisbase) were purchased from Sigma Aldrich. Fetal calf serum (FCS) was purchased from Hyclone. Penicillin and streptomycin were purchased from Duchefa and were diluted to a 100 mg/mL penicillin/streptomycin solution (P/S). Trypsin and OptiMEM (without phenol red) were purchased from Gibco Life Technologies. Trypan blue (0.4% in 0.81% sodium chloride and 0.06% potassium phosphate dibasic solution) was purchased from BioRad. Plastic disposable flasks and 96-well plates for cytotoxicity assays were purchased from Sarstedt. Cells were counted by using a BioRad TC10 automated cell counter with Biorad cell-counting slides. Cells were inspected with an Olympus IX81 microscope. UV-vis measurements for analysis of 96-well plates were performed with a M1000 Tecan Reader.

6.2. Cell culturing

Cells were cultured in Dulbecco's Modified Eagle Medium containing phenol red, supplemented with 9.0% v/v FCS, 0.2% v/v P/S and 0.9% v/v GM (called DMEM complete) and incubated at 37 ºC at 21% CO_2 in 75 cm^2 T-flasks. Fresh cells were passaged at least twice after being thawed and splitted once a week at 80-90% confluency. Cells were cultured for a maximum of 8 weeks for all biological experiment.

6.3. Phototoxicity essays

For each photocytotoxicity experiment, two parallel plates were prepared and treated identically, one plate was irradiated with green light, the second plate was kept in the dark. In each 96-well plate, A549 cells were seeded at t = 0 h at a density of 5000 and 8000 cells/well (100 µL), respectively in OptiMEM supplemented with 2.4% v/v FCS, 0.2% v/v P/S, and 1.0% v/v GM (called OptiMEM complete) and incubated for 24 h at 37 ºC and 21% O_2 and 7% CO_2. Only the inner 60 wells were used for seeding, the outer wells were kept cell free to prevent border effects during irradiation[7]. At t = 24 h, aliquots (100 µL) of six different concentrations of freshly prepared stock solutions of the compounds in OptiMEM complete were added to the wells in triplicate (see plate design in Figure S 26) and incubated for 24 h. Sterilized dimethylsulfoxide (DMSO) was used to dissolve the compounds in such amounts that the maximum v/v% of DMSO per well did not exceed 0.5%. At t = 48 h, the plates were irradiated with the cell-irradiation setup (520 nm, 30 min, 38 J/cm^2) and the control plate was kept in the dark. After irradiation, all the plates were incubated in the dark until a total time of t = 96 h after seeding. The cells were fixated by adding cold TCA (10% w/v; 100 µL) in each well and the plates were stored at 4 ºC for at least 4 h as part of the SRB assay that was adapted from Vichai *et al*.[8] After fixation, the TCA medium mixture was removed from the wells, rinsed three times with demineralized water. Afterwards, each well was stained with 100 µL SRB (0.6% w/v in 1% v/v acetic acid) and incubated for 30 min, the SRB was removed by washing with acetic acid (1% v/v), and air dried. The SRB dye was solubilized with Tris base (10 mM; 200 µL) overnight, and the absorbance in each well was read at λ = 510 nm.

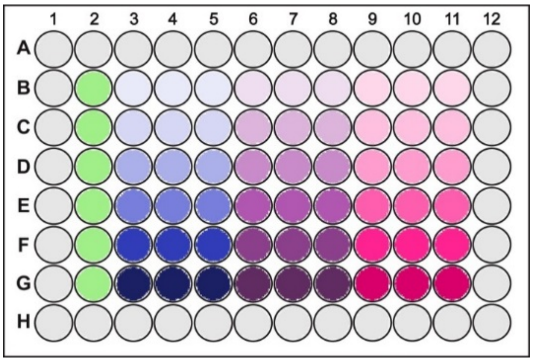

Figure S27: Design of a 96-well plate used in the (photo)cytotoxicity assays. Grey: Outer wells are not used for seeding to prevent border effects; green: non-treated cells (n_t = 6); blue: cells treated with compound A; purple: cells treated with compound B; pink: cells treated with compound C. Each compound was added in six different concentrations (one per row) per triplicate (n_t = 3).

The SRB absorbance data per compound per concentration was averaged over three identical wells (technical replicates, n_t = 3) in Excel and was exported to GraphPad Prism. Relative cell populations were calculated by dividing the average absorbance of the treated wells by the average absorbance of the untreated wells. It was checked that the cell viability of the untreated cells of the samples irradiated were similar (maximum difference of 10%) to the non-irradiated samples to make sure no harm was done by light alone. The resulting dose-response curve for each compound under dark and irradiated conditions was fitted to a non-linear regression function with fixed *y* maximum (100%) and minimum (0%) (relative cell viability) and a variable Hill slope. The data of three independent biological replications was used to obtain the effective concentrations (EC_{50} in μM). Photo indices (PI) were calculated, for each compound, by dividing the EC_{50} value obtained in the dark by the EC_{50} value determined under light irradiation.

6.4. Dark stability

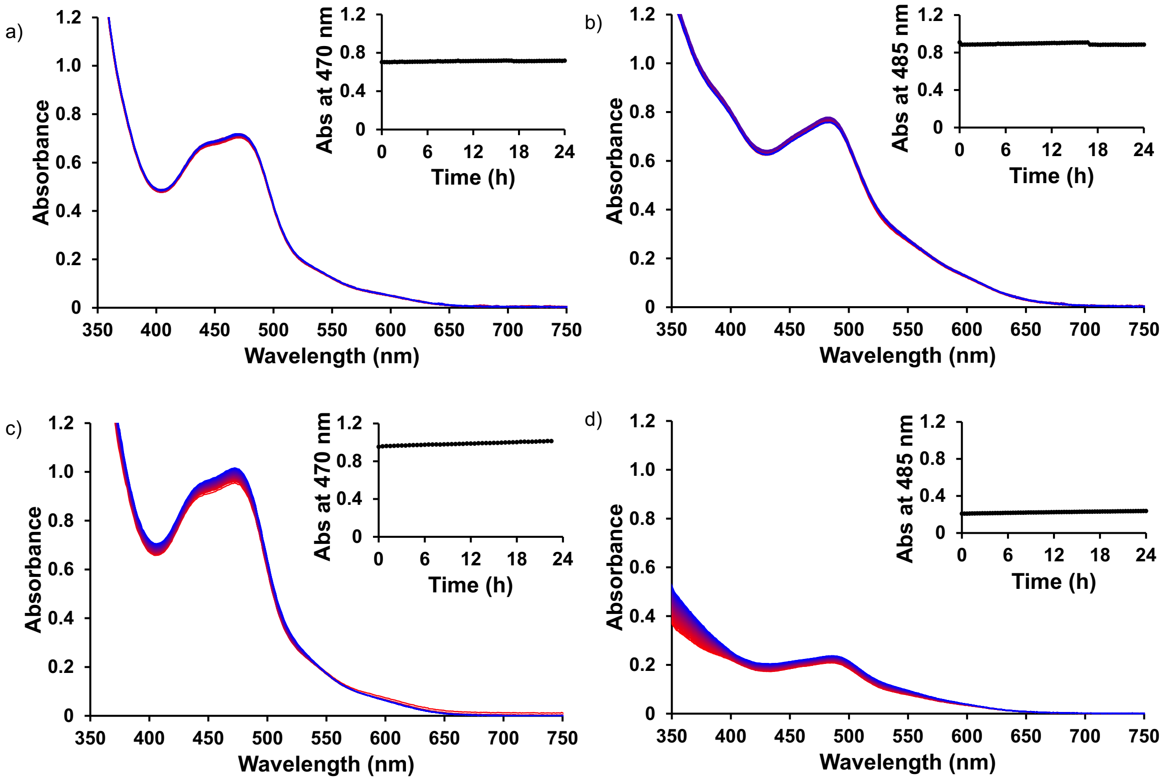

Figure S28: Evolution of the UV-vis spectra (region 350 – 750 nm) of a solution of a) [**2**]Cl_2 and b) [**4**]$(PF_6)_2$ in water, and c) [**2**]Cl_2 and d) [**4**]$(PF_6)_2$ in OptiMEM complete. Conditions: [Ru] = 0.094, 0.111, 0.130, and 0.035 mM, respectively, T = 37 °C, t = 24 h, V = 3 mL, under air atmosphere and in the dark. Inset: Time evolution of absorbance at wavelength 470 nm for [**2**]Cl_2 and 485 nm for [**4**]$(PF_6)_2$.

6.5. Determination of light dose

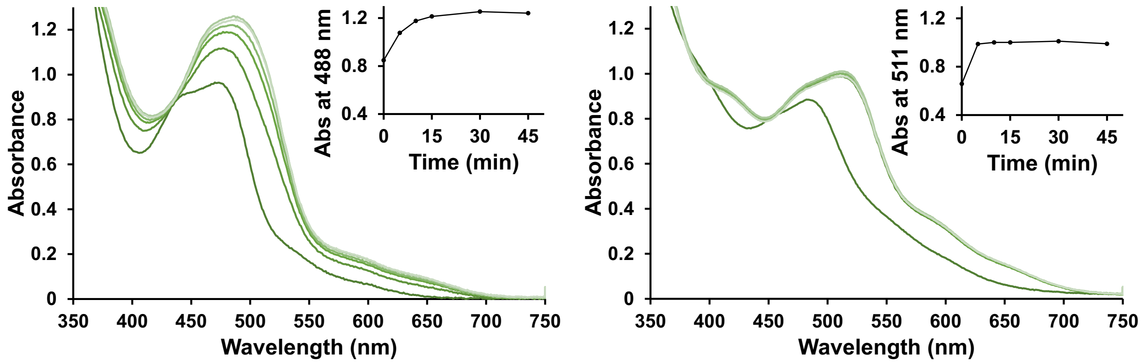

Figure S29: Evolution of the UV-vis spectra (region 350 – 750 nm) of a solution of [**2**]Cl_2 and [**4**]$(PF_6)_2$ in demineralized water upon green light irradiation in a 96 well plate i.e. under the conditions of the cytotoxicity experiment. Conditions: [Ru] = 250 µM, T = 37 °C, t = 0, 5, 10, 15, 30, and 45 min, light source: λ = 520 ± 20 nm, 20.9 ± 1.6 mW cm^{-2}, V = 200 µL, under air atmosphere. Inset: Time dependent absorbance at wavelength 488 nm for [**2**]Cl_2 and 511 nm for [**4**]$(PF_6)_2$.

6.6. Dose response curves for A549 cells

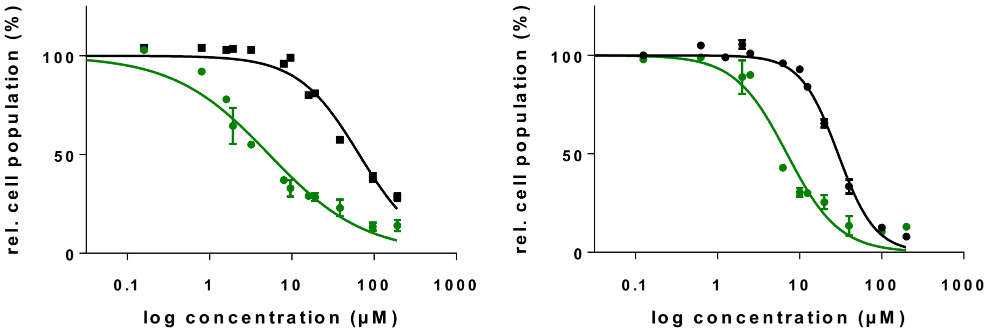

Figure S30: Dose response curves for A549 cells under normoxic conditions treated with [**2**]Cl_2 (left) or [**4**]$(PF_6)_2$ (right) and irradiated with green light (520 nm, 38 J · cm^{-2}) 24 h after treatment (green line) or left in the dark (black line).

6.7. Cell-irradiation setup

The cell-irradiation system consisted of a Ditabis thermostat (980923001) fitted with two flat-bottomed micro-plate thermoblocks (800010600) and a 96-LED array fitted to a standard 96-well plate. The 520 nm LED (OVL-3324), fans (40 mm, 24 VDC, 9714839), and power supply (EA-PS 2042-06B) were obtained from Farnell. See Hopkins *et al.* for a full description.[7]

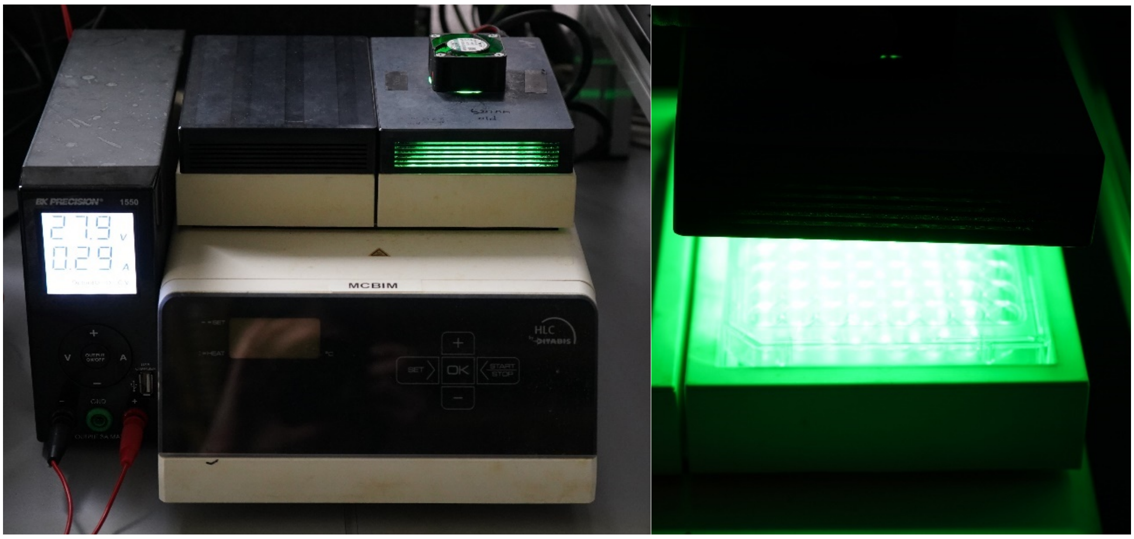

Figure S31: Left: shows irradiation setup with thermoblock in black, thermostat on the left and the LED array with green light. Right: 96-well LED array fitted to a standard 96-well plate.

6.8. Determination of irradiation times

To determine which light dose is necessary to fully activate the complexes during the cytotoxicity assay, the photochemical reactivity of the ruthenium-based complexes was tested. Therefore, the inner 60 wells of a 96-well plate were filled with OptiMEM complete (100 µL, seeding without cells), and aliquots of the complexes (at their highest concentration used in the cytotoxicity assay, 250 µM) were added to the first column. The plate was irradiated for a certain amount of time, hereafter a new column was filled, and the plate was irradiated again. This process was repeated several times (irradiation times: 15, 15, 5,

5, and 5 min), and was finished with the last column filled but not irradiated. In this way, the columns were irradiated cumulative for a total time of 0, 5, 10, 15, 30, and 45 min, respectively. The absorbance of each well was measured (between 350 and 700 nm) by a M1000Tecan Reader and corrected for the absorbance of OptiMEM complete. The data was analyzed using Excel and the absorbance as function of time was plotted to check the time necessary for full activation (shown in Figure S32).

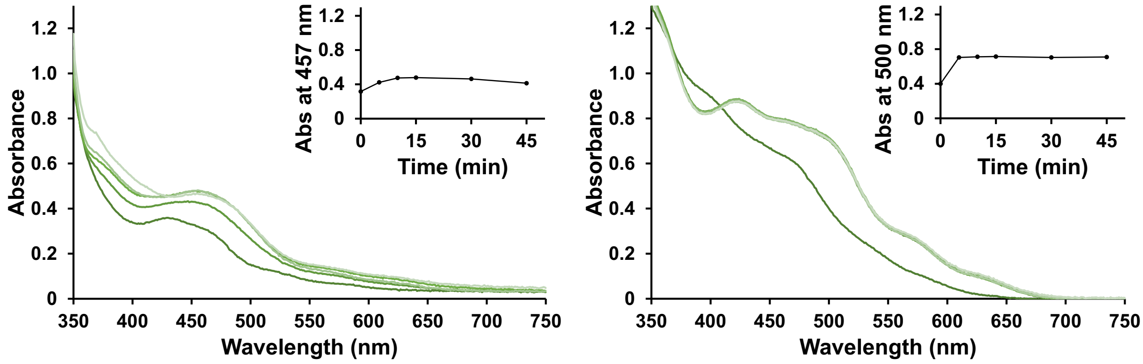

Figure S32: Evolution of the UV-vis spectra (region 350 – 750 nm) of solutions of [**1**]$(PF_6)_2$, and [**3**]$(PF_6)_2$ in demineralized water upon green light irradiation in a 96 well plate, i.e. under the conditions of the cytotoxicity experiment. Conditions: [Ru] = 250 µM, T = 37 °C, t = 0, 5, 10, 15, 30, and 45 min, light source: λ = 520 ± 20 nm, 20.9 ± 1.6 mW cm^{-2}, V = 200 µL, under air atmosphere. Inset: Time dependent absorbance at wavelength 457 nm for [**1**]$(PF_6)_2$, and 500 nm for [**3**]$(PF_6)_2$.

6.9. Cellular uptake

Cell uptake studies for the ruthenium-based complexes were conducted on A549 cancer cells at 37 °C and 21% O_2. Per compound, 1.6 x 10^6 cells were seeded in 10 mL OptiMEM complete in a 75 cm^2 flask at t = 0 h. At t = 24 h, the media was aspirated and the cells were treated with solutions of the complexes in 12 mL OptiMEM complete at a concentration of 30 µM. Treatment at the same concentration for all complexes allows for comparison of the amount of ruthenium taken up by the cells. 30 µM correlates to the lowest EC_{50} value of all complexes in the dark (EC_{50} value of [Ru(HCC-tpy)(*i*-Hdiqa)(Hmte)]$(PF_6)_2$). At t = 48 h, the medium was aspirated and the cells were washed twice with PBS (5 mL). The cells were trypsinized (2 mL, 5 min), suspended in OptiMEM complete (8 mL), and centrifuged (4 min, 1200 rpm). The supernatant was removed, the cells were resuspended in PBS (1 mL), and the cell count determined. The cells were centrifuged for a second time (4 min, 1200 rpm), the supernatant was aspirated, and the cell pellet stored at -80 °C.

For metal and protein quantification, the pellets were resuspended in demineralized water (200 µL) and lysed for 30 min by ultrasonication. The protein content of cell lysates was determined by the Bradford method. For the ruthenium measurements a contrAA 700 high-resolution continuum-source atomic absorption spectrometer (Analytik Jena AG) was used. All reagents were purchased from Sigma Aldrich. Stock solutions of the respective complexes in graded concentrations (solvent: DMSO) were used as standards and calibration was done in a matrix-matched manner. Meaning all samples and standards were adjusted to the same cellular protein concentration (1.0 mg cell protein per mL) by dilution (final DMSO concentration: 0.5 %). Triton X-100 (1%, 10 µL) as well as nitric acid (13%, 10 µL), were added to each standard sample (120 µL). Samples were injected (50 µL) into coated standard graphite tubes (Analytik Jena AG) and thermally processed as previously described by Kowalski *et al.*[9] Drying steps were adjusted and the atomization temperature set to 2400 °C. Ruthenium was quantified at a wavelength of 349.90 nm. The mean integrated absorbance of double injections was used throughout the measurements.

The data of three independent biological replications was used to obtain the uptake values, calculated as nmol metal (ruthenium) per mg cell protein.

7. Click reactions

7.1. Materials

Black 96-well Screenstar plates (Product number #655866, Greiner Bio-One, Frickenhausen, Germany) were used for immunostaining; copper sulfate, sodium ascorbate, Triton X-100, tris(3-hydroxypropyl-triazolylmethyl)amine (THPTA), phosphate buffered saline (PBS), and bovine serum albumin (BSA) were purchased from Sigma Aldrich; paraformaldehyde (PFA 16%) from Alfa Aesar; and Alexa Fluor™ 488 Azide (A10266) from Invitrogen (Thermo Fisher Scientific). Azidoplatin was kindly provided by the DeRose lab.

7.2. Treatment

A549 cells were seeded at t = 0 h in 96-well plates at a density of 5000 cells/well (100 µL) in OptiMEM complete and incubated for 24 h at 37 °C and 21% O_2 and 7% CO_2. At t = 24 h, the cells were treated with aliquots (100 µL) of either [**2**]Cl_2 (50 µM), [**4**]$(PF_6)_2$ (50 µM) and incubated for another 24 h. At t = 48 h (DLI=24 h), the plate was irradiated without changing the medium under an air atmosphere using the cell-irradiation system (520 nm, 1 h, 76 J/cm^2) and further incubated in the dark in a standard incubator. At t = 49 h, ie 1 h after light irradiation, the wells were washed twice with 1X PBS (100 µL) and fixed with 4% PFA in PBS (100 µL) for 20 min. Then, PFA was aspirated, and 0.5% Triton X-100 in PBS (100 µL) was added for 20 min. After aspiration, the wells were washed twice with BSA 3% (100 µL) for 10 min.

7.3. Click reaction

Before removing the 3% BSA solution, the click cocktail was prepared. Herefore, stock solutions of the Alexa Fluor^{TM} 488 Azide (c=2mM in DMSO), Sodiumascorbate (NaAsc, c=83 mM in PBS) and Tris((1-hydroxy-propyl-1H-1,2,3-triazol-4-yl)methyl)amine (THPTA, c=15 mM in PBS) were prepared. For the $CuSO_4$ x 5 H_2O, a stock of c=3 mM in THPTA (c=15 mM) was used to dissolve the $CuSO_4$ x 5 H_2O and prepared freshly before using. For a positive click reaction, 33 µL of a 15 µM Alexa Fluor^{TM} 488 solution dissolved in PBS, 33 µL of the 3 mM $CuSO_4$ x 5 H_2O, dissolved in 15 mM THPTA and 33 µL of 83 mM NaAsc solution was added. For a negative click reaction, the 33 µL of the 3 mM $CuSO_4$ x 5 H_2O, dissolved in 15 mM THPTA was exchanged for 33 µL of 15 mM THPTA solution.

The click mixture was added to the cells and kept at room temperature in dark for 1h. Thereafter, the mixture was aspirated and the wells were washed with 3% BSA in PBS for 10 min, 0.5% Triton-X-100 for 10 min and finally PBS for 10 min. Depending if the immunostaining (co-staining) is applied in the experiment, it is carried out at this time point. If not, Hoechst was added and the cells were imaged.

8. Imaging

8.1. Materials

GM130 (D6B1) XP Rabbit mAb, LAMP1 (D2D11) XP Rabbit mAb, Anti rabbit IgG (H+L) F(ab‘) 2 Fragment Alexa Fluor^{TM} 647 Conjugate and Anti mouse IgG (H+L) F(ab‘) 2 Fragment Alexa Fluor^{TM} 647 Conjugate was purchased from Cell Signaling – Bioké. Anti-P4HB [RL90] Mouse (ab2792) and

Anti-ATP5A antibody [15H4C4] Mouse (ab14748) was purchased from Abcam. NucBlue™ from Invitrogen (R37605).

8.2. Co-staining

After the explained procedure for the cupper-click reaction in 7.3, co-staining is applied in the dark. To do that, wells were washed twice with Blocking Buffer (5% BSA in PBS with 0.3% Triton X-100 to block unspecific binding) for 60 min. After blocking, the primary antibodies were diluted in Dilution Buffer (1% BSA / 0.3% Triton™ X-100 buffer in PBS). Blocking solution was aspirated and solution of primary antibody was added and incubated overnight (Lysosomes staining: LAMP1 in 1:300; Golgi Apparatus staining: GM130 1:3200; Endoplasmic Reticulum staining: P4HB 1:1000; Mitochondria staining: ATP5A 1:1000) After incubation, the wells were rinsed three times with PBS, 0.1% Triton X-100 for 5 min. The secondary antibody was diluted in PBS, 0.1% Triton for 1h in dark (for Lysosomes and Golgi Apparatus Anti-Rabbit in 1:2000; Endoplasmic Reticulum and Mitochondria in 1:2000 dilution). The wells were rinsed with PBS, 0.1% Triton X-100. After aspiration, nuclear co-staining (NucBlue™, 1 drop/2 mL, 100 µL) was added and incubated for 60 min at room temperature. The plate was immediately taken to the microscope for imaging.

8.3. Microscopy imaging

Confocal imaging was performed on an Eclipse Ti2-C2+ Nikon confocal microscope using the 40x water (Apo LWD 40x/1.15 WI λS DIC N2) objective. Lasers used: 405 nm for Hoechst 33342 (ex./em. 360/460 nm), 488 nm for [**2**]$^{2+}$ and [**4**]$^{2+}$ labeled with Alexa Fluor™ 488 (ex./em. 495⁄519 nm), and 640 nm for Alexa Fluor™ 647 (ex./em. 651/667 nm). The settings for image acquisition (laser power and PMT gain) were identical for all conditions. The images were processed using NIS-Elements AR software version 5.02.02, Fiji Image J software while the figure were made using Omero 5.6 where the raw data are stored. The setting during image processing were identical for each condition. Hoechst, AlexaFluor488 and AlexaFluor647 were displayed in blue, green and red.

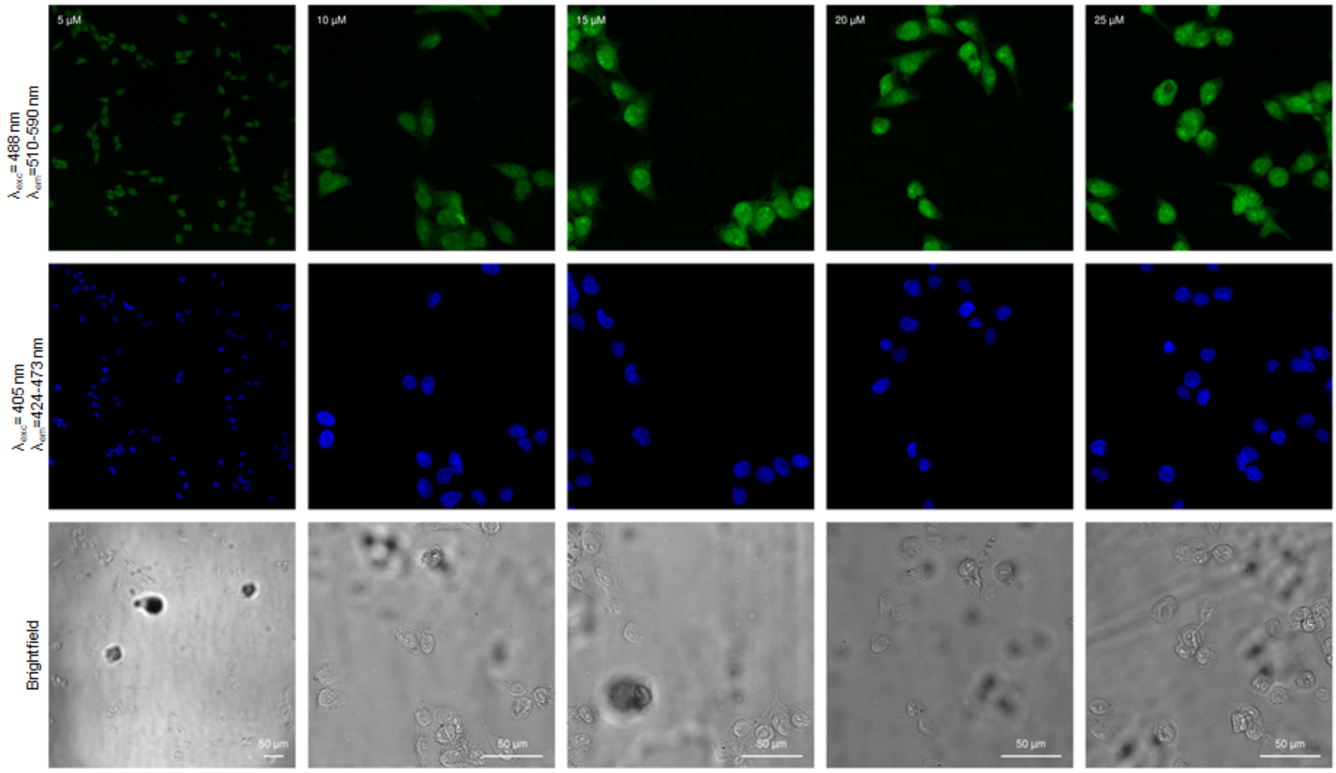

Figure S33: Concentration experiments of [**4**]$(PF_6)_2$ with 60 min incubation time after activation with λ=520 nm for 60 min (76 J/cm^2).

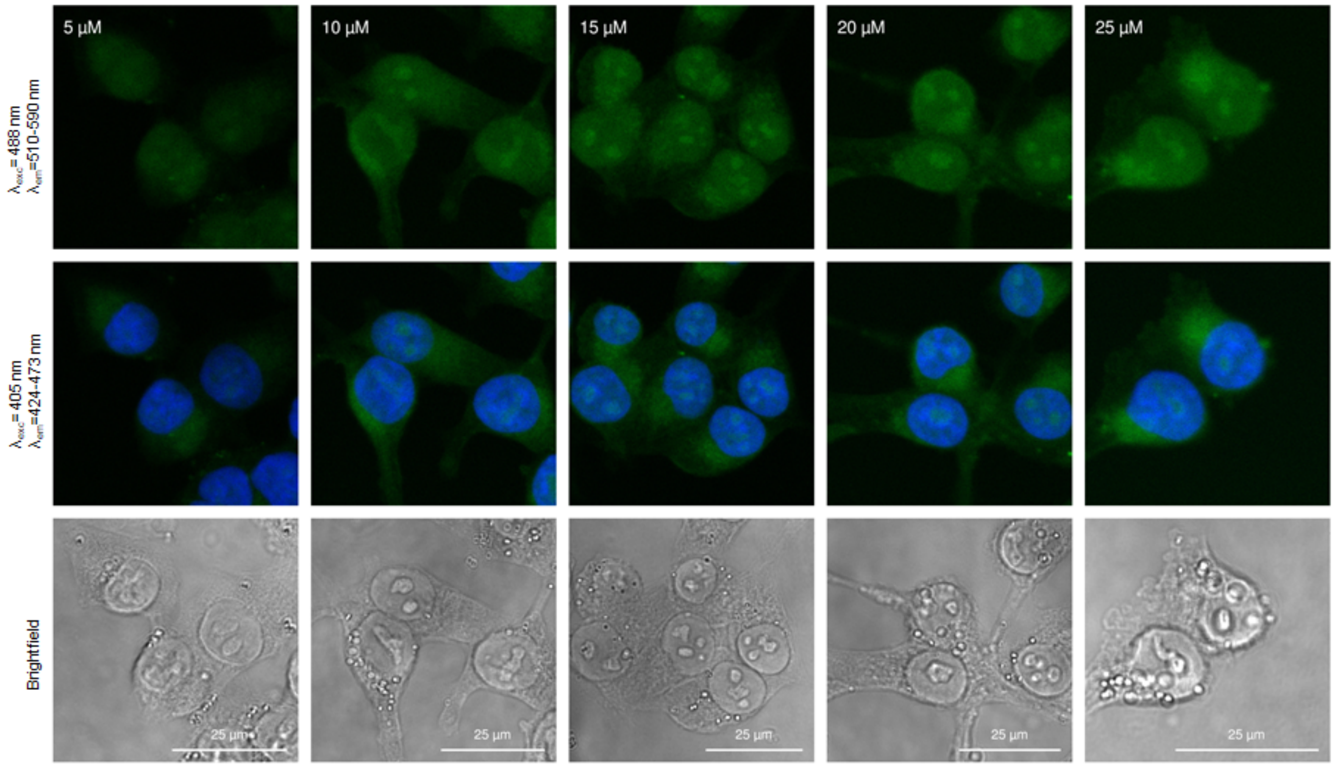

Figure S34: Concentration experiments of [**2**]Cl_2 with 60 min incubation time after activation with λ=520 nm for 60 min (76 J/cm^2).

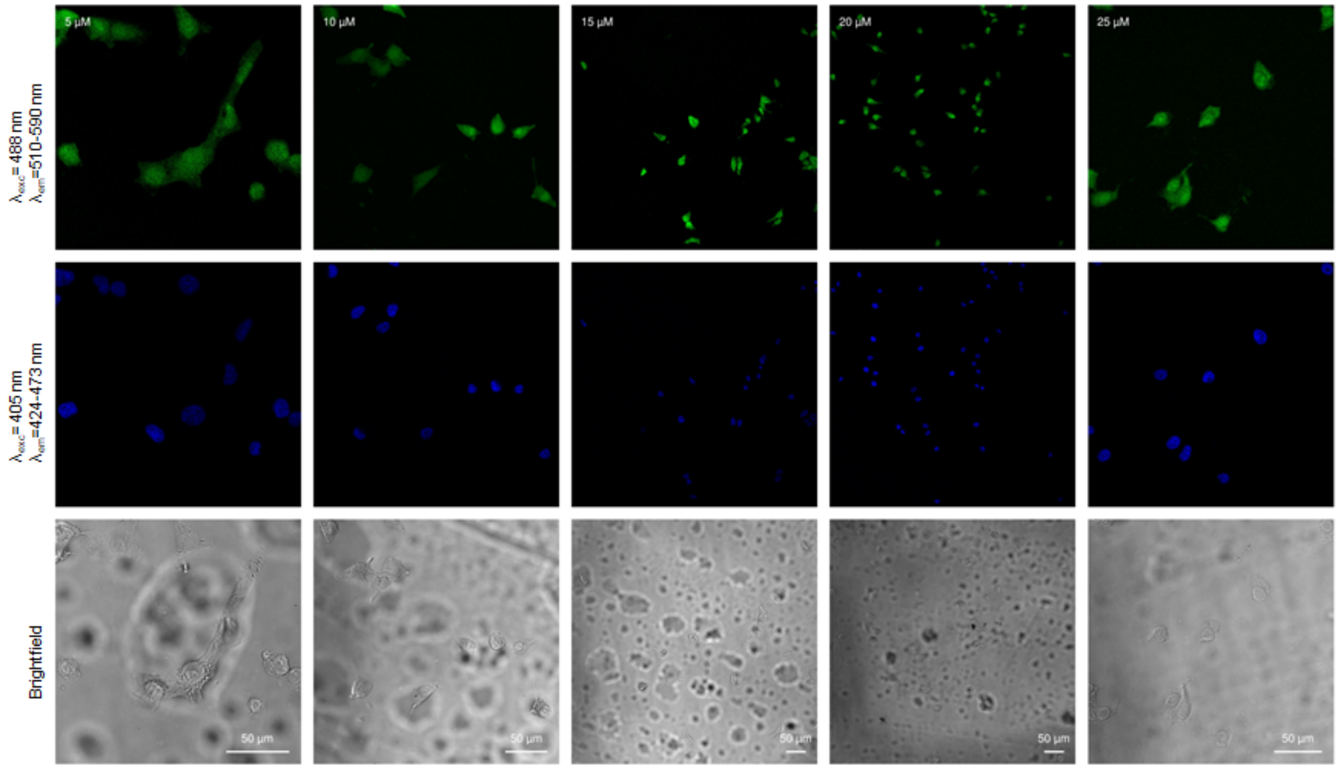

Figure S35: Concentration experiments of [**4**](PF_6) with 24 h incubation time after activation with λ=520 nm for 60 min (76 J/cm^2).

8.4. Images with co-staining using 25 µM concentration of the PACT prodrug

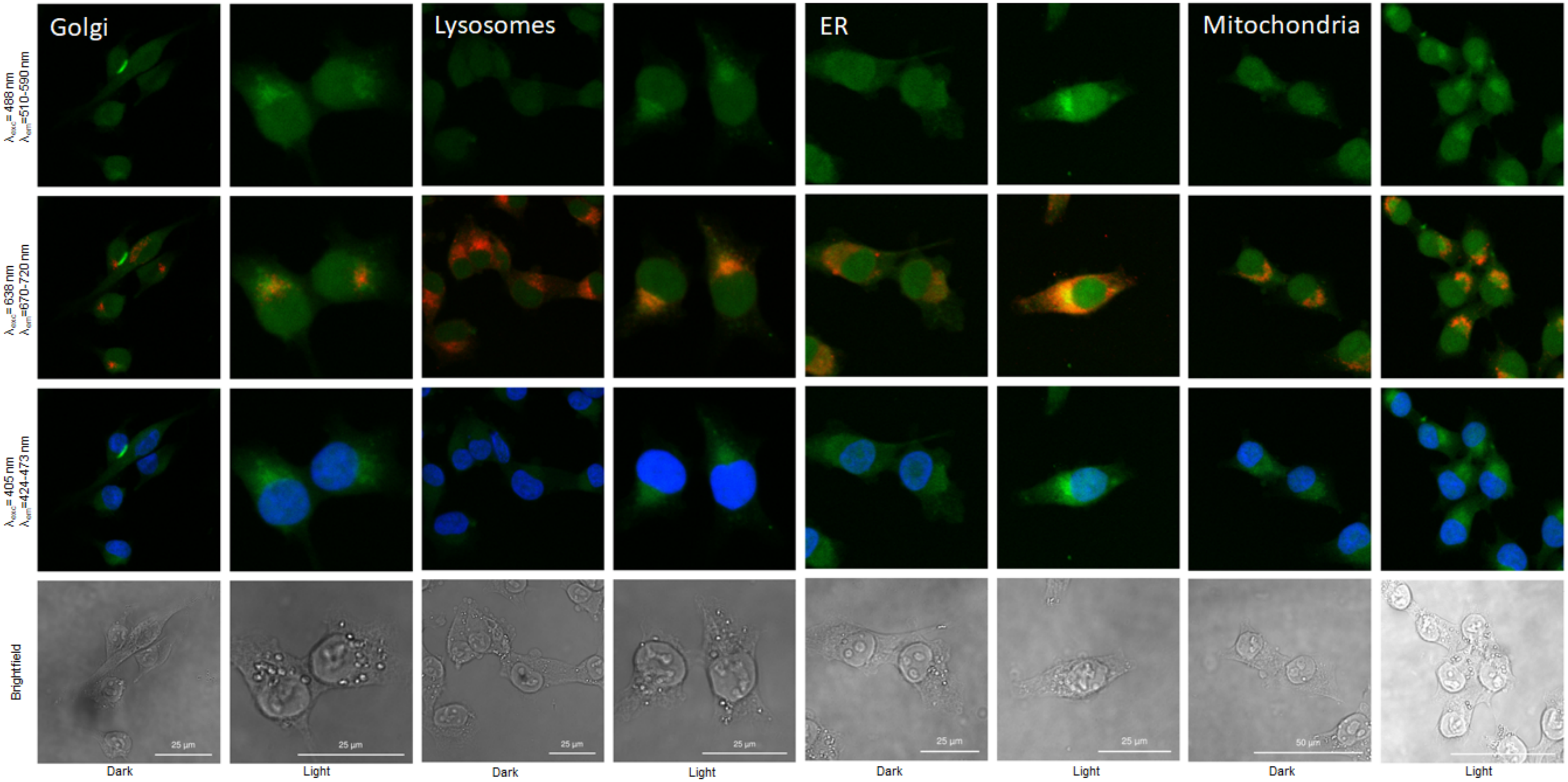

Figure S36: [**4**]$(PF_6)_2$ in 25 µM concentration; treatment: 30 min irradiation (λ=520 nm, 38 J/cm^2), 60 min incubation post-irradiation. Microscopy images taken with 40x water objective.

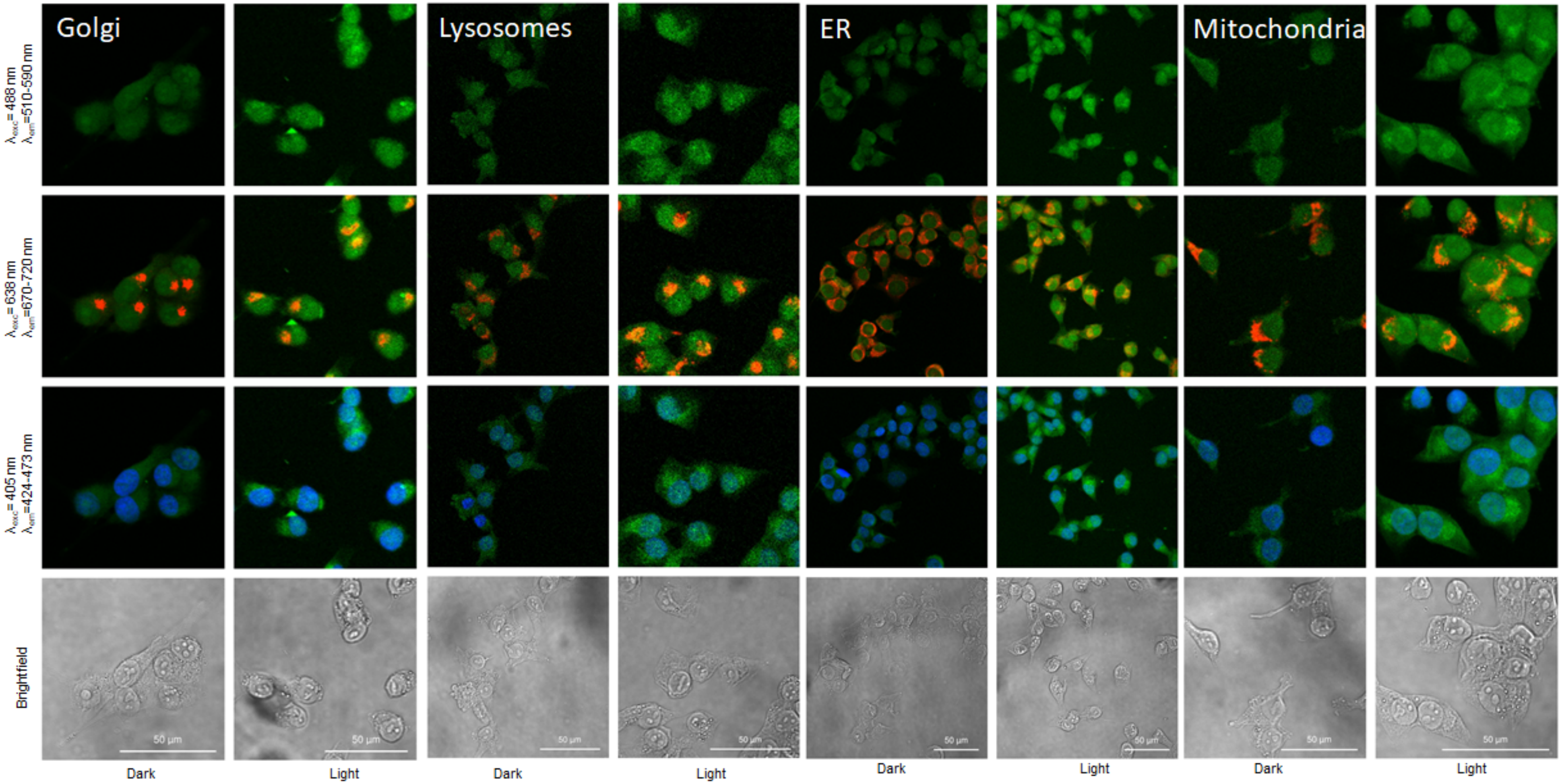

Figure S37: [**4**]$(PF_6)_2$ in 25 µM concentration; treatment: 60 min irradiation (λ=520 nm, 76 J/cm^2), 0 h incubation post-irradiation. Microscopy images taken with 40x water objective.

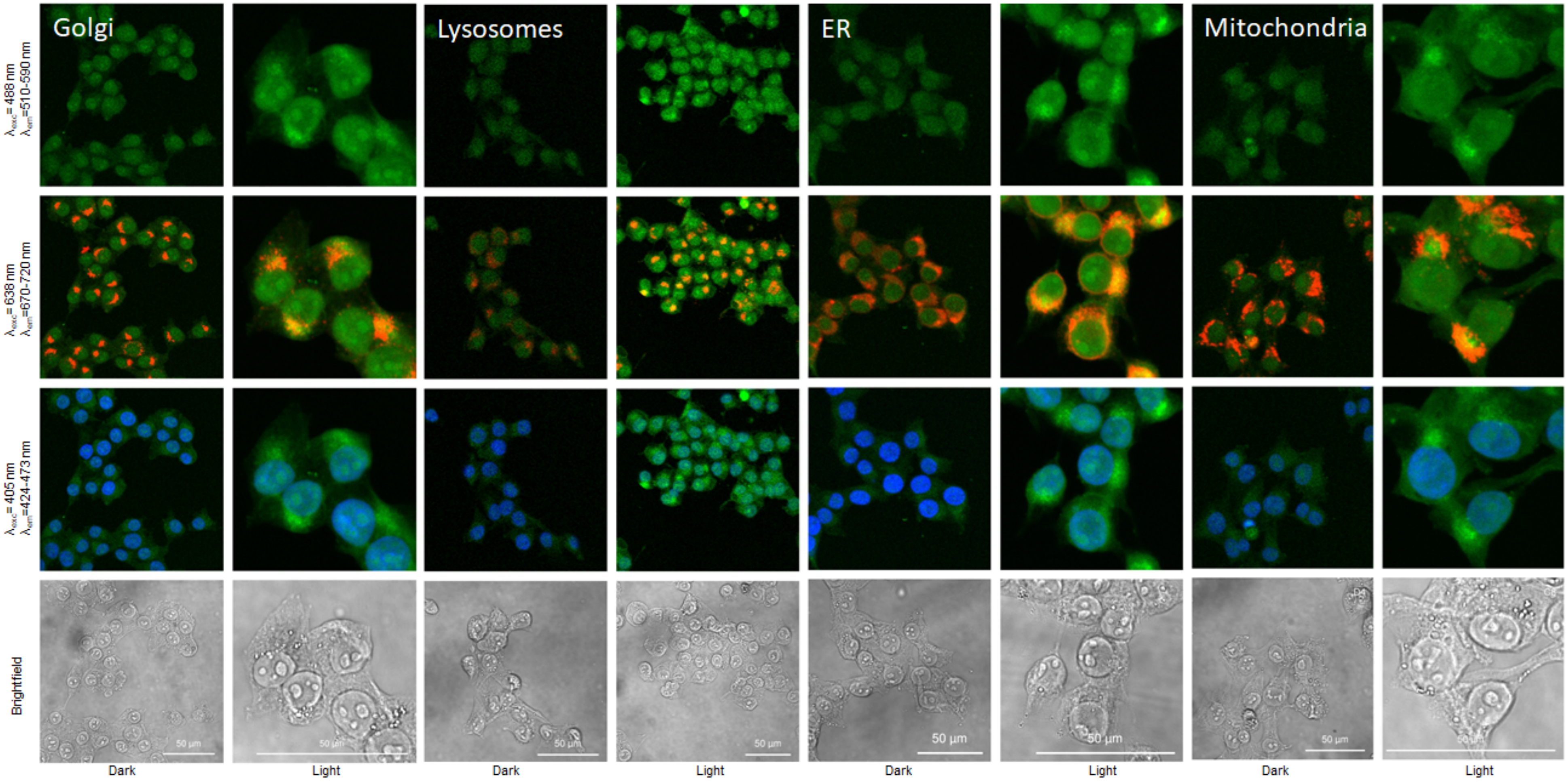

Figure S38: [**4**]$(PF_6)_2$ in 25 µM concentration; treatment: 60 min irradiation (λ=520 nm, 76 J/cm^2), 60 min incubation post-irradiation. Microscopy images taken with 40x water objective.

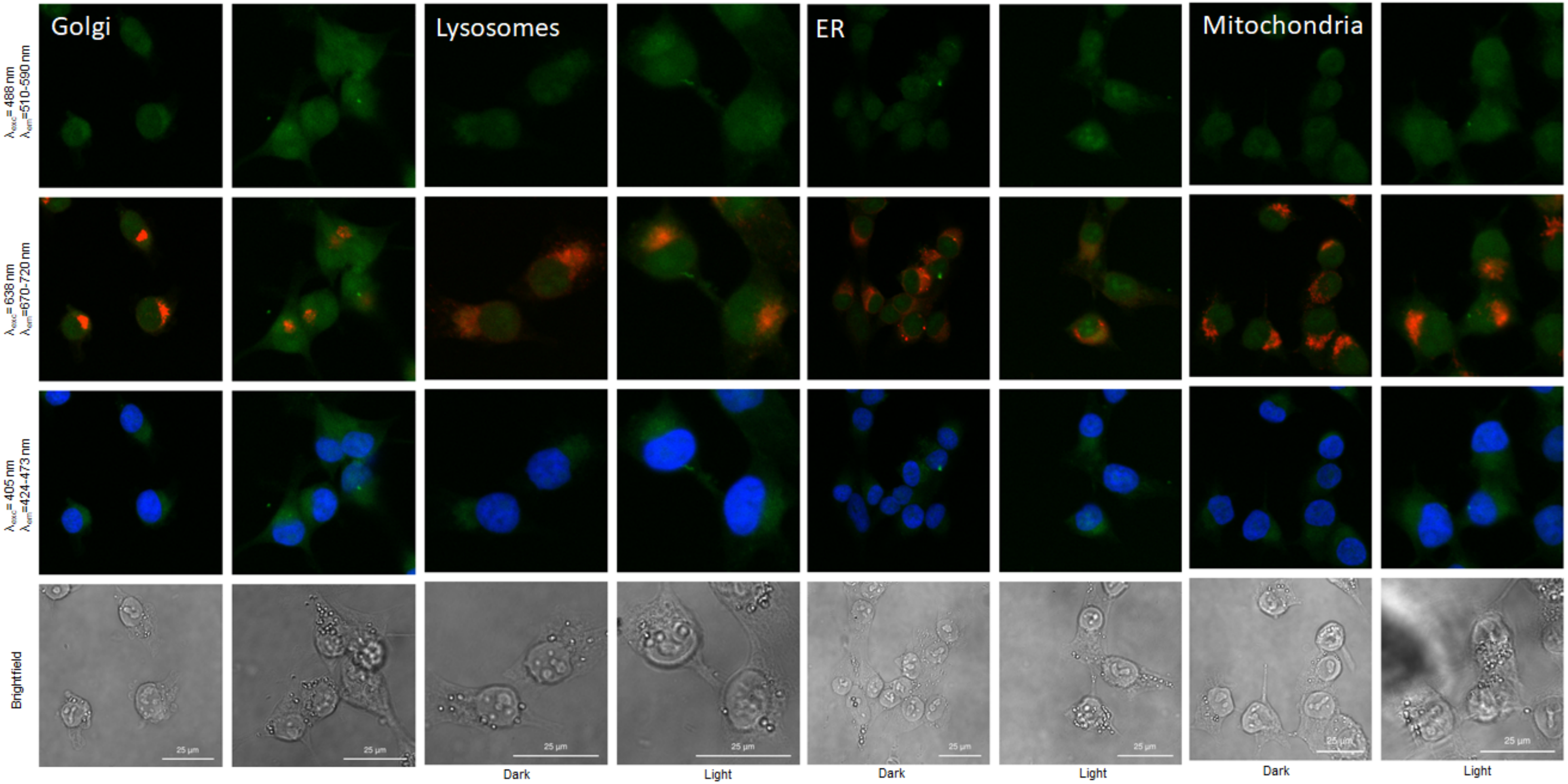

Figure S39: [**2**]Cl_2 in 25 µM concentration; treatment: 30 min irradiation (λ=520 nm, 38 J/cm^2), 60 min incubation post-irradiation. Microscopy images taken with 40x water objective.

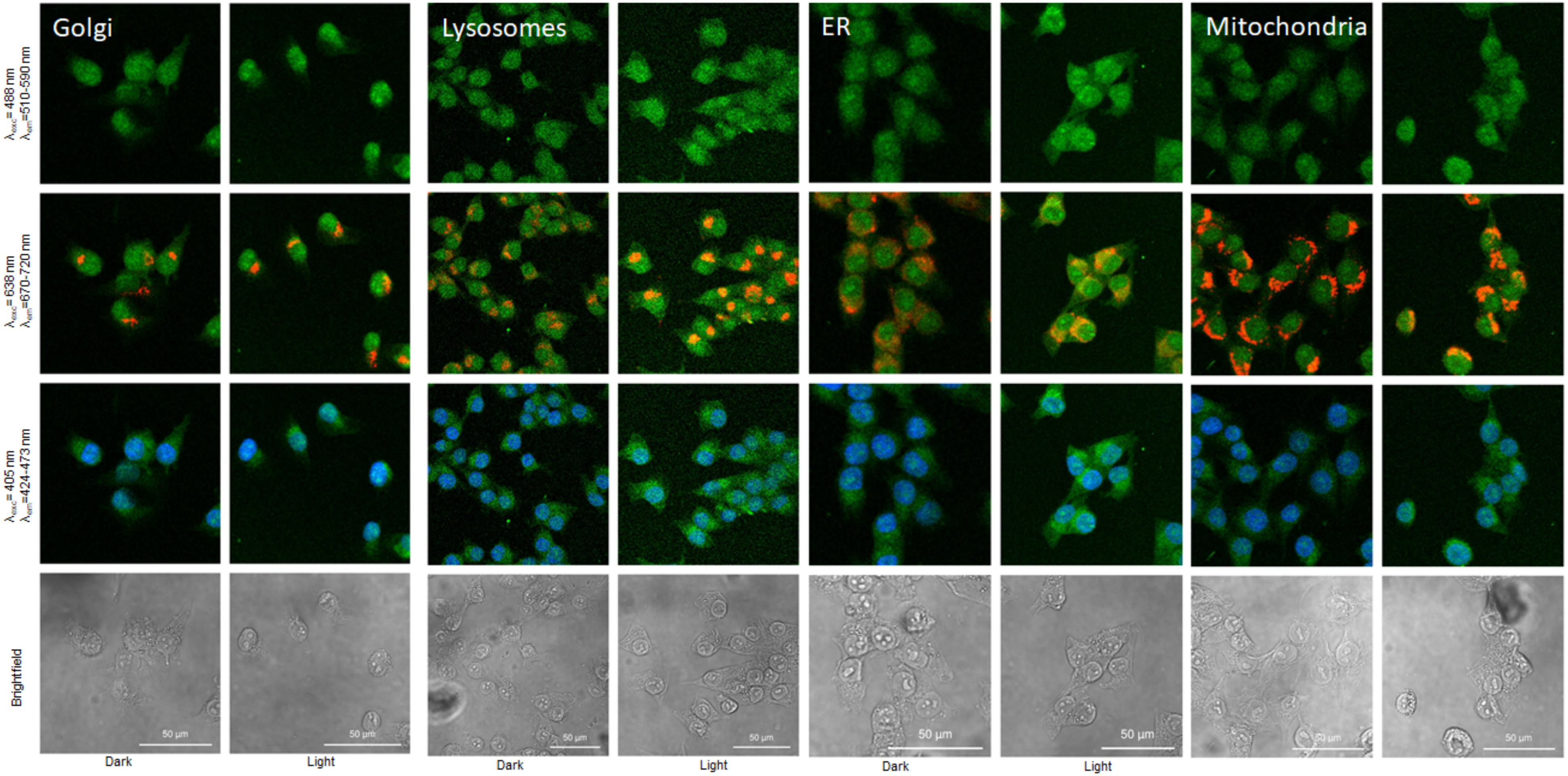

Figure S40: [**2**]Cl_2 in 25 µM concentration; treatment: 60 min irradiation (λ=520 nm, 76 J/cm^2), 0 min incubation post-irradiation. Microscopy images taken with 40x water objective.

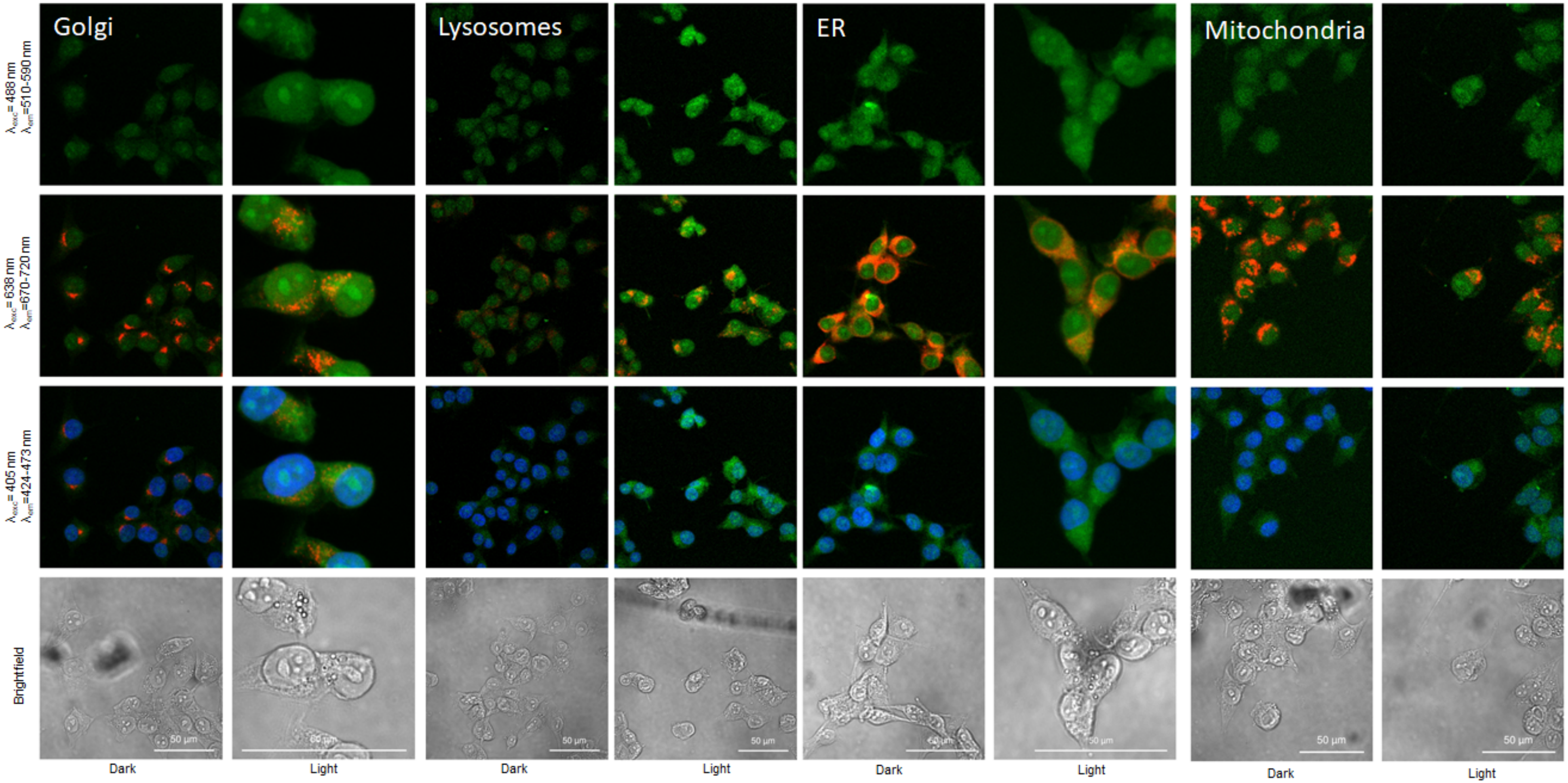

Figure S41: [**2**]Cl_2 in 25 µM concentration; treatment: 60 min irradiation (λ=520 nm, 76 J/cm^2), 60 min incubation post-irradiation. Microscopy images taken with 40x water objective.

9. DNA photointeraction studies

Agarose gel electrophoresis was used to assay the photoinduced binding of pUC19 plasmid using complexes [2]Cl_2 and [4]$(PF_6)_2$. For this purpose, for each metal complex (MC), two gels were run in parallel with the same sample composition but one where the solutions were first irradiated with green light and then loaded on the gel and the other where the solutions were kept in the dark. A 5X tris-boric acid (TBA) buffer (45 mM tris(hydroxymethyl)aminomethane and 45 mM boric acid, pH = 7.4) was used for the gel preparation and for the running buffer. Phosphate buffer (**PB**, 100 mM NaH_2PO_4, pH = 7.0) was used for DNA-MC interactions. A 0.8% w/w agarose gel (0.24 g agarose, 24 g DI H_2O, and 6 mL TBA) was casted using the OWL B1A Easycast system. 40 µL stock solutions for both gels were prepared accordingly to the Table S4. In all samples the pUC19 concentration was kept constant (3 µL, 3 µg, $[DNA\ bp]_{final} = 1.43\cdot10^{-3}$ M), while increasing concentration of MC was used, from 0 to 187.5 µM (**C-H**). For the latter, two stock solutions of 500 µM and 75 µM MC in PB were prepared from a 5 mM stock solution in DMSO. A sample with cisplatin (**B,** $[cisPt]_{final} = 0.1\cdot10^{-6}$ M, 4.7 mM NaCl, 1.7 mM mannitol) and a negative control with the maximum DMSO content, 1.5 µL, 3.75%, that is found in the MC samples (**I**) were also included.

Table S4: Agarose gel electrophoresis stock solution composition.

#	Sample	pUC19 1g/L µL	MC 75 µM µL	MC 500 µM µL	cisPt 1 mM µL	DMSO µL	PB 100 mM µL	Vtot µL	pUC19 final mM	MC final µM	DMSO %
**B**	cisPt	3	-	-	4	-	33	40	1.43	100	0
**C**	400:0	3	-	-	-	-	37	40	1.43	0	0
**D**	400:1	3	2	-	-	-	35	40	1.43	3.8	0.07
**E**	400:5	3	10	-	-	-	27	40	1.43	18.8	0.37
**F**	400:10	3	20	-	-	-	17	40	1.43	37.5	0.75
**G**	400:25	3	-	7.5	-	-	29.5	40	1.43	93.8	1.87
**H**	400:50	3	-	15	-	-	22	40	1.43	187.5	3.75
**I**	DMSO	3	-	-	-	1.5	35.5	40	1.43	-	3.75

Of the 40 µL solutions, 25 µL were transferred into separate wells of 96-well plates and irradiated (520 nm, 30 min, 38 J/cm^2) with the same irradiation setup previously described. The remaining 15 µL were kept in the dark. The two sets of solution were then used for the separate gels. To 15 µL of each solution, 3 µL of 6x loading dye was added. The λ DNA-HindIII digest molecular weight (MW) marker was prepared by adding 9 µL of the DNA MW marker, 51 µL PB, and 13 µL 6x loading dye (**A** and **J**). The gel electrophoresis chamber was filled with 50 mL TBA and 210 mL deionized H_2O. To each well in the gel 12 µL of each sample were loaded. The gel was run at 105 V for 90 min. The gel was stained using 20 µL (10 mg/mL) SYBR Safe (ThermoFisher, product S33102) win 200 mL deionized H_2O for 30 min with slight shaking and then destained in 200 mL deionized H_2O for 20 min. Immediately following destaining, the gel was imaged on a BioRad ChemiDoc imaging system using the SYBR Safe setting, Figure S 42.

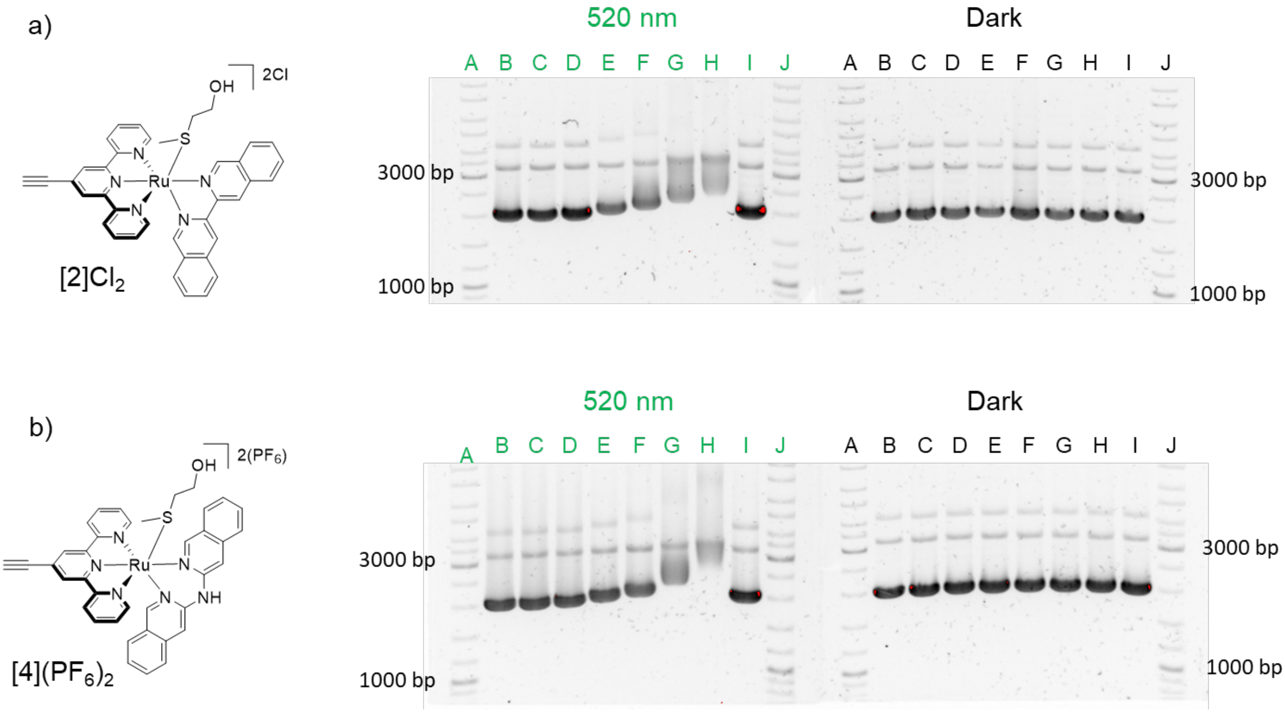

Figure S42: Agarose gel showing the difference of binding of a) [**2**]Cl_2 and b) [**4**]$(PF_6)_2$ to pUC19 upon light irradiation (green lables, 520 nm) and in the dark (black lables, Dark). Samples composition: **A** λ MW marker, **B** cisplatin, **C** DNA control, **D** 400:1 BP:MC, **E** 400:5 BP:MC, **F** 400:10 BP:MC, **G** 400:25 BP:MC, **H** 400:50 BP:MC, **I** DMSO control, **J** λ MW marker.

10. Image Analysis with CellProfiler

10.1. Measurement of cytoplasm /nuclei ratio

Images (acquired as described in Section 8) were analyzed using CellProfiler version 4.2.8. The image analysis workflow proceeded as follows:

1) Nuclei were identified using the *IdentifyPrimaryObjects* module with the global minimum Cross-Entropy thresholding method. Object size constraints were set with a minimum diameter of 7 pixels and a maximum diameter of 100 pixels. This step generated a binary mask representing the nuclei.

2) Whole-cell boundaries were approximated by expanding each nucleus object outward 7 pixels, simulating the cell boundary. The cytoplasm region was then defined by subtracting the nucleus mask from this expanded cell mask, isolating the cytoplasmic area. To improve quantification accuracy, objects exhibiting abnormally high fluorescence intensity in the metal complex fluorescence channel (Alexa488, green) were excluded, as these likely represent imaging artifacts. For all defined objects (nuclei and cytoplasm), mean fluorescence intensities were measured in both the metal complex fluorescence channel (Alexa488, green) and the Alexa647 channel (organelle, red). Subsequently high-intensity areas in both the [green] and [red] channel were identified similarly to the previously performed nuclei segmentation (using the *IdentifyPrimaryObjects* module). These areas are used to calculate the overlap between the green emission from the metal complex and the red emission from each specific organelles. Alexa647 objects (based on specific organelle staining) were used to mask metal complex fluorescence objects. (To circumvent an artifact where ER segmentation included the nucleus the nuclei objects were subtracted from the Alexa 647 objects) The remaining objects were measured for size. The ratio between the total area of Alexa 647objects and the metal complex fluorescence-derived objects represents the fraction of

the organelle object area overlapping with the metal complex fluorescence signal. This fraction indicates how completely the [green] signal colocalizes with the stained organelles.

CellProfiler measurements were exported to R (4.4.1) for further analysis. Cytoplasm/nuclei ratio and fraction of organelle overlap were both calculated in R. P-values were determined on a single cell level for nuclei/cytoplasm ratios and image averages for overlap fraction using Wilcox test (assuming non-normality in ratios as proven by Shapiro-wilk normality test) and t-test (assuming normally distributed means of replicate means), respectively.

Table S5: Relevant CellProfiler parameters

Module index	Module name	Parameter	Value	Relevant inputs
**1**	IdentifyPrimaryObjects	Typical diameter (min;max)	7;100	Nuclei staining image
**1**	IdentifyPrimaryObjects	Threshold strategy	Global	
**1**	IdentifyPrimaryObjects	Thresholding method	Minimum Cross-Enthropy	
**1**	IdentifyPrimaryObjects	Threshold smoothing scale	0	
**1**	IdentifyPrimaryObjects	Correction factor	1	
**1**	IdentifyPrimaryObjects	Lower and upper bounds	0;1	
**1**	IdentifyPrimaryObjects	Size of smoothing filter	0	
**1**	IdentifyPrimaryObjects	Suppress local maxima closer than	4	
**2**	IdentifySecondaryObjects	Method	Distance - N	Nuclei object from #1
**2**	IdentifySecondaryObjects	Number of pixels	7	
**4**	Threshold	Manual threshold	0.9	Complex fluorescence channel image
**4**	Threshold	Threshold smoothing scale	0	
**5**	IdentifyPrimaryObjects	Threshold strategy	Global	Binary mask from #4
**5**	IdentifyPrimaryObjects	Thresholding method	Manual	
**5**	IdentifyPrimaryObjects	Manual threshold	0.5	
**6**	ExpandOrShrinkObjects	Number of pixel by which to expand	2	Objects from #5
**7**	MaskImage			Complex fluorescence channel image & objects from #6
**8**	IdentifyPrimaryObjects	Typical diameter (min;max)	2;20	Complex fluorescence image filtered from #7
**8**	IdentifyPrimaryObjects	Threshold strategy	Adative	
**8**	IdentifyPrimaryObjects	Thresholding method	Robust Background	
**8**	IdentifyPrimaryObjects	Lower outlier fraction	0.93	
**8**	IdentifyPrimaryObjects	Upper outlier fraction	0	
**8**	IdentifyPrimaryObjects	Averaging method	mean	
**8**	IdentifyPrimaryObjects	Variance method	Standard Deviation	
**8**	IdentifyPrimaryObjects	# of deviations	1	
**8**	IdentifyPrimaryObjects	Threshold smoothing scale	1.3488	

**8**	IdentifyPrimaryObjects	Correction factor	0.8	
**8**	IdentifyPrimaryObjects	Lower and upper bounds	0;1	
**8**	IdentifyPrimaryObjects	Size of adaptive window	40	
**8**	IdentifyPrimaryObjects	Size of smoothing filter	10	
**8**	IdentifyPrimaryObjects	Suppress local maxima closer than	7	
**9**	IdentifyPrimaryObjects	Typical diameter (min;max)	2;50	Cy5 image (organelle staining)
**9**	IdentifyPrimaryObjects	Threshold strategy	Adative	
**9**	IdentifyPrimaryObjects	Thresholding method	Robust Background	
**9**	IdentifyPrimaryObjects	Lower outlier fraction	0	
**9**	IdentifyPrimaryObjects	Upper outlier fraction	0	
**9**	IdentifyPrimaryObjects	Averaging method	mean	
**9**	IdentifyPrimaryObjects	Variance method	Standard Deviation	
**9**	IdentifyPrimaryObjects	# of deviations	2	
**9**	IdentifyPrimaryObjects	Threshold smoothing scale	1.3488	
**9**	IdentifyPrimaryObjects	Correction factor	0.8	
**9**	IdentifyPrimaryObjects	Lower and upper bounds	0;1	
**9**	IdentifyPrimaryObjects	Size of adaptive window	100	
**9**	IdentifyPrimaryObjects	Size of smoothing filter	20	
**10**	ExpandOrShrinkObjects	Number of pixel by which to expand	2	Nuclei from #1

[2]Cl$_2$

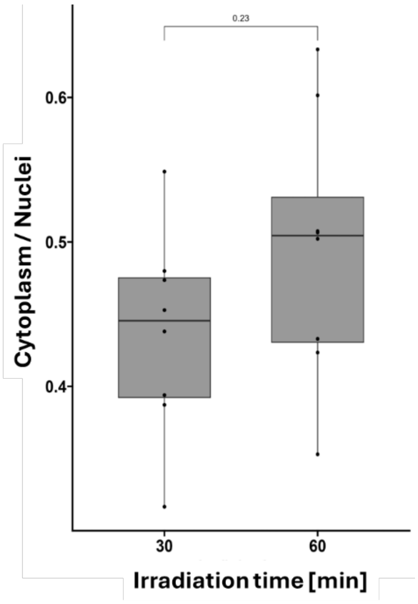

Figure S43: Quantified fluorescence ratio between cytoplasmic and nuclear signal with different light irradiation times and 60 min post-irradiation incubation for complex [**2**]Cl$_2$.

[4](PF$_6$)$_2$

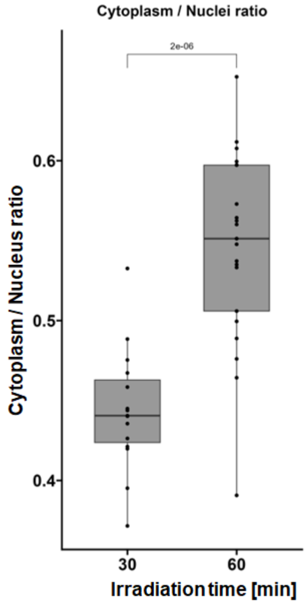

Figure S44: Quantified fluorescence ratio between cytoplasmic and nuclear signal with different light irradiation times and 60 min post-irradiation incubation time for complex [**4**](PF$_6$)$_2$.

10.2. Specific target image analysis

[2]Cl$_2$

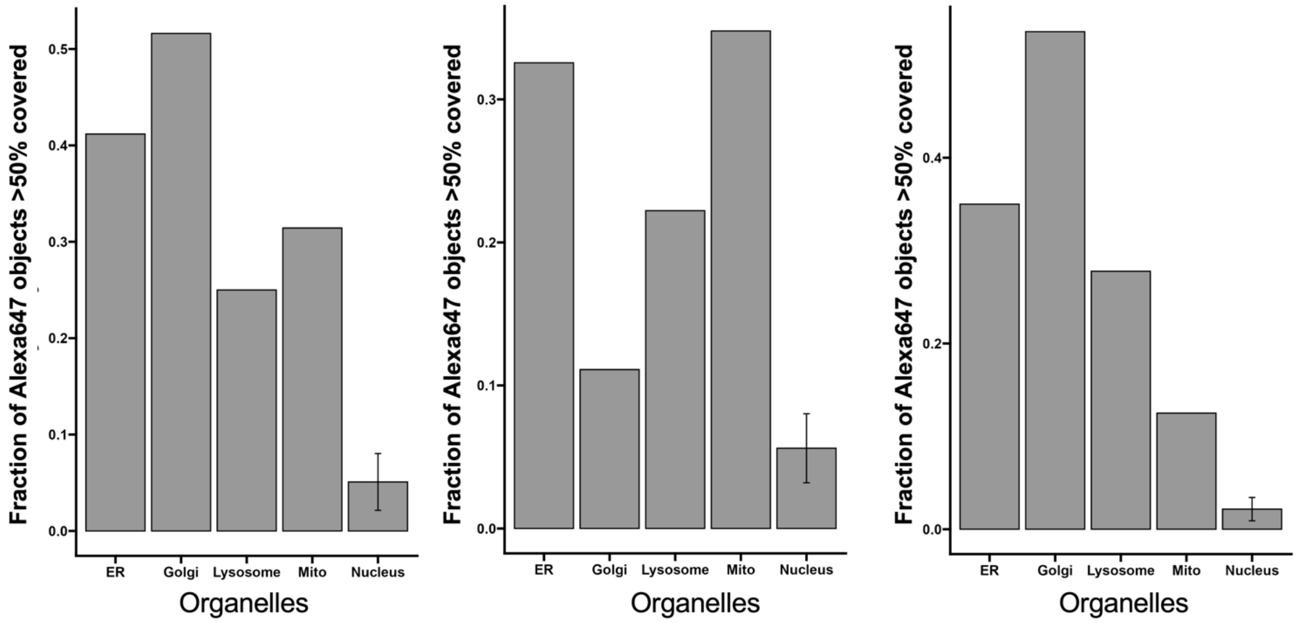

Figure S45: Localization of the tagged complex [**2**]Cl$_2$ through staining of various organelles: ER, Golgi apparatus, Lyosoumes, Mitochondria and Nucleus of the cell. Conditions: Left: 30 min irradiation (38 J/cm^2) followed by 60 min incubation. Center: 60 min irradiation (76 J/cm^2) followed by 0 min incubation. Right: 60 min irradiation (76 J/cm^2) followed by 60 min incubation.

[4]($(PF_6)_2$)

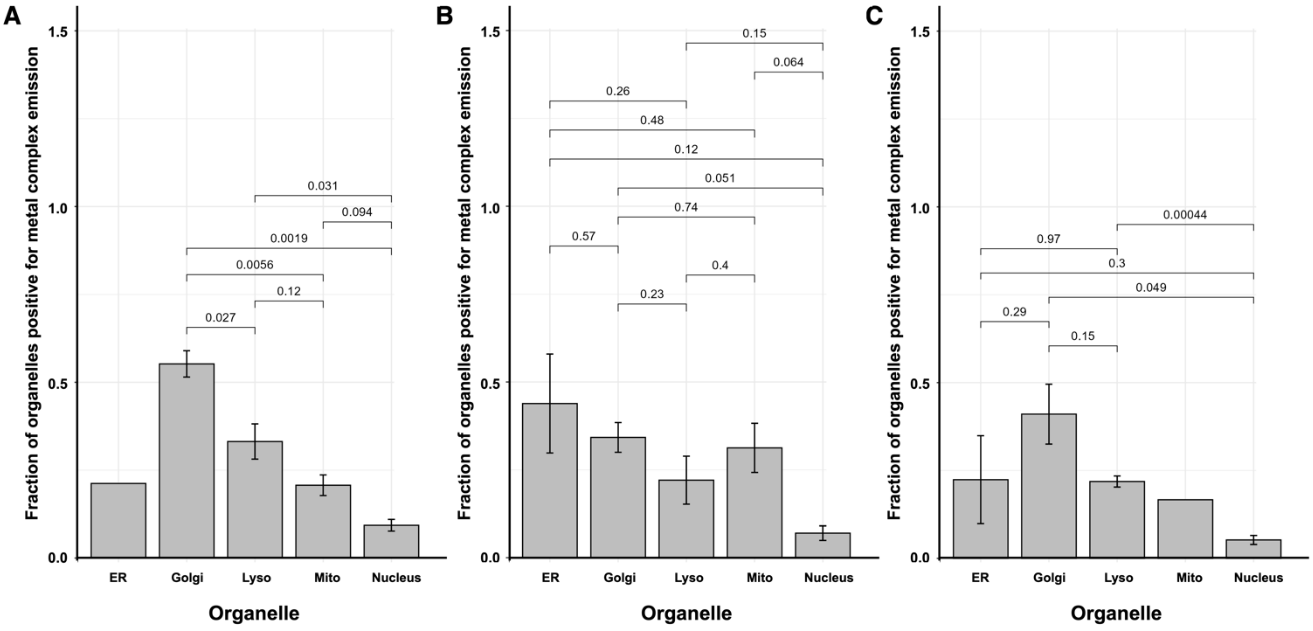

Figure S46: Co-localization of the Alexa488-tagged complex [**4**]$(PF_6)_2$ through staining of various organelles with Alexa647: Endoplasmic reticulum (*ER*), Golgi apparatus (*Golgi*), lysosomes (*Lyso*), mitochondria (*Mito*) and nucleus of the cell. Each organelle was considered positive when >50% of its red emission area overlapped with the green emission from the complex. Conditions: A: 30 min irradiation (38 J/cm^2) followed by 60 min incubation. B: 60 min irradiation (76 J/cm^2) followed by no incubation time (0 min). C: 60 min irradiation (76 J/cm^2), followed by 60 min incubation. Error bars represent standard errors. P-values are included.

11. Dual labelling experiments

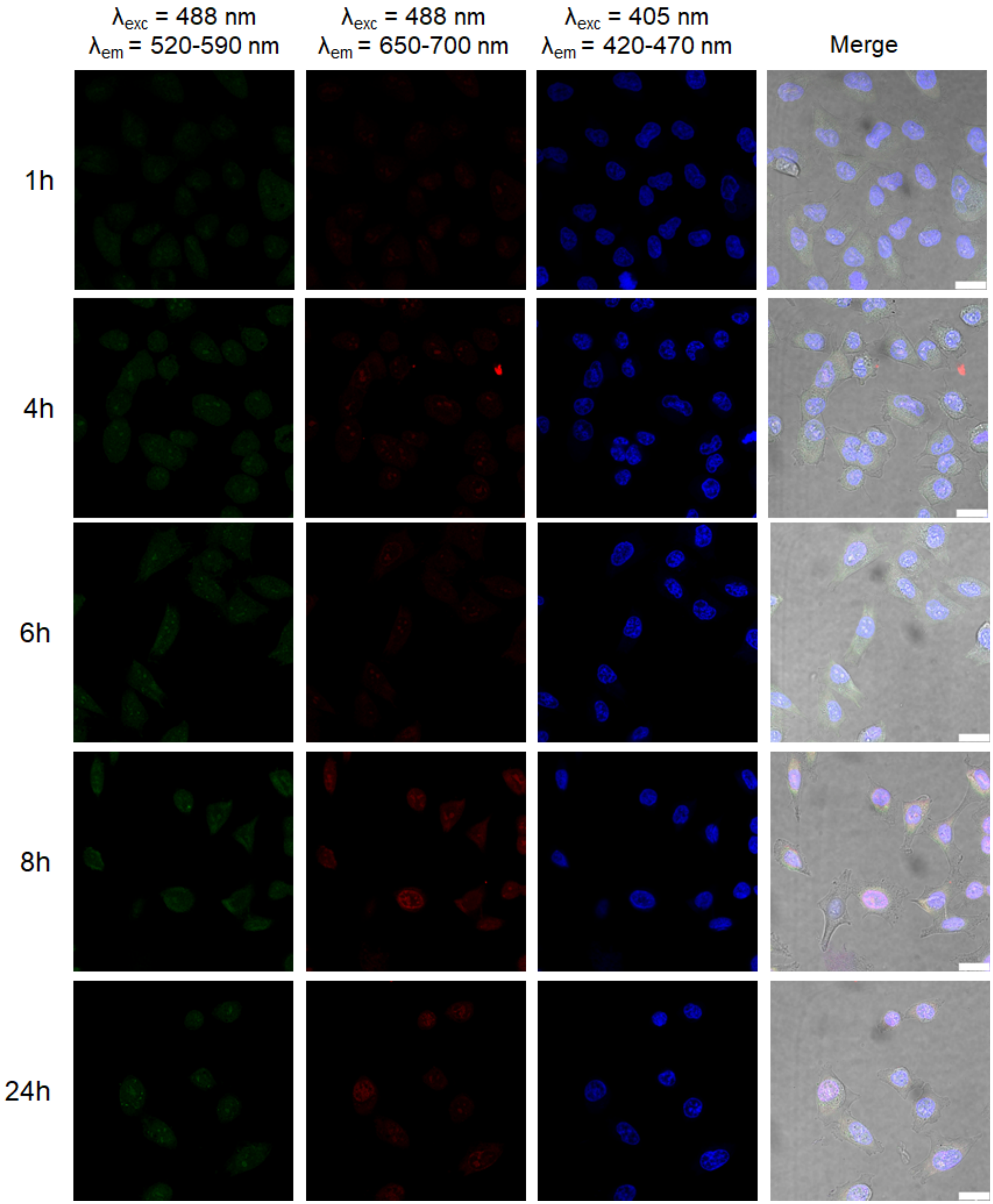

Figure S47: Dual Labelling of CuAAC and IEDDA, [**4**]$(PF_6)_2$ concentration of 5 µM. Column 1 shows CuAAC green emission channel, column 2 shows red emission IEDDA channel, column 3 shows blue emission Hoechst channel, and column 4 shows the merged emission channels and the white contrast

image of the cells. Rows show different incubation times between green light irradiation (λ=520 nm, 36 J/cm^2, 30 min) and fixation of the cells. Scale bar: 20 μm.

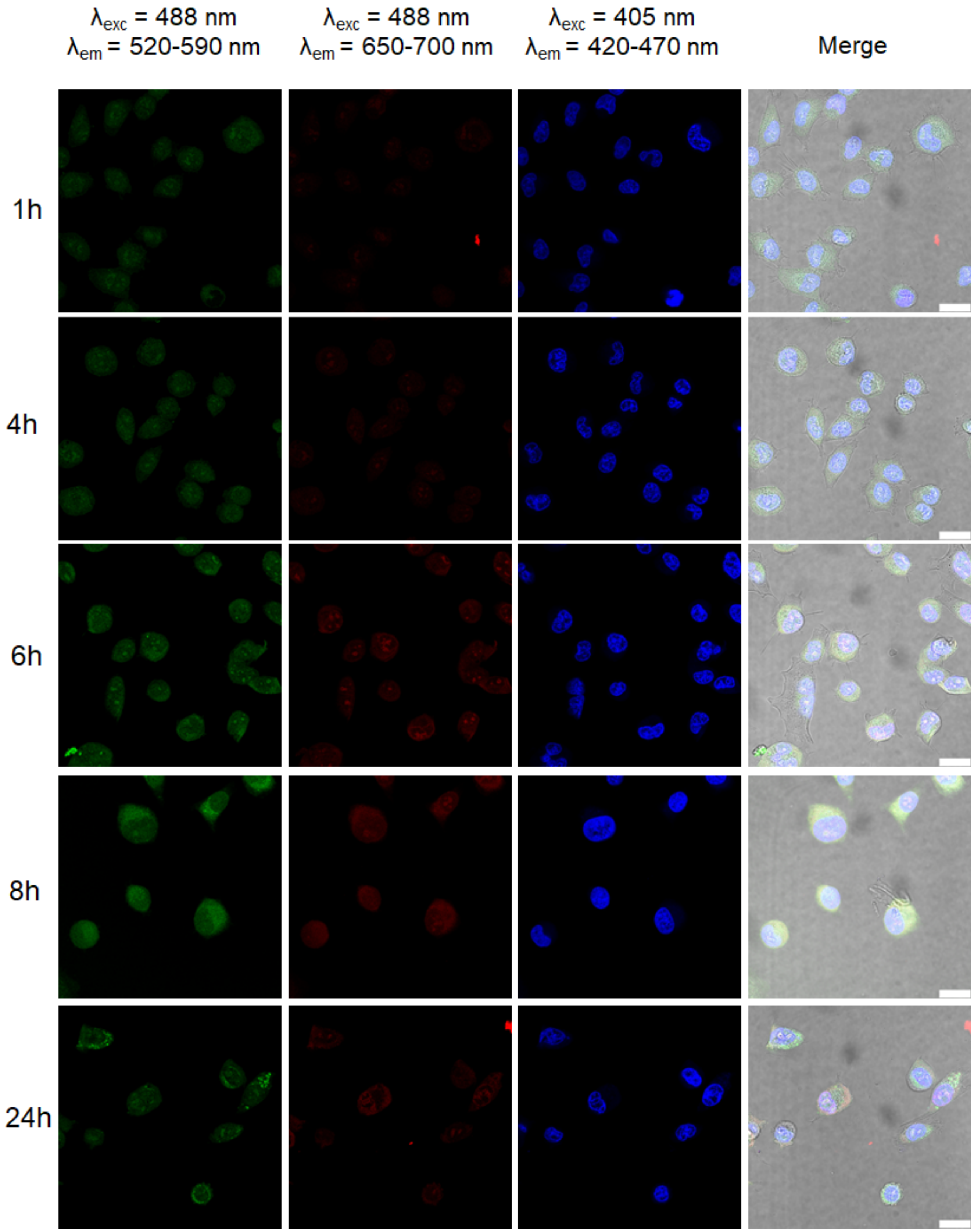

Figure S48: Dual Labelling of CuAAC and IEDDA, [**4**](PF_6)$_2$ concentration of 10 μM. Column 1 shows CuAAC green emission channel, column 2 shows red emission IEDDA channel, column 3 shows blue

emission Hoechst channel, and column 4 shows the merged emission channels and the white contrast image of the cells. Rows show different incubation times between green light irradiation (λ=520 nm, 36 J/cm^2, 30 min) and fixation of the cells. Scale bar: 20 µm.

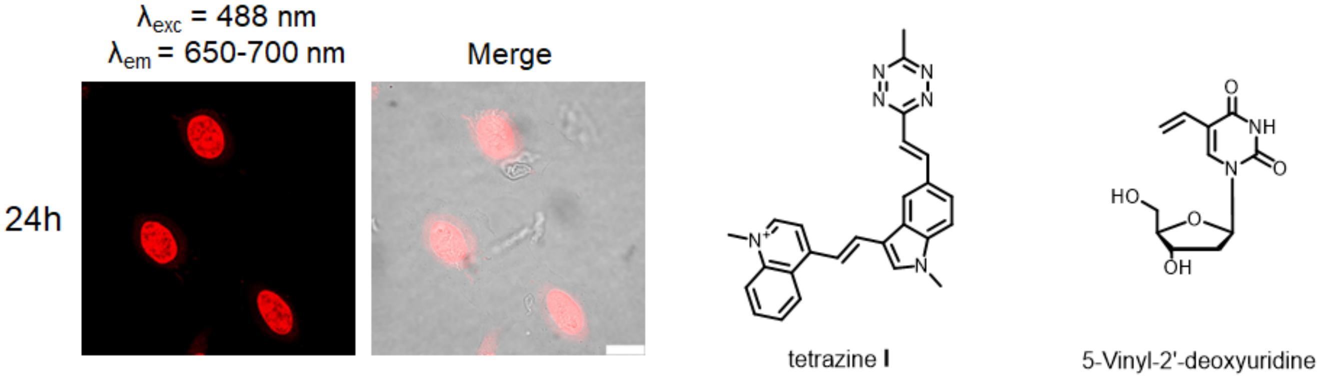

Figure S49: Metabolic labelling of A549 cells with 5-vinyl-2;-deoxyuridine (25 µM, formula shown right) following 24 h incubation time without PACT treatment. Then, cells were fixated with PFA 4%, Triton-X-100 0.5% and BSA 3%, and then labelled with Tetrazine **I** (see formula in the middle) for 6 h incubation time (IEDDA click reaction), before confocal imaging. Scale bar: 20 µm.

12. References

[1] T. Funayama, M. Kato, H. Kosugi, M. Yagi, J. Higuchi, S. Yamauchi, *Bulletin of the Chemical Society of Japan* **2000**, *73*, 1541-1550.

[2] A. Busemann, I. Flaspohler, X. Q. Zhou, C. Schmidt, S. K. Goetzfried, V. H. S. van Rixel, I. Ott, M. A. Siegler, S. Bonnet, *J Biol Inorg Chem* **2021**, *26*, 667-674.

[3] A. Busemann, C. Araman, I. Flaspohler, A. Pratesi, X. Q. Zhou, V. H. S. van Rixel, M. A. Siegler, L. Messori, S. I. van Kasteren, S. Bonnet, *Inorg Chem* **2020**, *59*, 7710-7720.

[4] G. te Velde, F. M. Bickelhaupt, E. J. Baerends, C. Fonseca Guerra, S. J. A. van Gisbergen, J. G. Snijders, T. Ziegler, *Journal of Computational Chemistry* **2001**, *22*, 931-967.

[5] aA. Bahreman, J.-A. Cuello-Garibo, S. Bonnet, *Dalton Transactions* **2014**, *43*, 4494-4505; bL. Bretin, Y. Husiev, V. Ramu, L. Zhang, M. Hakkennes, S. Abyar, A. C. Johns, S. E. Le Devedec, T. Betancourt, A. Kornienko, S. Bonnet, *Angew Chem Int Ed Engl* **2024**, *63*, e202316425.

[6] aX. Q. Zhou, A. Busemann, M. S. Meijer, M. A. Siegler, S. Bonnet, *Chem Commun (Camb)* **2019**, *55*, 4695-4698; bR. Ossola, O. M. Jonsson, K. Moor, K. McNeill, *Chem Rev* **2021**, *121*, 4100-4146.

[7] S. Hopkins, B. Siewert, S. Askes, P. Veldhuizen, R. Zwier, M. Heger, S. Bonnet, *Photochemical & Photobiological Sciences* **2016**, *15*, 644-653.

[8] V. Vichai, K. Kirtikara, *Nat. Protoc.* **2006**, *1*, 1112-1116.

[9] J. Skiba, C. Schmidt, P. Lippmann, P. Ensslen, H.-A. Wagenknecht, R. Czerwieniec, F. Brandl, I. Ott, T. Bernaś, B. Krawczyk, D. Szczukocki, K. Kowalski, *Eur. J. Inorg. Chem*., **2017**, 297-305.
